# Functional Redundancy and Dual Function of a Hypothetical Protein in the Biosynthesis of Eunicellane-Type Diterpenoids

**DOI:** 10.1021/acschembio.4c00413

**Published:** 2024-11-01

**Authors:** Ayesha Ahmed Chaudhri, Yuya Kakumu, Sirinthra Thiengmag, Jack Chun-Ting Liu, Geng-Min Lin, Suhan Durusu, Friederike Biermann, Miriam Boeck, Christopher A. Voigt, Jon Clardy, Reiko Ueoka, Allison S. Walker, Eric J. N. Helfrich

**Affiliations:** † Institute for Molecular Bio Science, Goethe University Frankfurt, Max-von-Laue Strasse 9, 60438 Frankfurt am Main, Germany; ‡ LOEWE Center for Translational Biodiversity Genomics (TBG), Senckenberganlage 25, 60325 Frankfurt am Main, Germany; § Department of Biological Chemistry and Molecular Pharmacology, Harvard Medical School, Boston, Massachusetts 02115, United States; ∥ Synthetic Biology Center Department of Biological Engineering, Massachusetts Institute of Technology, Cambridge, Massachusetts 02139, United States; ⊥ School of Marine Biosciences, Kitasato University, 1-15-1 Kitasato, Minami-ku, Sagamihara, Kanagawa 252-0373, Japan; # Department of Chemistry, Vanderbilt University, 1234 Stevenson Center Lane, Nashville, Tennessee 37240, United States; ¶ Department of Biological Sciences, Vanderbilt University, 465 21st Avenue South, Nashville, Tennessee 37235, United States

## Abstract

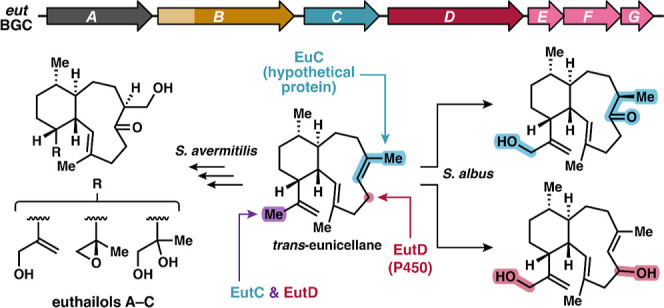

Many complex terpenoids, predominantly isolated from plants and fungi, show drug-like physicochemical properties. Recent advances in genome mining revealed actinobacteria as an almost untouched treasure trove of terpene biosynthetic gene clusters (BGCs). In this study, we characterized a terpene BGC with an unusual architecture. The selected BGC includes, among others, genes encoding a terpene cyclase fused to a truncated reductase domain and a cytochrome P450 monooxygenase (P450) that is split over three gene fragments. Functional characterization of the BGC in a heterologous host led to the identification of several new members of the *trans*-eunicellane family of diterpenoids, the euthailols, that feature unique oxidation patterns. A combination of bioinformatic analyses, structural modeling studies, and heterologous expression revealed a dual function of the pathway-encoded hypothetical protein that acts as an isomerase and an oxygenase. Moreover, in the absence of other tailoring enzymes, a P450 hydroxylates the eunicellane scaffold at a position that is not modified in other eunicellanes. Surprisingly, both the modifications installed by the hypothetical protein and one of the P450s exhibit partial redundancy. Bioactivity assays revealed that some of the euthailols show growth inhibitory properties against Gram-negative ESKAPE pathogens. The characterization of the euthailol BGC in this study provides unprecedented insights into the partial functional redundancy of tailoring enzymes in complex diterpenoid biosynthesis and highlights hypothetical proteins as an important and largely overlooked family of tailoring enzymes involved in the maturation of complex terpenoids.

## Introduction

Terpenoids are the largest and structurally most diverse class of natural products with over 180,000 characterized members that can be grouped into more than 400 structural families.^1^ Terpenoids are found across all domains of life. They are biosynthesized through the joint action of terpene cyclases (TCs) to generate complex hydrocarbon scaffolds and a wide array of tailoring enzymes that decorate the terpene hydrocarbon scaffold.^2−5^ While identical terpene hydrocarbon scaffolds are frequently produced across different biological taxa, varying sets of tailoring enzymes in different organisms result in a vast array of modification patterns. The remarkable structural diversity of terpenoids is reflected in their wide range of bioactivities. Notable examples include the plant-derived artemisinin, which is used as a drug for the treatment of malaria and taxol, an anticancer drug.^6,7^

Terpenoids are biosynthesized from C_5_ isoprene units, isopentenyl pyrophosphate (IPP) and dimethylallyl pyrophosphate (DMAPP), that are condensed by oligoprenyl pyrophosphate synthetases to form linear, achiral polyene pyrophosphates C_5*n*_ (*n* = 1, 2, 3, ...) with methyl branches.^8^ Terpenoids are classified into different families according to the number of the 5-carbon building blocks present in the hydrocarbon scaffold, e.g., monoterpenoids (C_10_), sesquiterpenoids (C_15_), diterpenoids (C_20_), and triterpenoids (C_30_). Despite the limited diversity in biosynthetic precursors (isoprene oligomers), an enormous structural complexity is achieved through the TC-catalyzed cyclization and rearrangement of these achiral polyenes.^9,10^ In many cases, the TC transforms the achiral precursors into highly complex hydrocarbon scaffolds with multiple rings, while changing the hybridization state of up to 50% of carbon atoms and forming multiple stereocenters during the cyclization process.^11^ Additional structural diversity arises from tailoring enzymes such as cytochrome P450 monooxygenases (P450s), flavin-dependent monooxygenases, reductases, dehydrogenases, transferases and isomerases that modify the terpene hydrocarbon scaffold.^12−15^ Among these modifying enzymes, P450s are the most abundant enzymes encoded in terpene biosynthetic gene clusters (BGCs). P450s are responsible for various modifications on terpene scaffolds including hydroxylation, dealkylation, oxidation (alcohols to aldehydes, ketones, or acids), epoxidation, and oxidation-induced rearrangement reactions.^16^

Although terpenoids have traditionally been isolated from plants and fungi, recent advances in genome mining, (i.e., an in silico natural product discovery strategy, in which the potential of an organism to produce natural products is assessed through the analysis of its genome sequence),^17^ and the availability of an ever-increasing number of genome sequences have resulted in the identification of bacteria as an almost untapped treasure trove of biosynthetic blueprints for the production of complex terpenoids.^18^ In contrast to other natural product classes such as the nonribosomal peptides and polyketides, the core structure of a terpene scaffold cannot be predicted, unless a close homologue of the underlying TC has been experimentally characterized.^19^ The modifying enzymes play a more significant role in the process of terpenoid tailoring than in other natural product classes. The type of reaction a tailoring enzyme is performing and the regio and stereochemistry of the broad spectrum of modifying reactions can likewise currently not be predicted.^20^ Another feature of terpenoid biosynthetic pathways is that a single BGC can give rise to dozens of compounds. In some cases, a single TC can transform the same oligoprenyl building block into multiple hydrocarbon scaffolds. Additionally tailoring enzymes can further modify these initial scaffolds through postcyclization reactions, leading to the production of new and distinct hydrocarbon scaffolds.^21,22^ Moreover, the combination of TC and multiple tailoring enzymes frequently results in the production of multiple products with distinct oxygenation patterns.^21^

The targeted isolation of complex bacterial terpenoids can be challenging due to their inimitable properties when compared to members of other natural product classes, for example, the frequent lack of UV-absorbing functional groups, the high volatility of terpene hydrocarbon scaffolds and sparsely functionalized terpenoids (such as C10 and C15 terpenoids), and poor ionization efficiency.^23^ These unfavorable physicochemical properties along with the frequent low production titers in bacteria and the lack of bioinformatic tools for the structural prediction of terpene scaffolds can render the targeted isolation of terpenoids from their natural producer a highly challenging endeavor.

However, recent developments in the fields of genome mining and synthetic biology have now provided the toolset required to retrieve these biosynthetic treasures hidden in bacterial genomes.^24^ The antiSMASH database (a repository of predicted BGCs identified by the genome mining platform antiSMASH) lists more than 25,000 natural product BGCs from only 864 *Streptomyces* strains, which includes more than 4500 terpene BGCs.^25^ The genus *Streptomyces* is well-known for its extremely large and exquisite natural product biosynthetic potential and the products that are associated with the large majority of BGCs in *Streptomyces* have yet to be determined.^26^ We were therefore interested in identifying novel sesqui- and diterpenoids of bacterial origin as unlike many other natural products, sesqui- and diterpenoids show extremely favorable drug-like physicochemical properties (e.g., they frequently follow Lipinsky’s rule of 5).^27^

**Figure 1:**
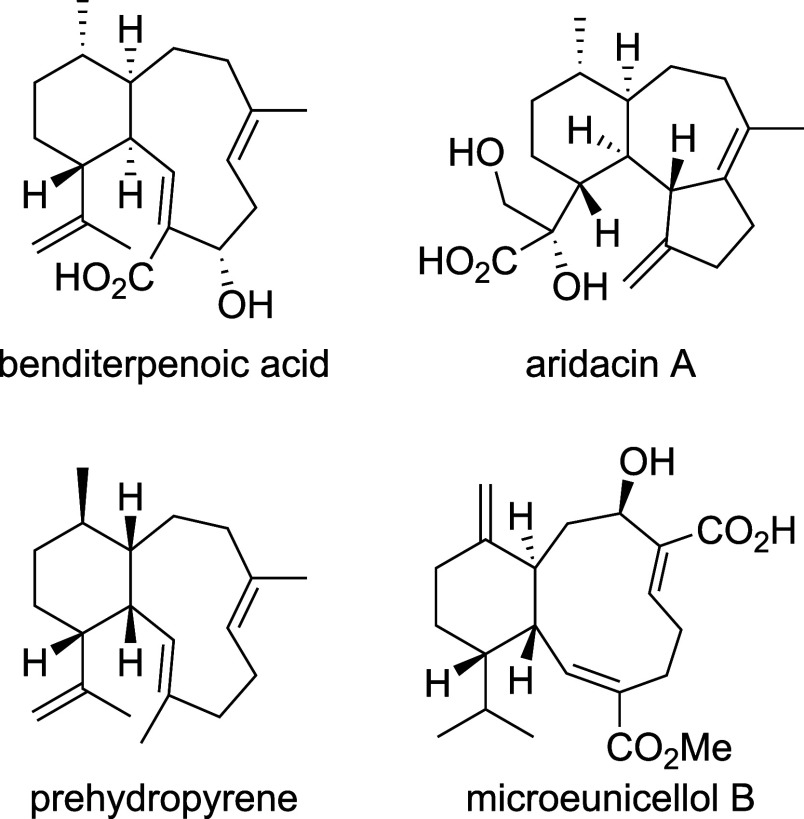
Selection of eunicellane diterpenoids of bacterial origin.

Among the diterpenoids, the eunicellanes constitute a unique family of 6/10 bicyclic hydrocarbon scaffold-containing terpenoids with immense structural complexity due to a diverse array of modification patterns.^28^ Many eunicellane-derived diterpenoids with promising bioactivities have been identified. Notable examples include the soft coral-derived anti-inflammatory klysimplexin R^29^ and eleutherobin, a microtubule inhibitor.^30,31^ To date around 400 eunicellane-derived diterpenoids have been discovered, predominantly from marine organisms (e.g., soft corals).^32^ There are only a handful of reports of eunicellane-type diterpenoids from plants and prokaryotes. However, with the recent advent of genome mining, BGCs encoding TCs responsible for eunicellane-derived diterpenoid biosynthesis have been identified and characterized. Benditerpenoic acid,^33^ aridacins^21^ and prehydropyrene,^34^ a biosynthetic intermediate of hydropyrene, are characterized *cis-*configured bacterial eunicellanes. *Trans*-fused eunicellane scaffolds of bacterial origin are rare and include the microeunicellols that were isolated from a coral symbiont (Figure 1).^35^ The first TC responsible for the biosynthesis of *trans*-configured eunicellanes has only recently been characterized from the albireticulone BGC.^36^

In this work, we combined genome mining and heterologous expression strategies to expand the chemical diversity of eunicellane-type diterpenoids. We mined an in-house bacterial genome sequence database and prioritized a BGC for further characterization based on the presence of genes encoding a TC fused to a truncated reductase domain and multiple tailoring enzymes including a hypothetical protein and cytochrome P450s where one P450 encoding gene is seemingly split into three fragments. We functionally characterized the selected BGC and assigned roles to each gene product, by characterizing the accumulated biosynthetic intermediates. The functional assignment of each gene product in the pathway revealed an unprecedented dual role of a hypothetical protein involved in the isomerization and oxidation of the eunicellane scaffold. Moreover, we provide unexpected insights into the partial functional redundancy of a P450, and the hypothetical protein encoded in the BGC and propose a model for the biosynthesis of the characterized diterpenoids, some of which showed bioactivity against Gram-negative bacteria.

## Results and Discussion

### Genome Mining for Terpene BGCs with Unusual Architecture

We mined the genome sequences of an in-house bacterial genome database for putative talented producers of sesqui or diterpenoids. We prioritized candidate terpenoid producers based on BGC composition and ranked the identified terpene BGCs based on three criteria: (a) unusual biosynthetic features, (b) number of tailoring enzymes and (c) BGC size that would still allow PCR-based cloning of the BGC of interest. Our top hit was a 5.6 kb BGC encoded in the genome of an unassigned *Streptomyces* sp. N2458 that we selected for characterization and further referred to as *eut* BGC. For the taxonomic assignment of the strain harboring the *eut* BGC, we conducted phylogenetic analysis using the Type (strain) Genome Server (TYGS).^37^ This analysis revealed that the *Streptomyces* strain is most closely related to *S. abikoensis* JCM 4002 with a maximum digital DNA–DNA hybridization (dDDH d_4_) value of 64.7%, suggesting that the *Streptomyces* strain is a new species. We named the species *Streptomyces euthainensis* N2458 [eu: from eunicellane, thai: from its country of isolation Thailand] (Figures S1 and S2).

**Figure 2:**
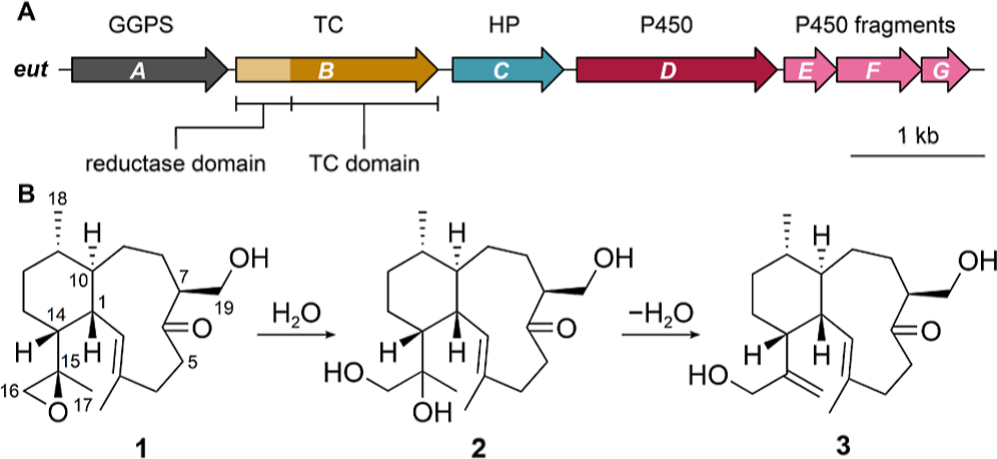
Eunicellane-type diterpenoids produced by heterologous expression of the *eut* BGC in *S. avermitilis* SUKA22. (A) Genomic representation of *eut* BGC. (B) Structures of characterized diterpenoids harboring an epoxide moiety at C-15/16 (**1**), a diol (**2**) that likely results from epoxide ring opening, and an allyl alcohol (**3**) which is the likely product of a dehydration reaction.

The *eut* BGC from *S. euthainensis* N2458 is flanked by a transposase gene on one side and a gene encoding a protein involved in septation on the other side. The GC content of the *eut* BGC (67.3%) is noticeably lower than that of the rest of the genome, which has a GC content of 72.2%. The lower GC content, along with the presence of a transposase gene adjacent to the BGC, suggests that the BGC might have been acquired through horizontal gene transfer. The *eut* BGC harbors seven ORFs and encodes genes for a geranylgeranyl pyrophosphate synthase (GGPS) (EutA), an unusual terpene reductase-cyclase didomain-containing enzyme (EutB) (Figure 2A), and five genes encoding tailoring enzymes. The putative tailoring enzymes include a hypothetical protein (EutC), a full-length cytochrome P450 monooxygenase (EutD), and a P450 that is seemingly split into three individual fragments (EutEFG) (Figure 2A). We verified the BGC sequence, initially obtained by PacBio sequencing, with traditional gene walking by Sanger sequencing to confirm the fusion of the reductase domain with the TC and the unprecedented separation of a single P450 into three gene fragments that are seemingly unidirectional, overlapping and have undergone frame shift (Figures S3 and S4).

Structural predictions of the putative P450 protein complex with AlphaFold-multimer^38^ predicted that the three P450 fragments (EutEFG) form an almost full length P450 monooxygenase that structurally resembles functional P450s (Figure S5A). The structural alignment of the P450 complex (EutEFG) with EutD revealed that the N-terminus present in EutD is absent in EutEFG (Figure S5B). BLAST analysis of the hypothetical protein EutC did not show sequence similarity with any characterized protein and neither the NCBI conserved domain nor PFAM database searches yielded any results. We modeled the putative 3D protein structure using AlphaFold2^39^ (Figure S6) and ran a protein structure comparison on the open-source DALI server^40^ against the protein families present in the Protein Data Bank. The predicted structure of the hypothetical protein shows similarity to the retinaldehyde (diterpenoid) binding protein-1 with a similar ligand binding site. These results suggest that the hypothetical protein is capable of binding (and putatively modifying) diterpenoids.

### Heterologous Expression of the *eut* BGC in an Optimized *Streptomyces* Chassis

To characterize the putative terpenoid BGC, we initially attempted to isolate the terpenoids associated with the *eut* BGC from the native producer. We used different culture conditions, including liquid cultures, agar-based cultures, and cocultures with other bacteria of the strain collection and multiple different media for each approach but no obvious di- or sesquiterpenoid candidates were observed. We next set out to clone the *eut* BGC into the pSET152 plasmid under the control of the strong, constitutive *ermE* promoter (pSET152*) and heterologously express it in the three *Streptomyces* host strains, *S. avermitilis* SUKA22, *Streptomyces albus* J1074 and *S. coelicolor* M1154 (Tables S9 and S10).

To increase the titers of IPP and DMAPP for the formation of the oligoprenyl precursor, we cointroduced a plasmid harboring the mevalonic acid (MVA) pathway. The resulting strains were cultured, extracted and the crude extracts were subjected to high-performance liquid chromatography-high resolution electrospray ionization mass spectrometry (HPLC-HR-ESI-MS) analysis. Comparative metabolomics using the strain harboring *eutAB* and a strain harboring the mevalonate pathway and an empty pSET152* vector (containing pIJ1057_MVA) as control, resulted in the identification of a set of metabolites present in the supernatant of the host strain harboring the *eut* BGC. Low titers of the metabolites were also present in the extracts obtained from the cell pellets of bacterial cultures harboring the *eut* BGC. The three main products associated with the *eut* BGC that were identified in *S. avermitilis* SUKA22 culture extracts had exact masses of *m*/*z* 321.2420 [M + H]^+^ (calcd for *m*/*z* 321.2424, C_20_H_33_O_3_, Δ −0.5 ppm), *m*/*z* 361.2348 [M + Na]^+^ (calcd for *m*/*z* 361.2355, C_20_H_34_NaO_4_, Δ −1.88 ppm), and *m*/*z* 321.2425 [M + H]^+^ (calcd for *m*/*z* 321.2424, C_20_H_33_O_3_, Δ 0.6 ppm) and are further referred to as euthailol A (**1**), euthailol (**2**), and euthailol C (**3**), respectively (Figures S7–S10).

We first focused on the characterization of the three compounds from *S. avermitilis* SUKA22. The crude extract from a 24 L culture was collected and subsequently subjected to two rounds of reverse phase HPLC separation, to purify all three compounds to homogeneity. Analysis of the ^1^H and ^13^C NMR spectra in combination with the 2D NMR spectra (COSY, HSQC, HMBC and NOESY) revealed compounds with a *trans*-fused 6/10-bicyclic hydrocarbon scaffold with an epoxide (**1**), a diol (**2**) and an allyl alcohol (**3**) at C-15/16 in addition to a ketone and a hydroxyl group at C-6 and C-19, respectively (Figures 2B, S11–S28 and Tables S1–S3). Compound **2** likely results from epoxide opening of **1** and **3** is the suggested dehydration product of **2** (Figure 2B). Compound **1** is characterized by a distinct modification pattern with an epoxide at C-15/16 when compared to the previously characterized members of the eunicellane family of diterpenoids. In *S. albus* J1074 and *S. coelicolor* M1154, compounds with an exact mass of *m*/*z* 305.2474 [M + H]^+^ (calcd for *m*/*z* 305.2475, C_20_H_33_O_2_, Δ −0.5 ppm) were identified, indicating an oxygen loss in comparison to euthailols A–C (**1**–**3**) from*S. avermitilis* (Figure S29). The production of compounds detected at *m*/*z* 305.2474 [M + H]^+^ in all heterologous hosts but no production of **1**–**3** in *S. albus* and *S. coelicolor* suggests that either a native P450 of*S. avermitilis* SUKA22 modifies the eunicellane scaffold or one of the P450 in the *eut* BGC is inactive in the other two hosts.

### Characterization of the Didomain TC

A deeper bioinformatic analysis of the unusual terpene reductase-cyclase didomain enzyme revealed that the cyclase domain is fused to a truncated (*E*)-4-hydroxy-3-methyl-but-2-enyl-pyrophosphate (HMBPP) reductase, typically involved in the biosynthesis of DMAPP and IPP. Protein modeling of the didomain enzyme suggests that most catalytically important residues of the HMBPP reductase are not present and that the truncated HMBPP reductase domain could serve a structural role as a molecular lid (Figure S30). The molecular lid might seal the active site to prevent carbocation quenching by nucleophilic attack of water during the cationic cascade reaction that generates the cyclic hydrocarbon backbone. A similar structural role has been suggested previously based on the crystal structures of the aristolochene synthase from *Aspergillus terreus*.^41^ In a recent study, the first *trans*-fused eunicellane cyclase AlbS was characterized from a bacterial source, *Streptomyces albireticuli*.^42^ AlbS forms a rare *trans*-fused 6/10 bicyclic eunicellane scaffold, whose formation has been extensively studied by isotope labeling experiments and quantum chemical calculations.^41^ We heterologously expressed *eutB* in an*Escherichia coli* GGPP-overproducing strain that includes the MVA pathway and a GGPP synthase to increase precursor supply.^43^ Comparison of the NMR data obtained from the terpene hydrocarbon skeleton produced by EutB and the product of AlbS confirmed that EutB is also responsible for the formation of a *trans*-fused 6/10 bicyclic eunicellane scaffold (albireticulene, **9**).

Next, we aimed to investigate the role of the truncated HMBPP reductase domain (HMBPPR) in the formation of the eunicellane hydrocarbon scaffold. A series of heterologous expression strains harboring the full length *eutB* gene, the *eutB*Δreductase domain, *eutB*Δcyclase domain and didomain constructs in which one of the three catalytic motifs of the cyclase domain, respectively, were knocked out (*eutB*ΔNxxxSxxxE, *eutB*ΔWxxxxxRY and *eutB*ΔNxxxSxxxEΔWxxxxxRY) was constructed. Gas chromatography mass spectrometry analysis of the crude extracts of the recombinant strains revealed that in the absence of the reductase domain the production of the hydrocarbon scaffold is reduced by more than 35% (Figure S31), though, no shunt products that would point to water quenching were observed. The increase in eunicellane hydrocarbon scaffold production in the presence of the reductase domain suggests a structural role of the truncated reductase domain.

### Functional Characterization of Tailoring Enzymes Encoded in the *eut* BGC

**Figure 3:**
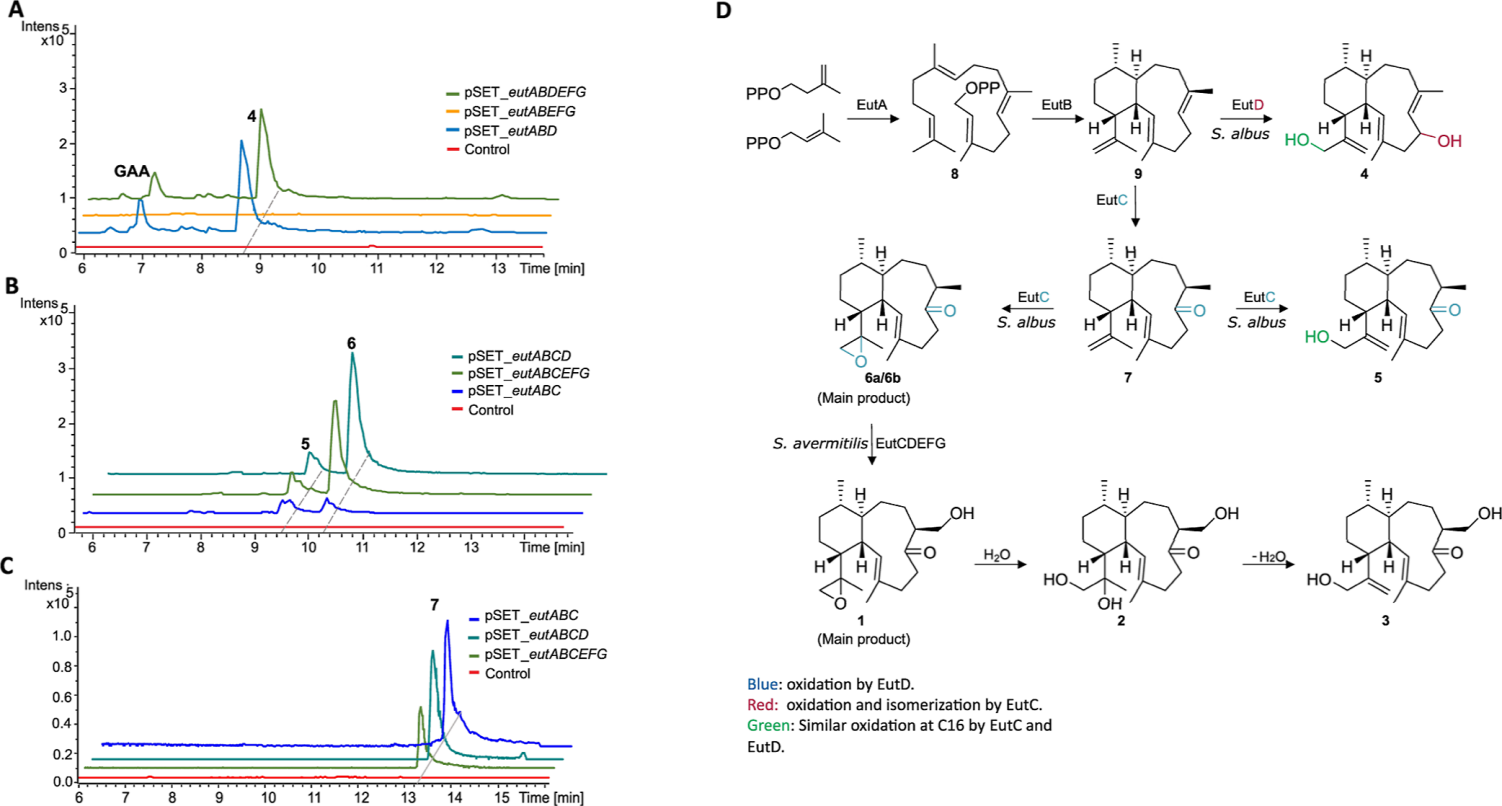
Proposed biosynthesis of euthailols **1**–**7**. (A) Extracted ion chromatograms (EICs) of **4** from *S. albus* harboring plasmids pSET_*eutABD*, pSET_*eutABDEFG* and pSET_*eutABEFG*. (B) EICs of **5** and **6** from *S. albus* harboring pSET_*eutABC*, pSET_*eutABCD* and pSET_*eutABCEFG*. (C) EICs of **7** from *S. albus* harboring pSET_*eutABC*, pSET_*eutABCD* pSET_*eutABCEFG*. *S. albus* strain harboring *eutAB* was used as a control. (D) Proposed biosynthetic model for the formation of **1**–**3**, **4**, **5**, **6**, and **7**.

We next set out to characterize the role of the tailoring enzymes encoded in the *eut* BGC to generate a biosynthetic model of the characterized euthailols A–C (**1**–**3**). Due to the slow growth rate and low production titer in *S. avermitilis* SUKA22 and the potential decoration of the eunicellane scaffold by a host P450, we selected the *S. albus* J1074 chassis for our biosynthetic studies. A series of heterologous expression strains was constructed harboring *eutAB* to generate the hydrocarbon scaffold and genes of the *eut* BGC encoding putative tailoring enzymes, were coexpressed with *eutAB* for functional characterization (Table S9). We first focused on determining the role of the cytochrome P450s and coexpressed *eutDEFG*, *eutEFG* and *eutD*, respectively, with *eutAB* in the IPP-overproducing *S. albus*. No compound with potential modification was detected in the control harboring *eutAB* confirming that no host enzyme is involved in eunicellane hydrocarbon scaffold modification in *S. albus*. In the culture supernatant of *S. albus* strains harboring *eutABDEFG* and *eutABD* two compounds were detected at *m*/*z* 305.2470 [M + H]^+^ (calcd for *m*/*z* 305.2475, C_20_H_33_O_2_, Δ −1.5 ppm) corresponding to a new compound, euthailol D (**4**), and geranylgeranoic acid (GAA) (Figures 3A and S32). We purified euthailol D (**4**) using a combination of open column chromatography, reverse phase preparative and semipreparative HPLC, from a 12 L culture of *S. albus* strains harboring *eutABD* and *eutABDEFG* for subsequent structure elucidation. ^1^H and ^13^C NMR spectra analysis revealed that **4** is likely a shunt product of the euthailol biosynthetic pathway (Figures 3A, S33–S37 and Table S4). The production of **4** was only observed in *S. albus* harboring *eutABD* revealing that only EutD is responsible for the dual oxidation at C-5 and C-16 and that the putative P450 enzyme complex EutEFG is catalytically inactive. The oxidation at C-5 in **4** is, to the best of our knowledge, the first report of a C-5 modification in the eunicellane family of diterpenoids. The unique modification pattern with a C-5 hydroxy in **4** in the absence of the hypothetical protein EutC indicates the putative role of the hypothetical protein in the formation of the ketone at C-6 and/or epoxide at C-16 in **1**–**3**.

To investigate the role of the hypothetical protein EutC we heterologously expressed *eutC, eutCD* and *eutCDEFG*, respectively, in *S. albus* harboring the MVA pathway and *eutAB*. Three compounds were identified by comparative metabolomics using *S. albus* harboring *eutAB* as control. The compounds identified have exact masses of *m*/*z* 305.2473 [M + H]^+^ (calcd for *m*/*z* 305.2475, C_20_H_33_O_2_, Δ −0.5 ppm) (**5**), *m*/*z* 305.2474 [M + H]^+^ (calcd for *m*/*z* 305.2475, C_20_H_33_O_2_, Δ −0.5 ppm) (**6**), *m*/*z* 289.2526 [M + H]^+^ (calcd for *m*/*z* 289.2526, C_20_H_31_O, Δ 0.1 ppm) (**7**), and *m*/*z* 273.2576 [M + H]^+^ (calcd for *m*/*z* 273.2577, C_20_H_32_, Δ 0.2 ppm) (iso-albireticulene **10**), respectively (Figures 3B,C and S38–S41). A 10-fold increase in production of compound **6** was observed when EutC was coexpressed either with EutD, or with EutEFG. Thus, the putative catalytically inactive P450 complex EutEFG might have a structural role in assisting EutC to yield **6**. We purified each compound by open column chromatography followed by preparative and semipreparative HPLC. The production of compound **6** in *S. albus* harboring *eutABC* was not sufficient for structure elucidation and thus **6** was obtained from *S. albus* harboring *eutABCD* and *eutABCEFG*. Structure elucidation of **5**, that we named euthailol E, revealed a ketone at C-6 and a hydroxy group at C-16 of the eunicellane scaffold (Figures 3B, S42–S47 and Table S5). During purification of **6**, two isomers **6a** and **6b** were detected at *m*/*z* 305.2474 [M + H]^+^ (calcd for *m*/*z* 305.2475, C_20_H_33_O_2_, Δ −0.5 ppm). NMR spectral analysis of **6a** and **6b**, that we named euthailol F and G, respectively, revealed the presence of an epoxide ring at C-16 and a ketone at C-6 (Figures S48–S58 and Table S6). Stereochemical characterization determined that **6a** and **6b** are diastereomers of the epoxide at C-15 (Tables S14 and S15). Structure elucidation of **7** that we named euthailol H, revealed the presence of a ketone at C-6 (Figures S59–S64 and Table S7).

During the preparation of this manuscript, a hypothetical protein (AlbU) from *S. albireticuli* has been reported to isomerize the *trans*-eunicellane scaffold to yield *iso*-albireticulene (**10**) that is oxidized by a P450 (AlbP1) to generate 17-hydroxy albireticulone and albireticulone A. The two compounds from *S. albreticuli* show identical modification patterns as **5** and **7**, respectively.^24,36^ However, the careful NMR spectral analysis of **7** in this study suggested that the relative configuration at C-7 of albireticulone A that was initially reported as 7S* has to be revised.^36^ The NOE correlations of H-2/H-7, H-2/H-10, and H-2/H-16 indicated their cofacial relationships, resulting in the stereochemical assignment of C-7 as 7R* for the compound reported in this and the previous study. DFT calculations of ^1^H and ^13^C NMR chemical shifts for (7*R*)-**7** and (7*S*)-**7** in combination with DP4 analysis^44^ support the revision of the relative configuration of C-7 as 7R* (Tables S16 and S17). Furthermore, the production of **5**, **6**, and **7** from *S. albus* harboring *eutABC* confirmed that EutC is responsible for the oxidation at C-6 and C-16. We therefore conclude that EutC first isomerizes the *trans-*eunicellane scaffold (albireticulene, **9**) to yield (iso-albireticulene, **10**) and subsequently acts as an oxygenase to yield **5**, **6** and **7** (Figure 3D). The *in vivo* characterization of EutC is, to the best of our knowledge, the first report of a hypothetical protein that acts both as an isomerase and an oxygenase. We conducted *in silico* analysis to identify a putative cofactor that might assist EutC in oxidizing the isomerized scaffold using AlphaFold 3,^45^ DeepSite,^46^ Consurf^47^ and Prosite.^48^ However, our *in silico* analysis did not result in conclusive evidence for the presence of a specific cofactor or a cofactor less oxidation. As a consequence, *in vitro* studies will be required to determine whether EutC requires a cofactor and to propose a mechanism for the unprecedented isomerase-oxygenase activity of EutC.

We ran a comparative gene cluster analysis of the *eut* BGC with similar gene clusters that harbor genes encoding a TC and a hypothetical protein and aligned the gene clusters with C-linker (Figure S65).^49,50^ EutC showed high sequence similarity with the hypothetical protein (AlbU) encoded in the albireticulone A BGC that was characterized to be exclusively an isomerase lacking oxidation activity. Despite the high degree of similarity with the monofunctional AlbU, EutC exhibits dual function as an isomerase-oxygenase to modify the eunicellane scaffold. This dual functionality suggests a possible functional divergence between EutC and AlbU despite their high levels of similarity.

**Figure 4:**
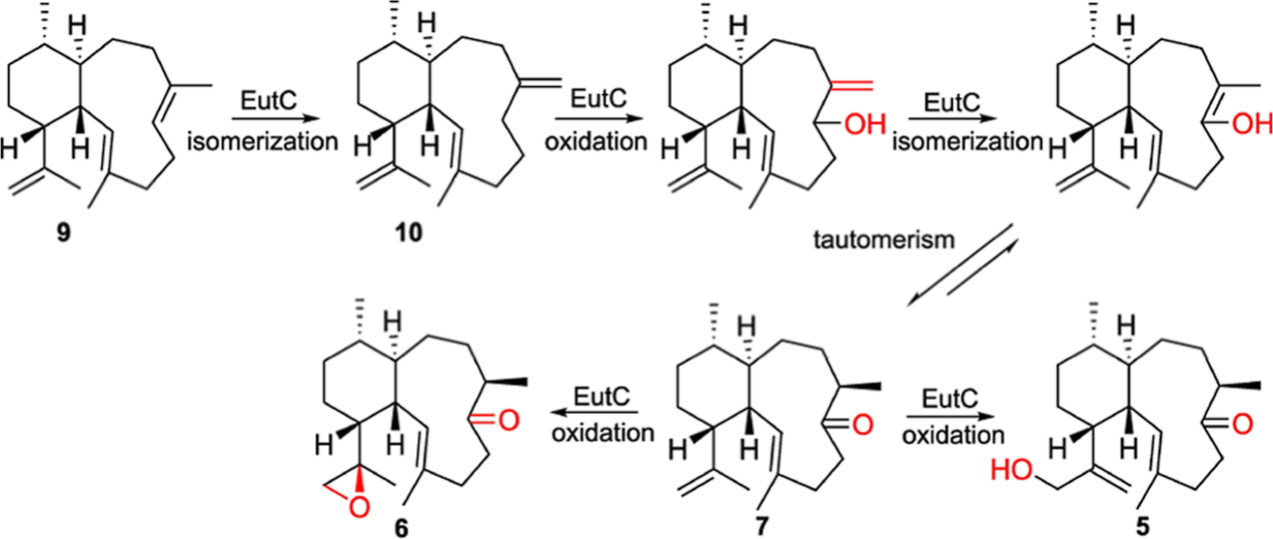
Proposed biosynthetic model for the formation of **5**, **6** and **7**. Successive isomerization-oxidation at C-6 in the eunicellane scaffold by EutC yields **7** that is further oxidized at C-16 to form an epoxide ring in **6** and a hydroxy group in **5**.

**Figure 5:**
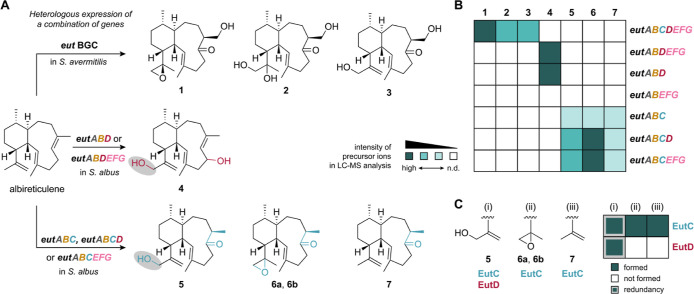
Functional characterization of the euthailol biosynthetic pathway. (A) Expression of complete *eut* BGC and partial *eut* BGC constructs in heterologous hosts *S. avermitilis* and *S. albus*. (B) Heat map representing compound production in strains harboring partial *eut* BGC. (C) Partial functional redundancy observed in EutC and EutD.

Based on the insights gained from our *in vivo* studies, we propose a biosynthetic model for the product of the isomerase-oxygenase EutC where, first the C-6/C-7 double bond shifts to generate an exomethylene at C-7. Subsequently, a hydroxy group is installed at C-6. The putative intermediate undergoes isomerization catalyzed by EutC to form an enol that tautomerizes to the ketone **7**. EutC additionally oxidizes **7** at C-16 resulting in the formation of the epoxide ring in **6** and a hydroxyl group in **5** (Figure 4). The hydroxylation at C-16, observed in **4** and **5** can be catalyzed by two different enzymes encoded in the *eut* BGC, EutC and EutD, respectively (Figure 5). The oxidation of C-16 in **4** and **5** indicates that the *eut* BGC encoded P450 (EutD) exhibits partial functional redundancy with the hypothetical protein (EutC) (Figure 5C).

### Bioactivity Studies

All compounds were screened for potential antibiotic activity against a panel of Gram-positive and Gram-negative bacteria, including all ESKAPE pathogens using disk diffusion assays. MIC values were subsequently determined using the broth microdilution method.^51^ Antifungal activity was tested against the fungal pathogen *Candida albicans*. Antibiotic activity of euthailols **4**, **5**, and **7** was observed against the Gram-positive bacterium *Arthrobacter pascens*, with MIC values of 16, 32, and 16 μg/mL, respectively. Moreover, compound **4**, exhibited weak activity against *Staphylococcus aureus* with an MIC value of 128 μg/mL. In addition, euthailol **4** also showed weak antibacterial activity against the Gram-negative bacteria *Acinetobacter baumannii* and *Enterobacter cloacae*, with MIC values of 64 and 128 μg/mL, respectively. All intermediate products (**4**–**7**) demonstrated low levels of bioactivity against *Pseudomonas aeruginosa*, with MIC values of 64 μg/mL, with the exception of compound **4** which inhibited growth at 32 μg/mL (Table S8). No bioactivity was detected against *C. albicans.* Compounds **1**–**3** did not show any activity in physiologically relevant concentrations against any of the tested bacteria. The low bioactivity of **6a** and **6b** suggests that the presence of the epoxide ring leads to a reduction or suppression of the antibacterial activity while an additional oxidation at C-19 in **1**–**3** results in complete bioactivity loss. Among all seven euthailols identified in this study, 5, 16-dihydroxy euthailol (**4**) exhibited the strongest bioactivity indicating that the C-5 hydroxy might be a crucial element for antibacterial activity of the euthailols.

## Conclusions

The *eut* BGC exhibits several peculiarities when compared to other characterized bacterial eunicellane BGCs, ranging from the unusual dual role of the hypothetical protein as an isomerase and an oxygenase to the unprecedented functional redundancy of the hypothetical protein and the P450 monooxygenase EutD. Surprisingly, despite the sequence similarity between EutD and the first P450 encoded in the *alb* BGC, both enzymes yield different products. EutD is promiscuous and hydroxylates the eunicellane scaffold at positions C-16 and C-5. The latter is a position that has not been reported to be modified in other eunicellanes and is also not modified in the fully maturated euthainols. C-16 can also be modified by the hypothetical protein EutC which installs a unique epoxide moiety at C-15–C-16 which is not present in other fully maturated eunicellanes.

The increase in production of different products dependent on the presence of other biosynthetic enzymes suggests that the biosynthetic enzymes come together to form a large enzyme complex and that the P450 complex has lost its activity while retaining its structural role.

A similar scenario might explain the structural role of the reductase domain suggesting that the characterized BGC is currently in a state of transition and its current form displays a snapshot of its evolutionary trajectory. This hypothesis is further supported by the dual role of the hypothetical protein. The characterized hypothetical protein AlbU in the albireticulone A pathway has been shown to be responsible for the isomerization of an intermediate.^36^ In the case of EutC, the enzyme both acts as an isomerase and an oxygenase suggesting that the enzyme has acquired a catalytic activity, or the hypothetical protein encoded in the *alb* BGC has lost said activity. While the role of the hypothetical protein encoded in the *alb* BGC is split and distributed over two enzymes (P450 and hypothetical protein), EutC is sufficient to catalyze the same types of reactions.

Genome mining for terpene BGCs using the hypothetical protein as bait, revealed that similar hypothetical proteins appear to be present in multiple terpene BGC and are not restricted to eunicellane BGCs. This observation suggests that EutC and its homologues represent a previously understudied family of proteins capable of diversifying terpene hydrocarbon scaffolds beyond the modifications typically introduced by P450s and that the catalytic activities of EutC and its homologues might, at least, be partially overlapping.

In summary, the characterization of an unusual diterpenoid BGC led to the identification of seven members of *trans*-eunicellane derived diterpenoids with unique modification patterns and antibacterial activity.

## Data Availability

The *eut* BGC nucleotide sequence has been deposited in the GenBank database under the accession number PQ240601.

## Supporting Information Available

Experimental methods; NMR and HRMS data; structure elucidation; antimicrobial activity assay; DFT calculations; taxonomic assignment; comparative gene cluster analysis; protein models; Figures S1–S65; Tables S1–S17 (PDF)

## Notes

The *eut* BGC nucleotide sequence has been deposited in the GenBank database under the accession number PQ240601.
