# Supplementary material for: Functional Redundancy and Dual Function of a Hypothetical Protein in the Biosynthesis of Eunicellane-Type Diterpenoids
Source: ACS Chem Biol. 2024 Nov 1;19(11):2314–22. doi: 10.1021/acschembio.4c00413 (PMC11574762; doi:10.1021/acschembio.4c00413)
Supplement: Supplementary file 1 — cb4c00413_si_001.pdf [file cb4c00413_si_001.pdf]

Supporting Information

Functional redundancy and dual function of a hypothetical protein in the biosynthesis of eunicellane-type diterpenoids

Ayesha Ahmed Chaudhri,[1,2] Yuya Kakumu,[1,2,†] Sirinthra Thiengmag,[1,2,†] Jack Chun-Ting Liu,[4,†] Geng-Min Lin,[3] Suhan Durusu,[1] Friederike Biermann,[1,2] Miriam Boeck,[1] Christopher A. Voigt,[3] Jon Clardy,[4] Reiko Ueoka,[5] Allison S. Walker,[6,7] and Eric J. N. Helfrich*[1,2,4]

1. *Institute for Molecular Bio Science, Goethe University Frankfurt, Max-von-Laue Strasse 9, 60438 Frankfurt am Main, Germany*
2. *LOEWE Center for Translational Biodiversity Genomics (TBG) Senckenberganlage 25, 60325 Frankfurt am Main, Germany*
3. *Department of Biological Engineering, Massachusetts Institute of Technology, Cambridge, Massachusetts, USA*
4. *Department of Biological Chemistry and Molecular Pharmacology, Harvard Medical School, Boston, Massachusetts, USA*
5. *School of Marine Biosciences, Kitasato University, 1-15-1 Kitasato, Minami-ku, Sagamihara, Kanagawa, 252-0373, Japan*
6. *Department of Chemistry, Vanderbilt University, 1234 Stevenson Center Lane, Nashville, TN 37240, USA*
7. *Department of Biological Sciences, Vanderbilt University, 465 21st Avenue S, Nashville, TN 37235, USA*

**Email: eric.helfrich@bio.uni-frankfurt.de*

†These authors contributed equally.

Table of Contents

Figure S1	Whole-genome sequence tree generated by the TYGS web server showing relatedness of *Streptomyces euthainensis* to the closest related type strains.	S6
Figure S2	16S rDNA gene sequence-based tree inferred with FastME 2.1.6.1 from GDBP distances by using the TYGS web server.	S7
Figure S3	Nucleotide sequences of *eutB* and amino acid sequence of EutB.	S8
Figure S4	Nucleotide sequences of *eutEFG* and their corresponding protein sequences.	S9
Figure S5	Model of cytochrome P450 complex (EutEFG) created with AlphaFold-multimer.	S10
Figure S6	Predicted structure of the hypothetical protein (EutC) created with AlphaFold 2.	S11
Figure S7	Extracted ion chromatogram of euthailol A (1) and C (3) at *m/z* 321.2424 [M+H]+ of extracts from cultures of *S. avermitilis* SUKA22, *S. coelicolor* M1154*, S. albus* J1074, respectively, that harbor the *eut* BGC.	S12
Figure S8	Isotopic pattern of euthailol A (**1**).	S13
Figure S9	Isotopic pattern of euthailol B (**2**).	S14
Figure S10	Isotopic pattern of euthailol C (**3**).	S15
Figure S11	^{1}H NMR spectrum (499.63 MHz, 303K) of euthailol A (**1**) in CD_3OD.	S16
Figure S12	^{13}C{^{1}H} NMR spectrum (125.64 MHz, 303K) of euthailol A (**1**) in CD_3OD.	S17
Figure S13	^{1}H–^{1}H COSY spectrum (303K) of euthailol A (**1**) in CD_3OD.	S18
Figure S14	HSQC spectrum (303K) of euthailol A (**1**) in CD_3OD.	S19
Figure S15	HMBC spectrum (303K) of euthailol A (**1**) in CD_3OD.	S20
Figure S16	NOESY spectrum (303K) of euthailol A (**1**) in CD_3OD.	S21
Figure S17	^{1}H NMR spectrum (499.63 MHz, 303K) of euthailol B (**2**) in CD_3OD.	S22
Figure S18	^{13}C{^{1}H} NMR spectrum (125.64 MHz, 303K) of euthailol B (**2**) in CD_3OD.	S23
Figure S19	^{1}H–^{1}H COSY spectrum (303K) of euthailol B (**2**) in CD_3OD.	S24
Figure S20	HSQC spectrum (303K) of euthailol B (**2**) CD_3OD.	S25
Figure S21	HMBC spectrum (303K) of euthailol B (**2**) CD_3OD.	S26
Figure S22	NOESY spectrum (303K) of euthailol B (**2**) in CD_3OD.	S27
Figure S23	^{1}H NMR spectrum (499.63 MHz, 303K) of euthailol C (**3**) in CD_3OD.	S28

Figure S24 $^{13}C\{^{1}H\}$ NMR spectrum (125.64 MHz, 303K) of euthailol C (**3**) in CD_3OD. S29

Figure S25 ^{1}H–^{1}H COSY spectrum (303K) of euthailol C (**3**) in CD_3OD. S30

Figure S26 HSQC spectrum (303K) of euthailol C (**3**) in CD_3OD. S31

Figure S27 HMBC spectrum (303K) of euthailol C (**3**) in CD_3OD. S32

Figure S28 NOESY spectrum (303K) of euthailol C (**3**) in CD_3OD. S33

Figure S29 Extracted ion chromatogram of euthailol E (**5**) and F (**6**) at *m/z* 305.2474 $[M+H]^+$ of culture extracts of *S. avermitilis* SUKA22, *S. coelicolor* M1154, and *S. albus* J1074, respectively, that harbor the *eut* BGC. S34

Figure S30 Protein model of the di-domain reductase-cyclase EutB created with AlphaFold 2. S35

Figure S31 Extracted ion chromatogram detected of the *trans*-eunicellane scaffold (albireticulene) at *m/z* 272.20 $[M+H]^+$ from *E. coli* with full length di-domain reductase-cyclase (*eutB*) and knockouts of reductase domain, cyclase domain and catalytic motifs NxxxSxxxE and WxxxxxRY in the cyclase domain. S36

Figure S32 Isotopic pattern of euthailol D (**4**). S37

Figure S33 ^{1}H NMR spectrum (500.18 MHz, 298K) of euthailol D (**4**) in C_5D_5N. S38

Figure S34 $^{13}C\{^{1}H\}$ NMR spectrum (125.78 MHz, 298K) of euthailol D (**4**) in C_5D_5N. S39

Figure S35 ^{1}H–^{1}H COSY spectrum (298K) of euthailol D (**4**) in C_5D_5N. S40

Figure S36 HSQC spectrum (298K) of euthailol D (**4**) in C_5D_5N. S41

Figure S37 HMBC spectrum (298K) of euthailol D (**4**) in C_5D_5N. S42

Figure S38 Isotopic pattern of euthailol E (**5**). S43

Figure S39 Isotopic pattern of euthailol F (**6**). S44

Figure S40 Isotopic pattern of euthailol H (**7**, albireticulone A). S45

Figure S41 Metabolic profile of *S. albus* strains expressing *eutABC* and *eutAB.* S46

Figure S42 ^{1}H NMR spectrum (500.18 MHz, 298K) of euthailol E (**5**) in $(CD_3)_2CO$. S47

Figure S43 ^{1}H NMR spectrum (600.21 MHz, 298K) of euthailol E (**5**) in $CDCl_3$. S48

Figure S44 $^{13}C\{^{1}H\}$ NMR spectrum (125.78 MHz, 298K) of euthailol E (**5**) in $(CD_3)_2CO$. S49

Figure S45 ^{1}H–^{1}H COSY spectrum (298K) of euthailol E (**5**) in $(CD_3)_2CO$. S50

Figure S46 HSQC spectrum (298K) of euthailol E (**5**) in $(CD_3)_2CO$. S51

Figure S47 HMBC spectrum (298K) of euthailol E (**5**) in $(CD_3)_2CO$. S52

Figure S48 ^{1}H NMR spectrum (600.21 MHz, 298K) of euthailol F (**6a**) in $(CD_3)_2CO$. S53

Figure S49	$^{13}C\{^{1}H\}$ NMR spectrum (150.94 MHz, 298K) of euthailol F (**6a**) in $(CD_3)_2CO$.	S54
Figure S50	^{1}H–^{1}H COSY spectrum (298K) of euthailol F (**6a**) in $(CD_3)_2CO$.	S55
Figure S51	HSQC spectrum (298K) of euthailol F (**6a**) in $(CD_3)_2CO$.	S56
Figure S52	HMBC spectrum (298K) of euthailol F (**6a**) in $(CD_3)_2CO$.	S57
Figure S53	NOESY spectrum (298K) of euthailol F (**6a**) in $(CD_3)_2CO$.	S58
Figure S54	^{1}H NMR spectrum (600.21 MHz, 298K) of euthailol G (**6b**) in $(CD_3)_2CO$.	S59
Figure S55	^{1}H–^{1}H COSY spectrum (298K) of euthailol G (**6b**) in $(CD_3)_2CO$.	S60
Figure S56	HSQC spectrum (298K) of euthailol G (**6b**) in $(CD_3)_2CO$.	S61
Figure S57	HMBC spectrum (298K) of euthailol G (**6b**) in $(CD_3)_2CO$.	S62
Figure S58	NOESY spectrum (298K) of euthailol G (**6b**) in $(CD_3)_2CO$.	S63
Figure S59	^{1}H NMR spectrum (600.21 MHz, 298K) of euthailol H (**7**, albireticulone A) in CD_3OD.	S64
Figure S60	$^{13}C\{^{1}H\}$ NMR spectrum (150.94 MHz, 298K) of euthailol H (**7**, albireticulone A) in CD_3OD.	S65
Figure S61	^{1}H–^{1}H COSY spectrum (298K) of euthailol H (**7**, albireticulone A) in CD_3OD.	S66
Figure S62	HSQC spectrum (298K) of euthailol H (**7**, albireticulone A) in CD_3OD.	S67
Figure S63	HMBC spectrum (298K) of euthailol H (**7**, albireticulone A) in CD_3OD.	S68
Figure S64	NOESY spectrum (298K) of euthailol H (**7**, albireticulone A) in CD_3OD.	S69
Figure S65	Comparative gene cluster analysis of *eut* BGC with BGCs encoding for hypothetical proteins with homology to EutC.	S70
Table S1	^{1}H (499.63 MHz) and ^{13}C (125.64 MHz) chemical shifts of euthailol A (**1**) in CD_3OD at 303K.	S71
Table S2	^{1}H (499.63 MHz) and ^{13}C (125.64 MHz) chemical shifts of euthailol B (**2**) in CD_3OD at 303K.	S72
Table S3	^{1}H (499.63 MHz) and ^{13}C (125.64 MHz) chemical shifts of euthailol C (**3**) in CD_3OD at 303K.	S73
Table S4	^{1}H (500.18 MHz) and ^{13}C (125.78 MHz) chemical shifts of euthailol D (**4**) in C_5D_5N at 298K.	S74
Table S5	^{1}H and ^{13}C NMR chemical shifts of euthailol E (**5**) at 298K.	S75
Table S6	^{1}H (600.21 MHz) and ^{13}C (150.94 MHz) NMR chemical shifts of euthailol F (**6a**) and G (**6b**) in $(CD_3)_2CO$ at 298K.	S76

Table S7	^1H (600.21 MHz) and ^{13}C (150.94 MHz) NMR chemical shifts of euthailol H (**7**, albireticulone A) in CD_3OD at 298K.	S77
Table S8	Antibacterial effect of euthailols **4**, **5**, **6a**, **6b**, albireticulone **7**, trimethoprim (Tmp.) and ampicillin (Amp.) against ESKAPE pathogens.	S78
Table S9	Strains used in this study.	S79
Table S10	Plasmids used in this study.	S81
Table S11	Primers used in this study.	S82
Table S12	Comparison of experimental and calculated ^{13}C NMR chemical shifts for euthailol A (**1**).	S84
Table S13	Comparison of experimental and calculated ^1H NMR chemical shifts for euthailol A (**1**).	S85
Table S14	Comparison of experimental and calculated ^{13}C NMR chemical shifts for euthailol F (**6a**) and G (**6b**).	S86
Table S15	Comparison of experimental and calculated ^1H NMR chemical shifts for euthailol F (**6a**) and G (**6b**).	S87
Table S16	Comparison of experimental and calculated ^{13}C NMR chemical shift for euthailol H (**7**, albireticulone A)	S88
Table S17	Comparison of experimental and calculated ^1H NMR chemical shifts for euthailol H (**7**, albireticulone A)	S89
Experimental Methods		S90

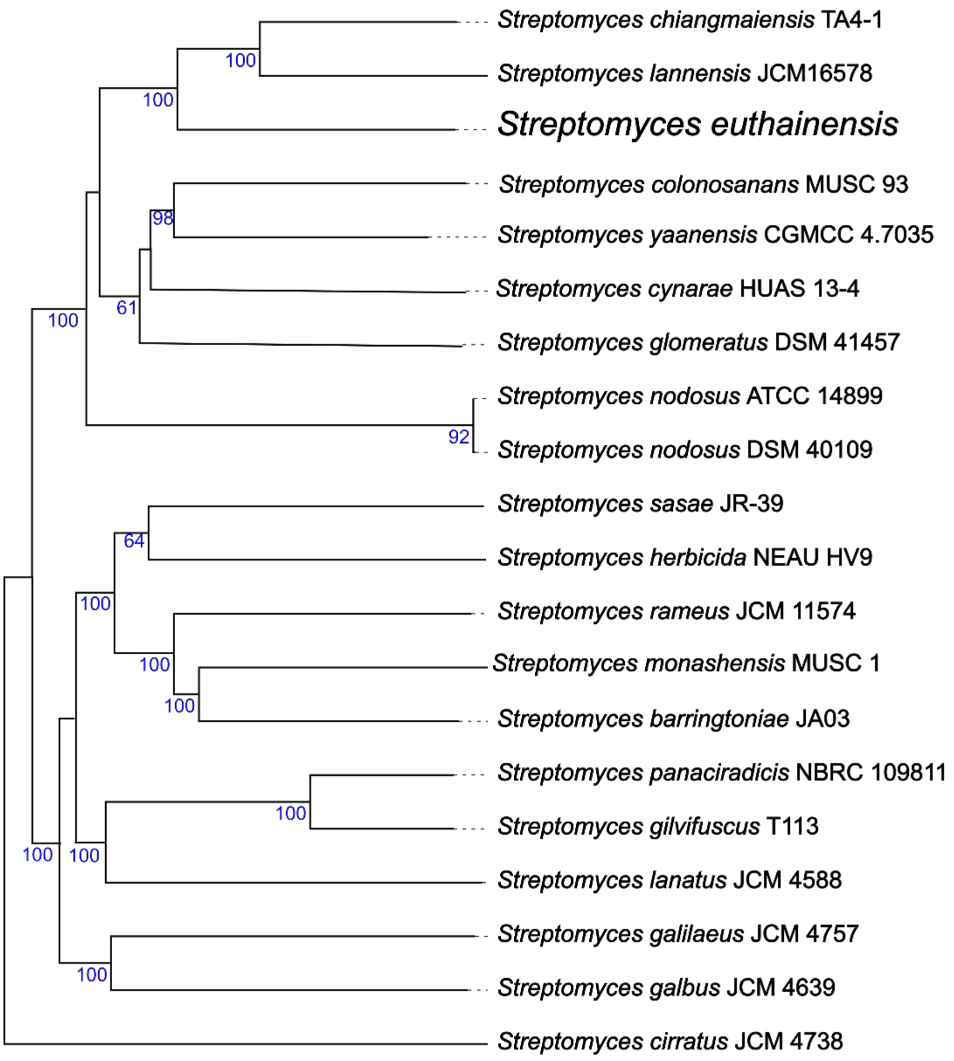

Figure S1. Whole-genome sequence tree generated by the TYGS web server showing relatedness of *Streptomyces euthainensis* N2458 to the closest related type strains. The branch lengths are scaled in terms of Genome BLAST Distance Phylogeny (GBDP) distance formula d_5. The numbers represent GBDP pseudo-bootstrap support values > 60 % from 100 replications, with an average branch support of 87.7 %.

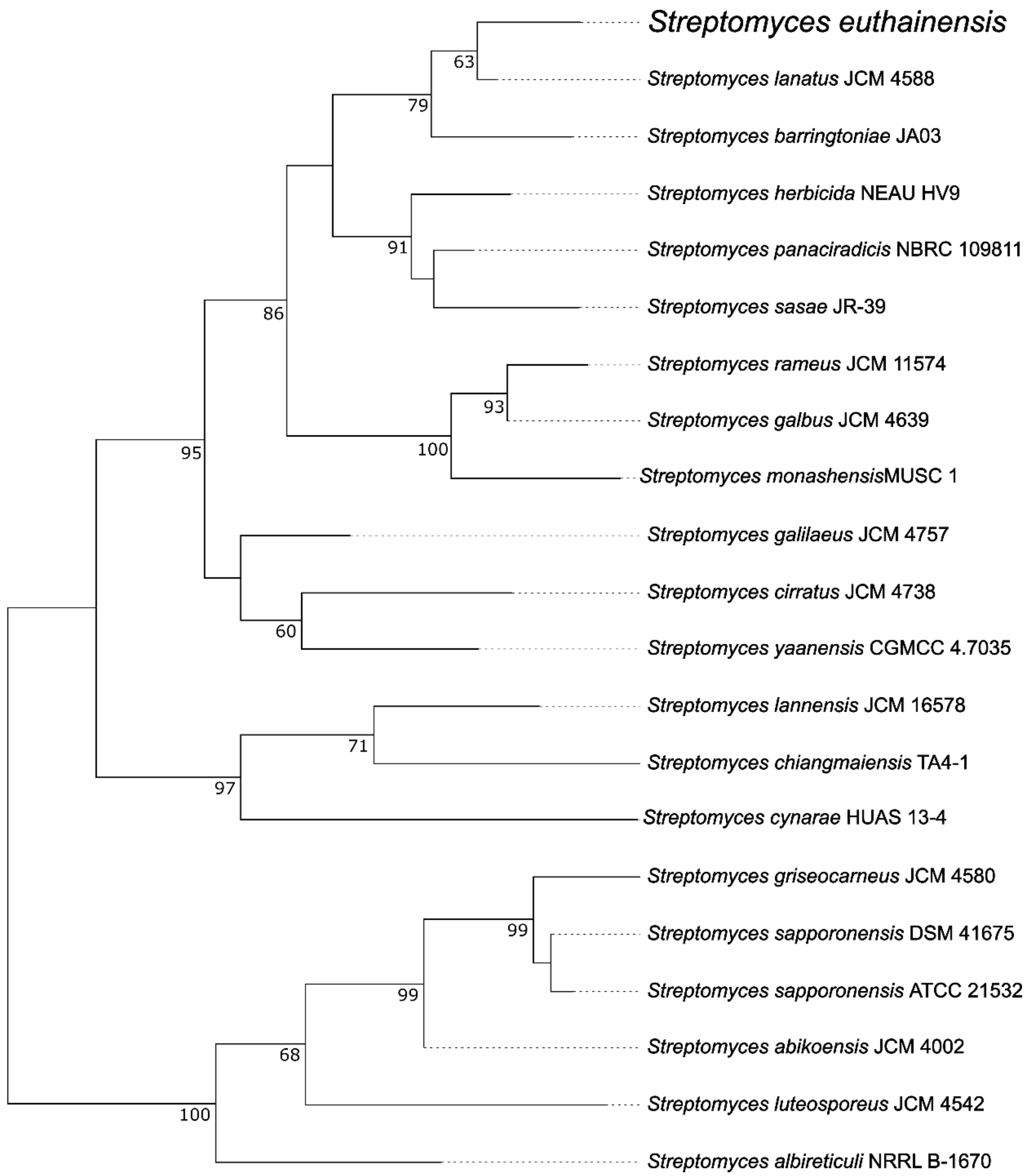

Figure S2. 16S rDNA gene sequence-based tree inferred with FastME 2.1.6.1 from GDBP distances calculated from 16S rDNA gene sequences. The branch lengths are scaled in terms of GBDP distance formula d_5. The numbers represent GBDP pseudo-bootstrap support values > 60% from 100 replications, with an average branch support of 77.1 %.

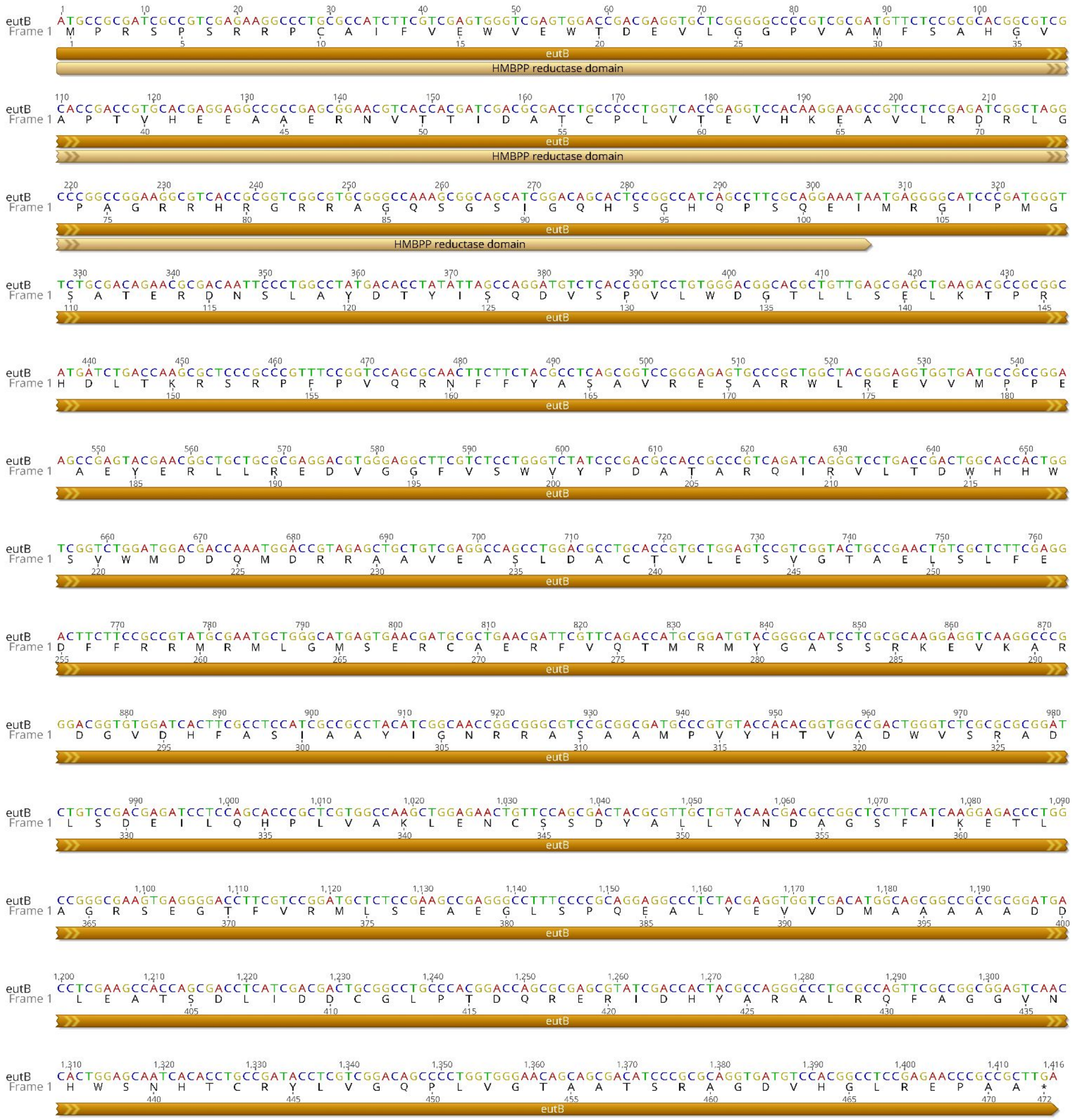

Figure S3: Nucleotide sequences of *eutB* and amino acid sequence of EutB. The sequencing results confirmed that the HMBPP reductase domain is fused with the terpene cyclase at the N-terminus.

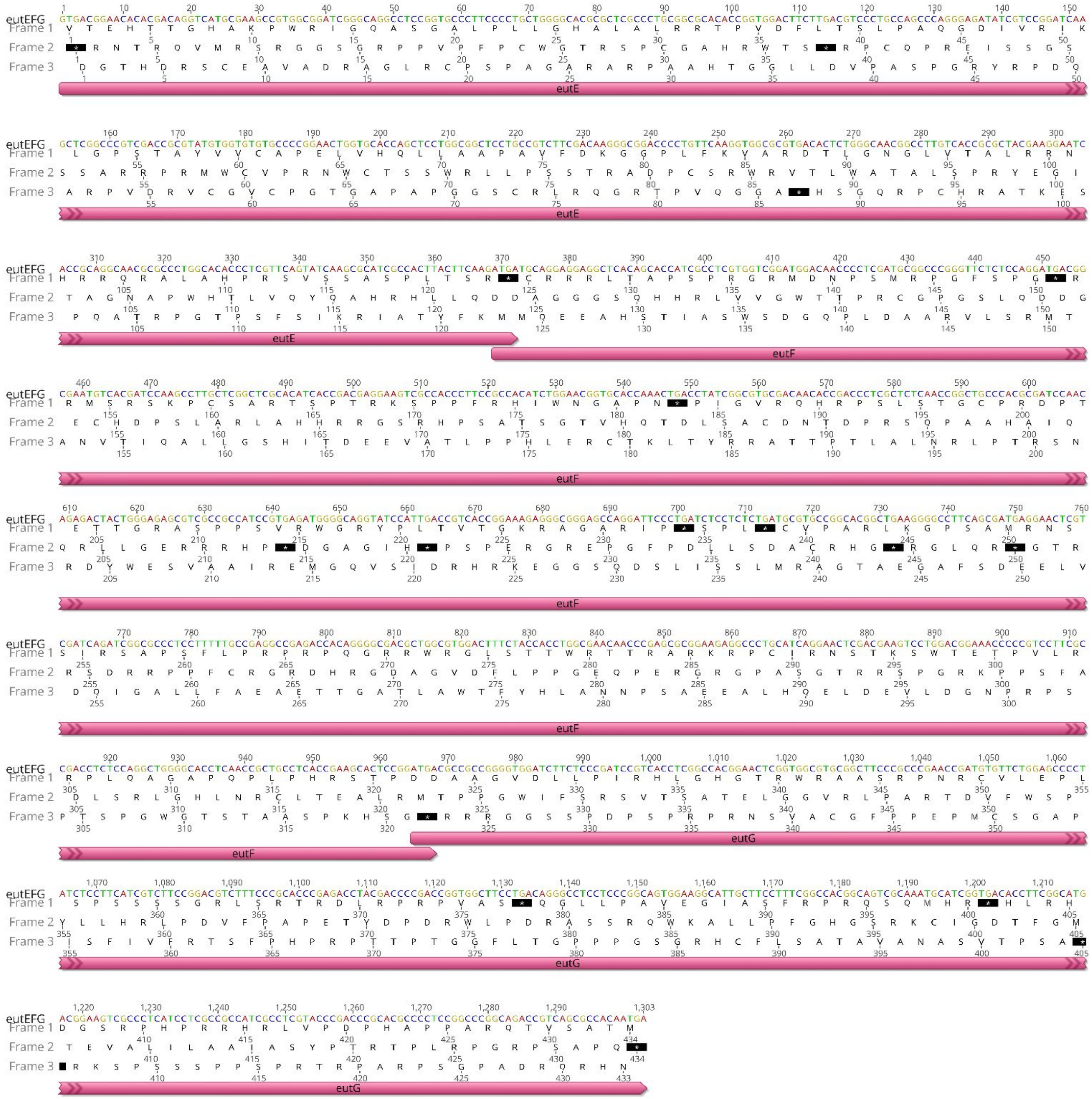

Figure S4: Nucleotide sequences of *eutEFG* and their corresponding protein sequences. The sequencing results confirmed that the three P450s gene fragments (*eutEFG*) are partially overlapping and translated in different reading frames (frame 1, 3 and 2, respectively).

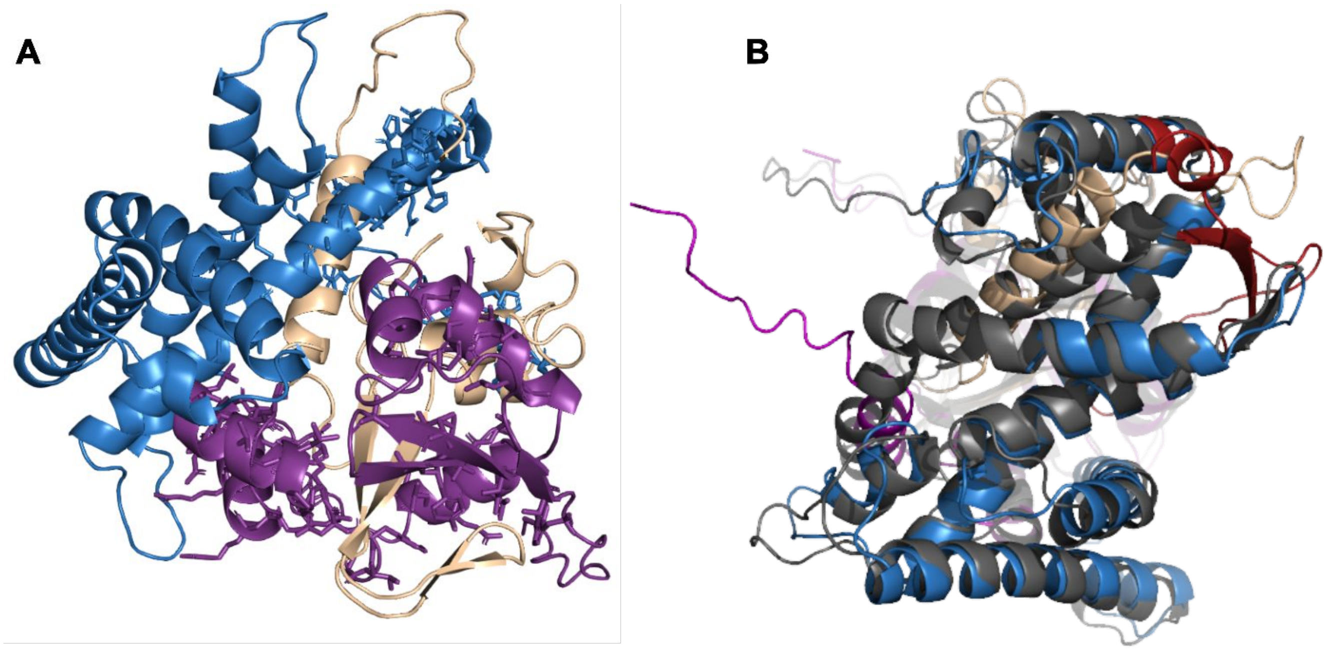

Figure S5: Model of cytochrome P450 complex (EutEFG) created with AlphaFold-multimer. A. EutE, F, G are depicted in beige, blue and lilac, respectively. B. EutEFG superimposed onto the alphafold2 model of EutD. The N-terminus present in EutD that is missing in the EutEFG complex is depicted in red.

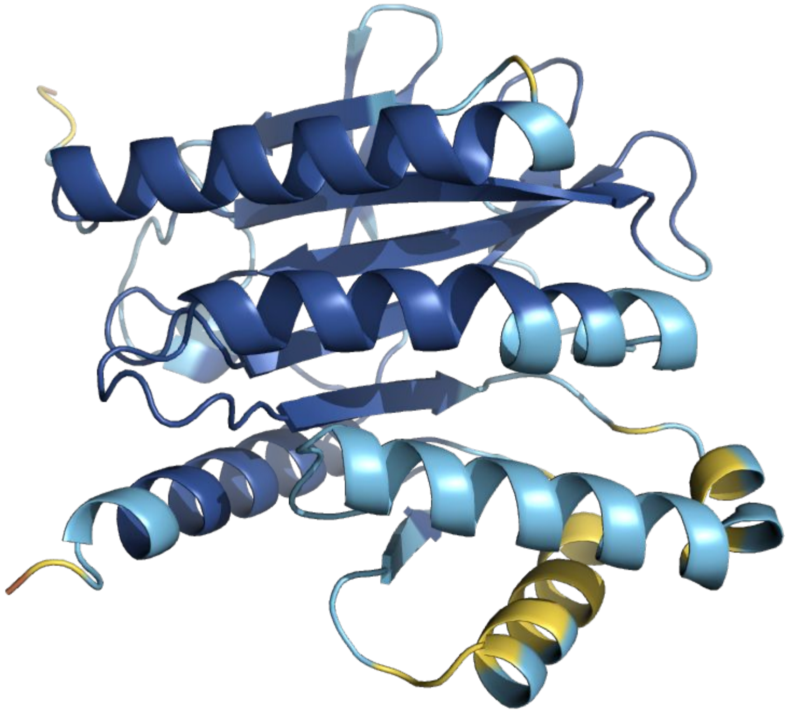

Figure S6: Predicted structure of the hypothetical protein (EutC) created with AlphaFold 2. Regions of low confidence (70 > pLDDT > 50) are shown in yellow, regions of high confidence (90 > pLDDT > 70) are shown in light blue and regions of very high confidence (pLDDT> 90) are shown in dark blue.

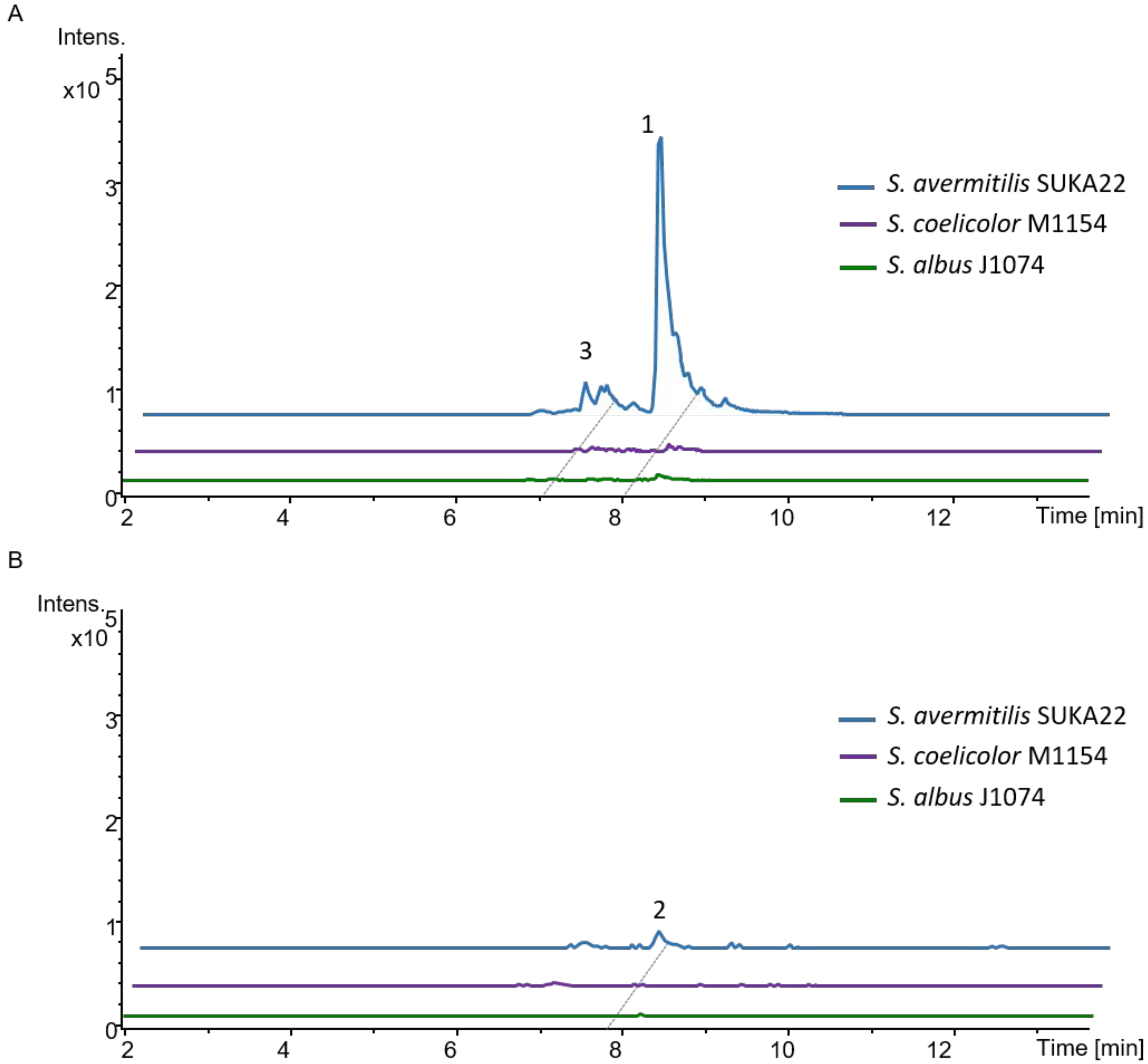

Figure S7: Extracted ion chromatogram (EIC) of euthailol A, B and C from culture extracts of *S. avermitilis* SUKA22*, S. coelicolor* M1154, *S. albus* J1074, respectively, that harbor the *eut BGC.* A. EIC of euthailol A (**1**) and C (**3**) at *m/z* 321.2424 $[M+H]^+$, B. EIC of euthailol B (**2**) at *m/z* 361.2348 $[M+Na]^+$.

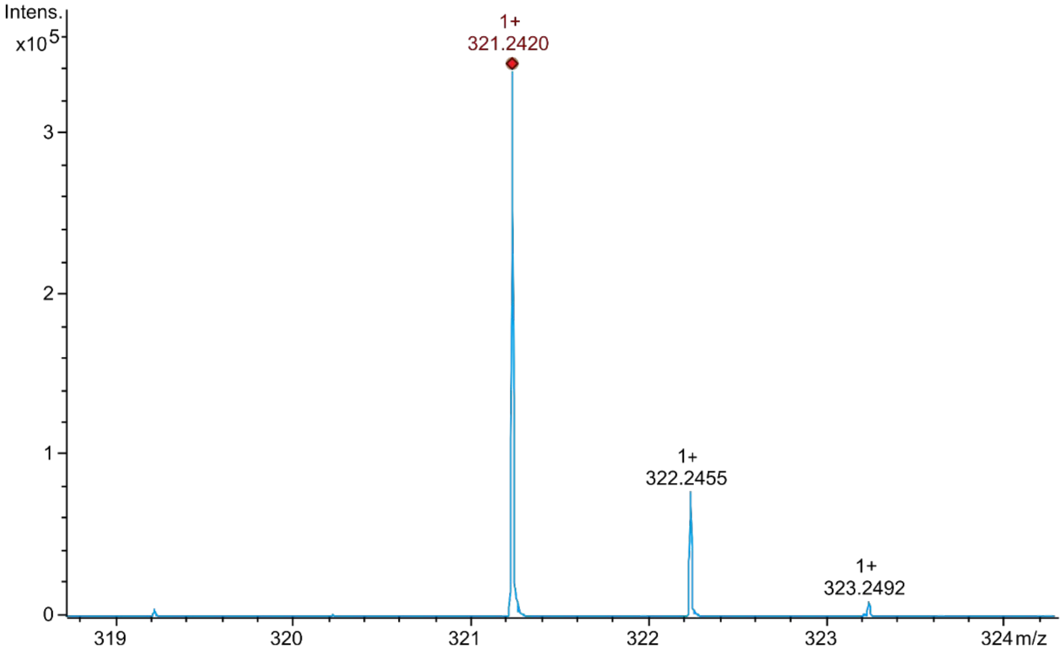

Fig S8: Isotopic pattern of euthailol A (**1**).

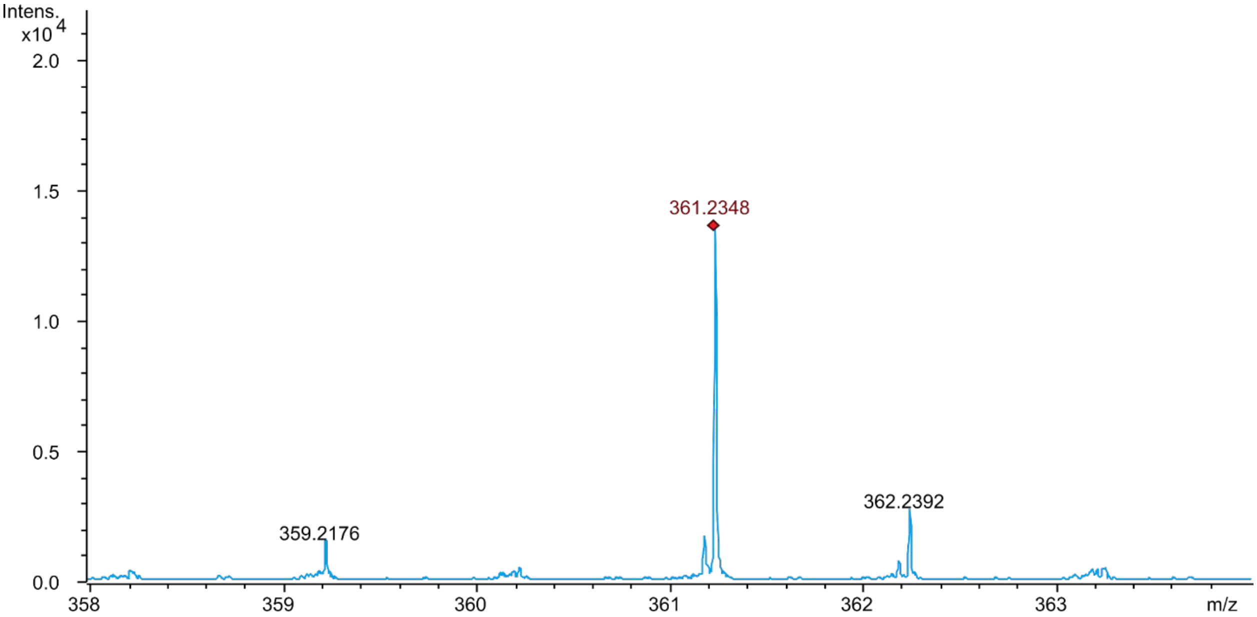

Fig S9: Isotopic pattern of euthailol B (**2**).

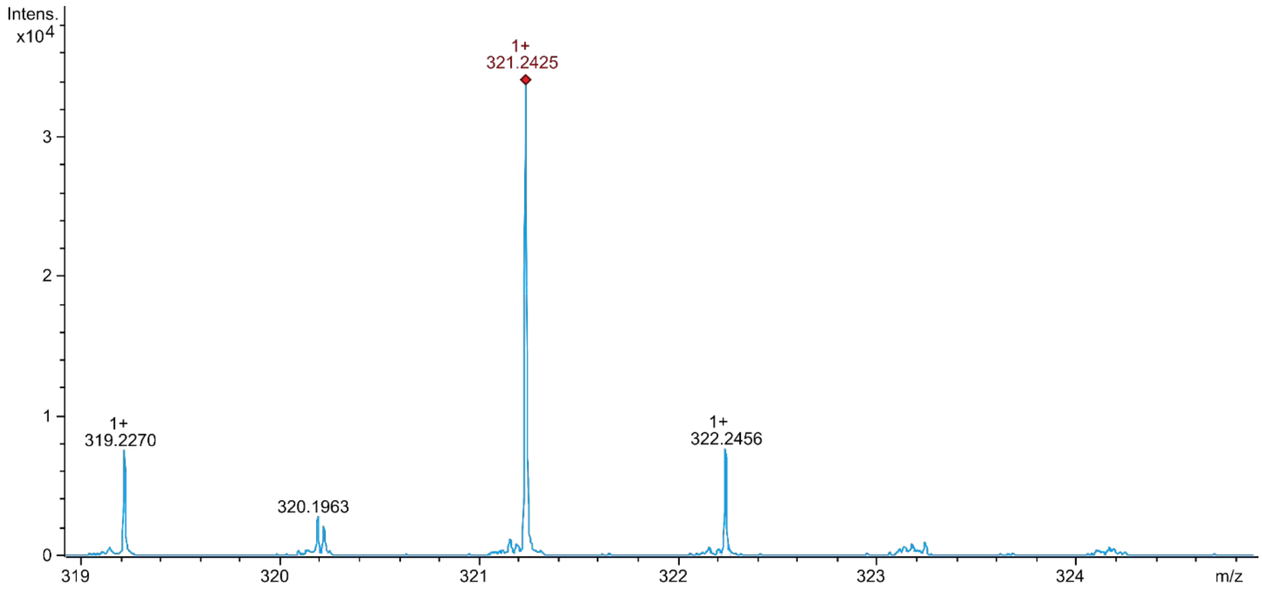

Fig S10: Isotopic pattern of euthailol C (**3**).

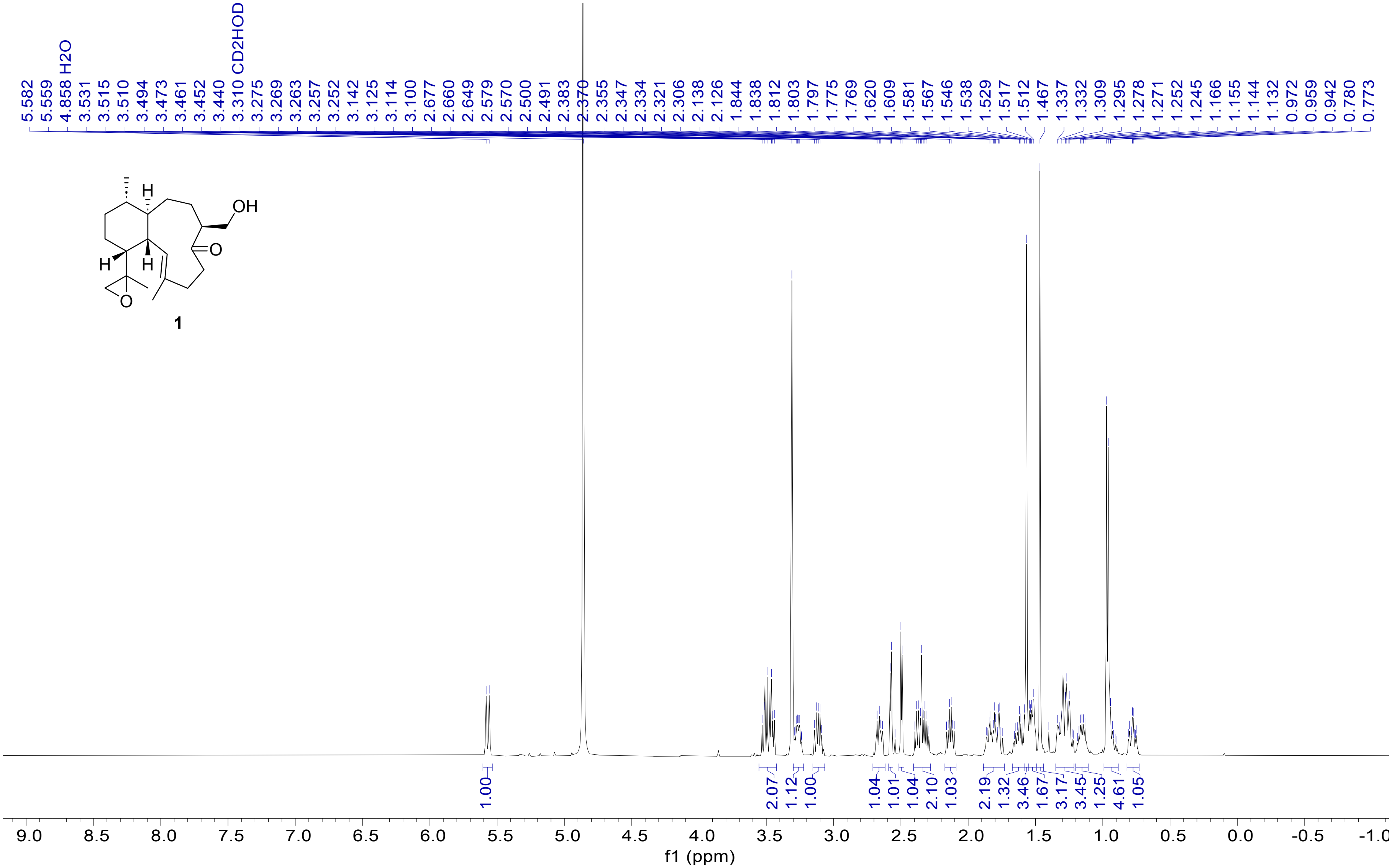

Figure S11. ^{1}H NMR spectrum (499.63 MHz, 303K) of euthailol A (**1**) in CD_3OD.

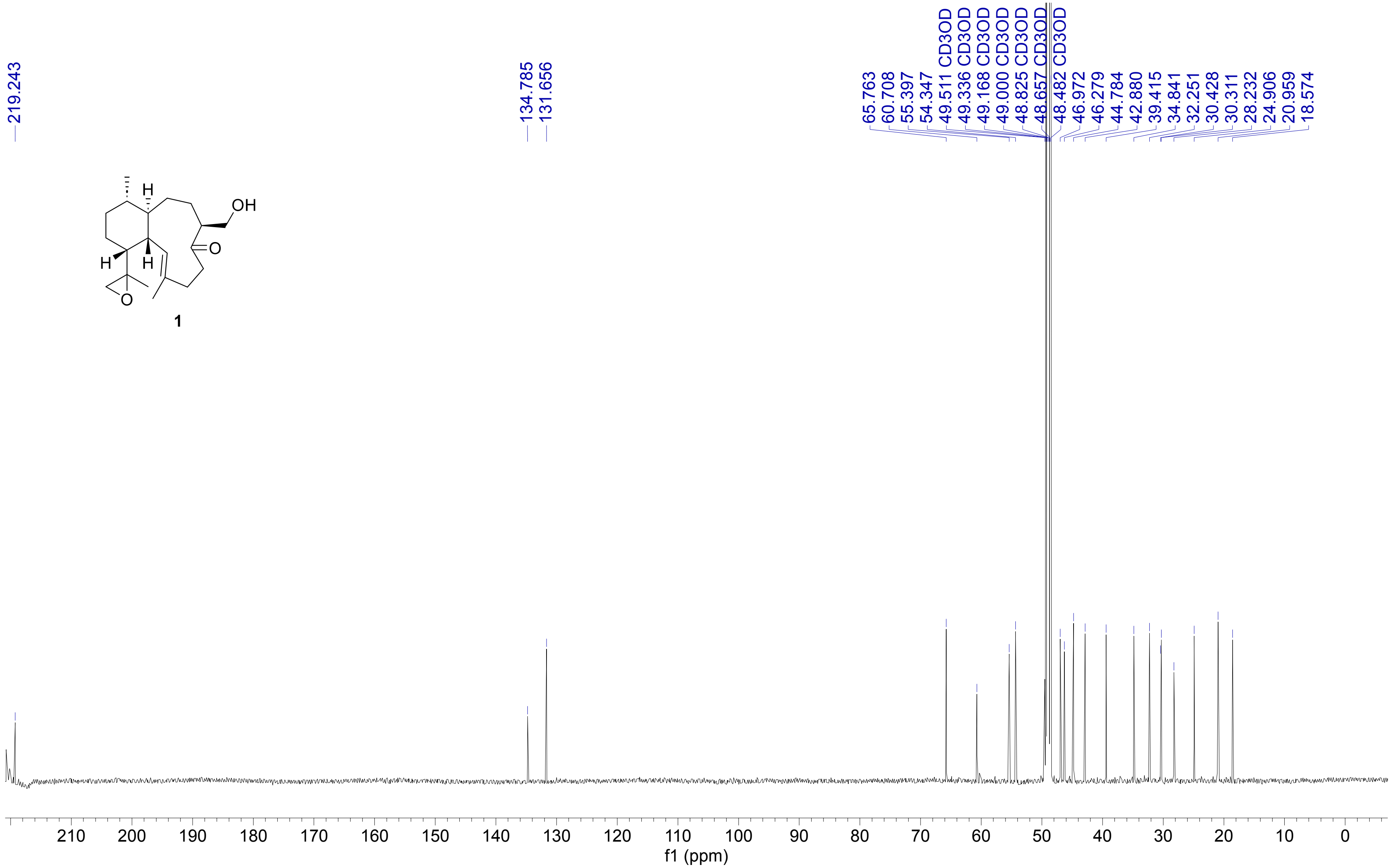

Figure S12. $^{13}C\{^{1}H\}$ NMR spectrum (125.64 MHz, 303K) of euthailol A (**1**) in CD_3OD.

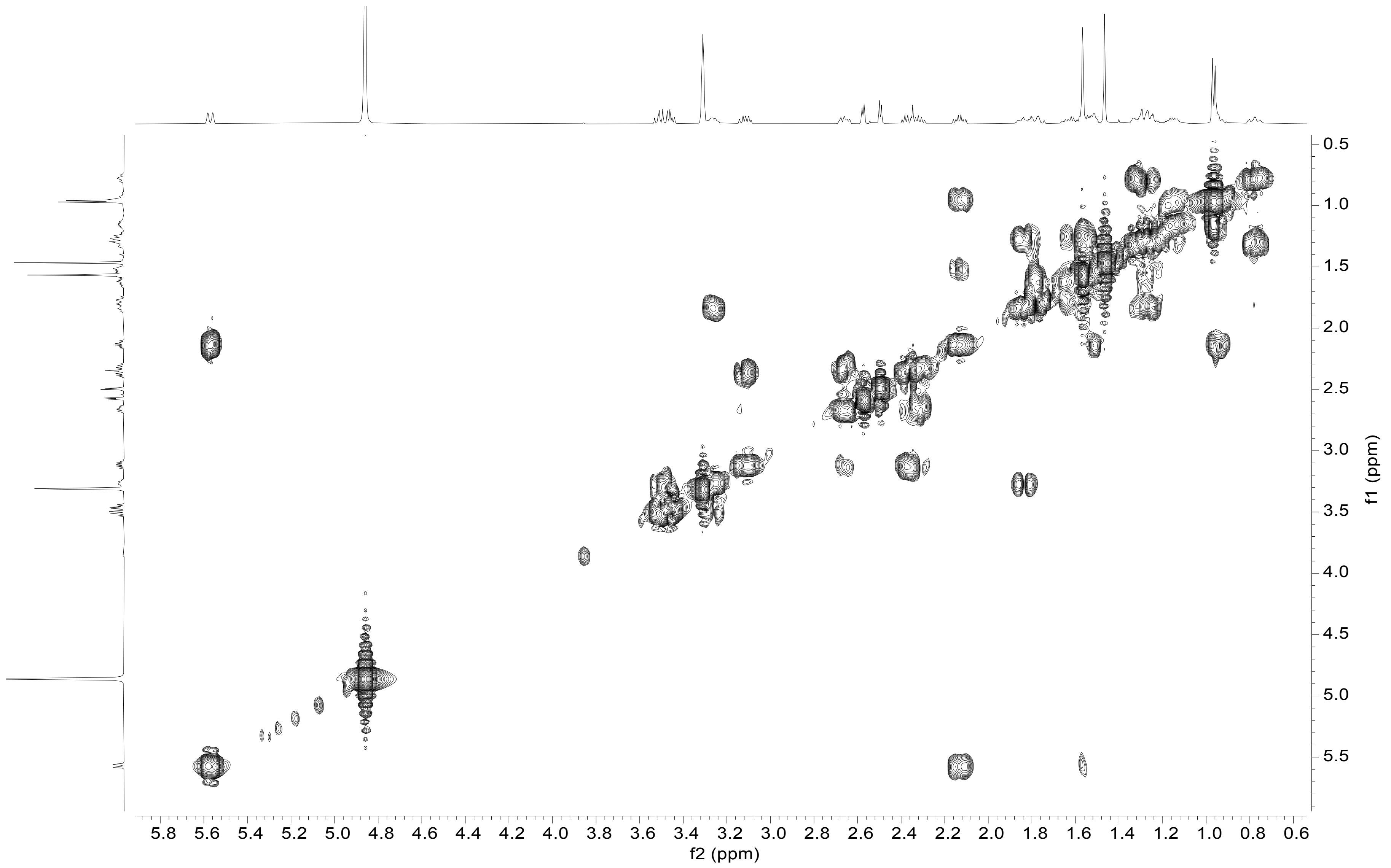

Figure S13. ^{1}H–^{1}H COSY spectrum (303K) of euthailol A (**1**) in CD_3OD.

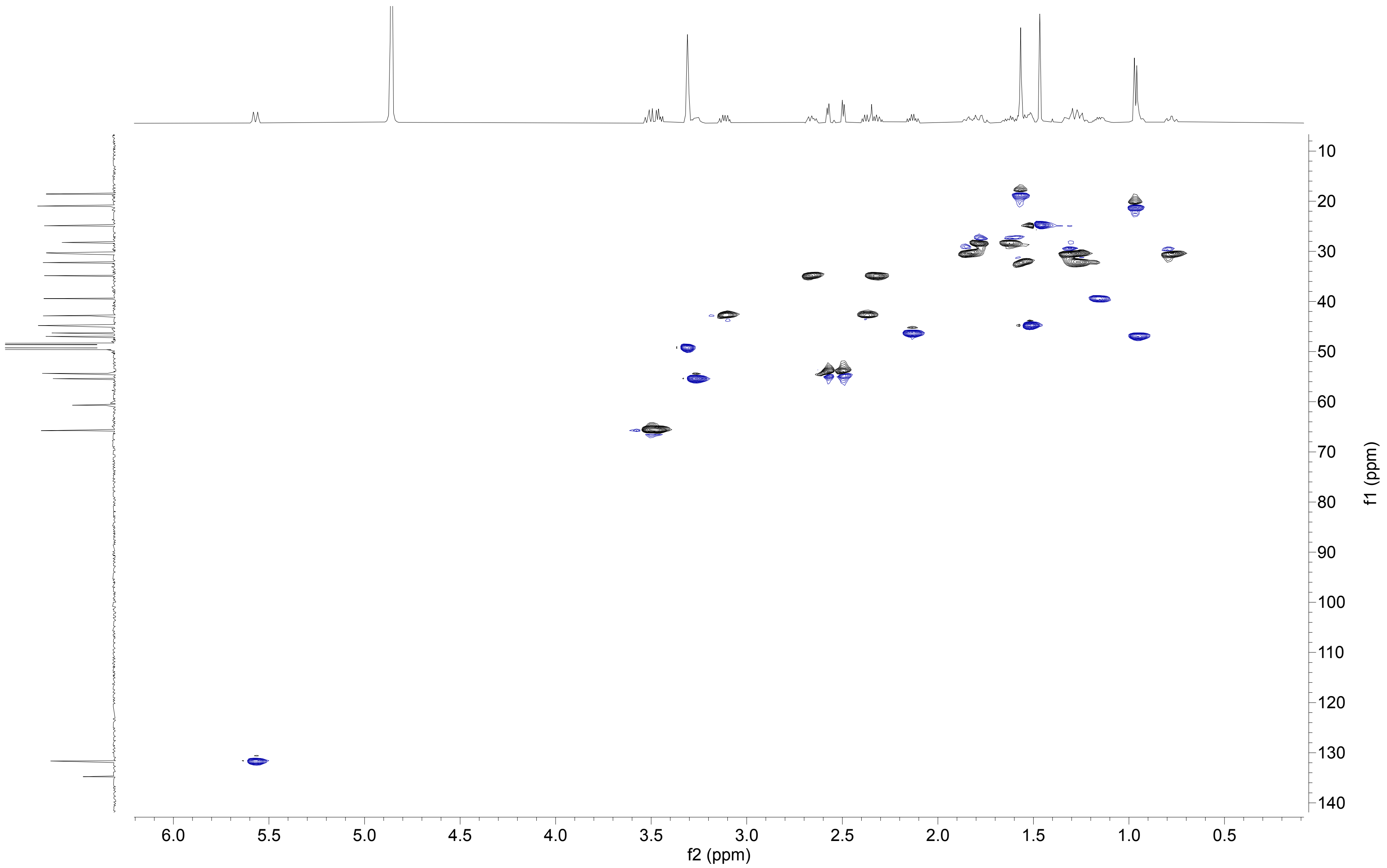

Figure S14. HSQC spectrum (303K) of euthailol A (**1**) in CD_3OD.

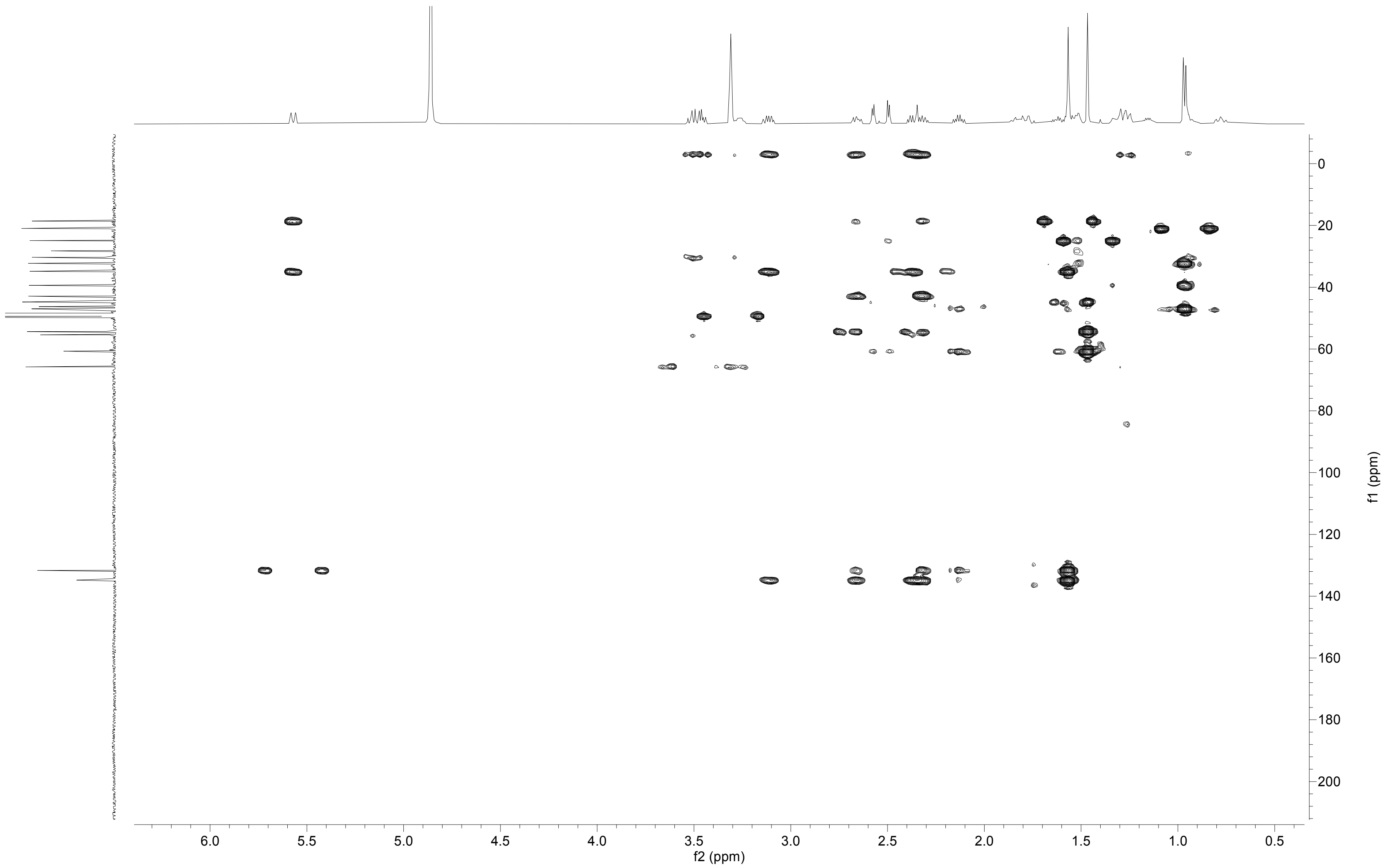

Figure S15. HMBC spectrum (303K) of euthailol A (**1**) in CD_3OD.

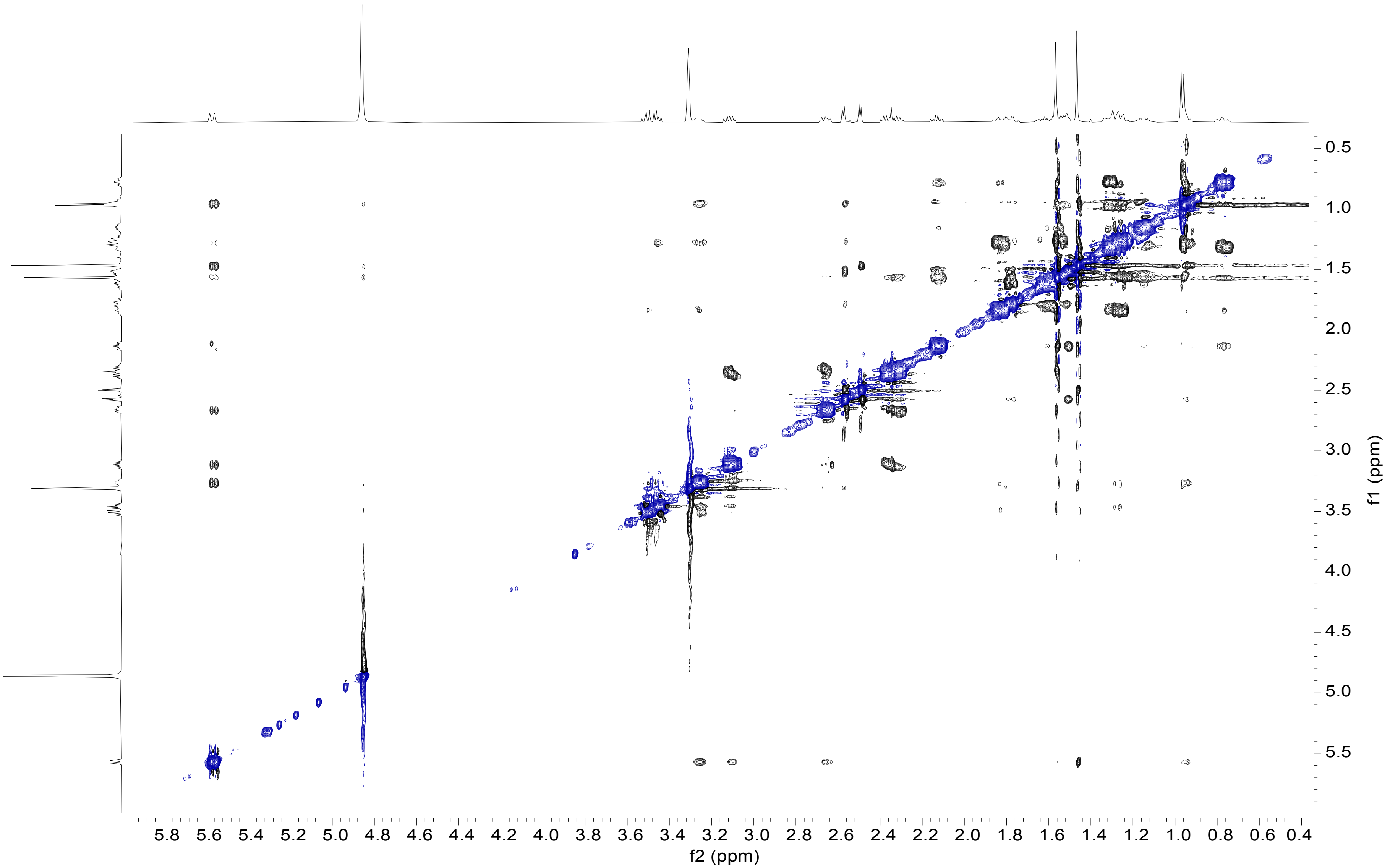

Figure S16. NOESY spectrum (303K) of euthailol A (**1**) in CD_3OD.

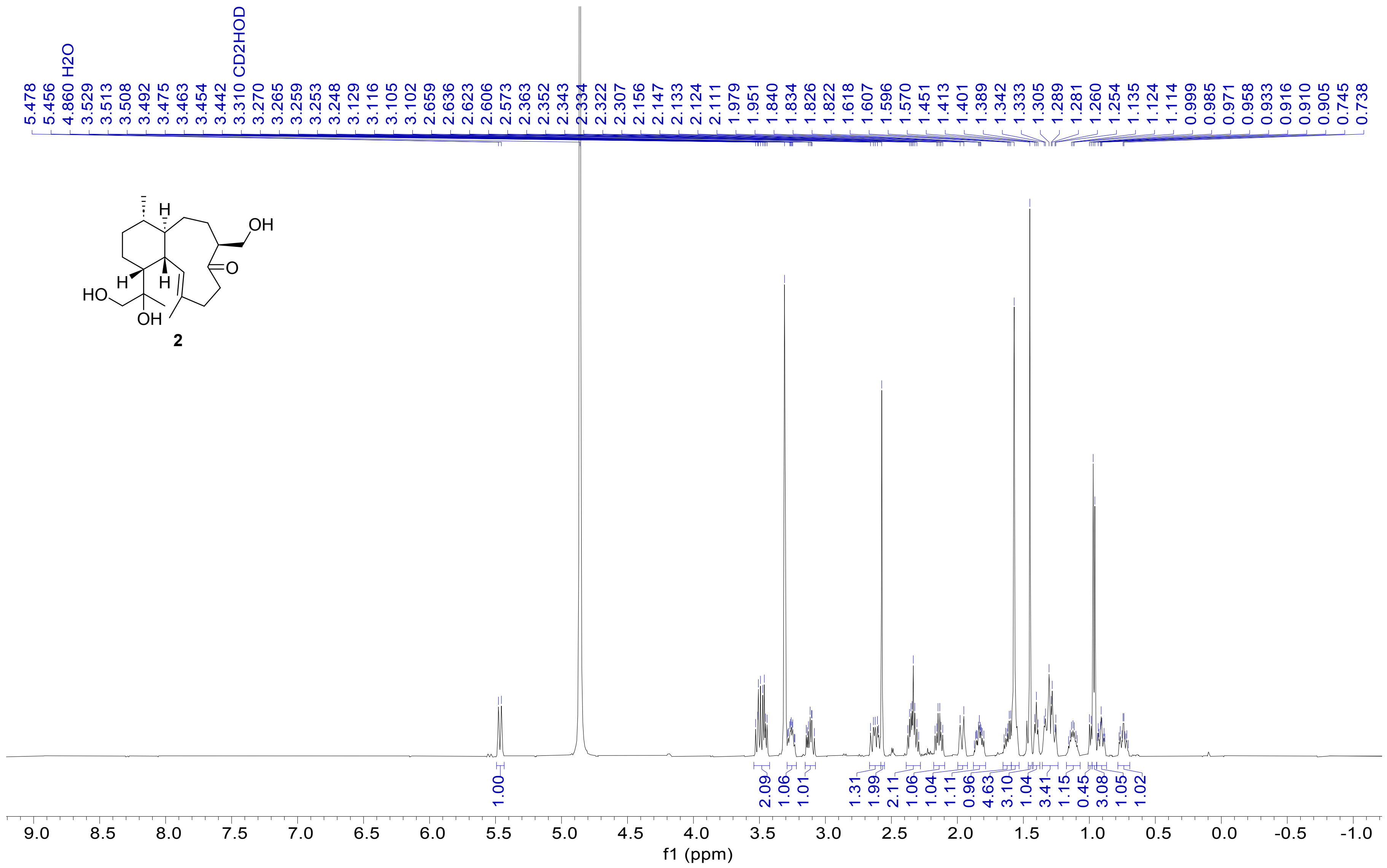

Figure S17. ^{1}H NMR spectrum (499.63 MHz, 303K) of euthailol B (**2**) in CD_3OD.

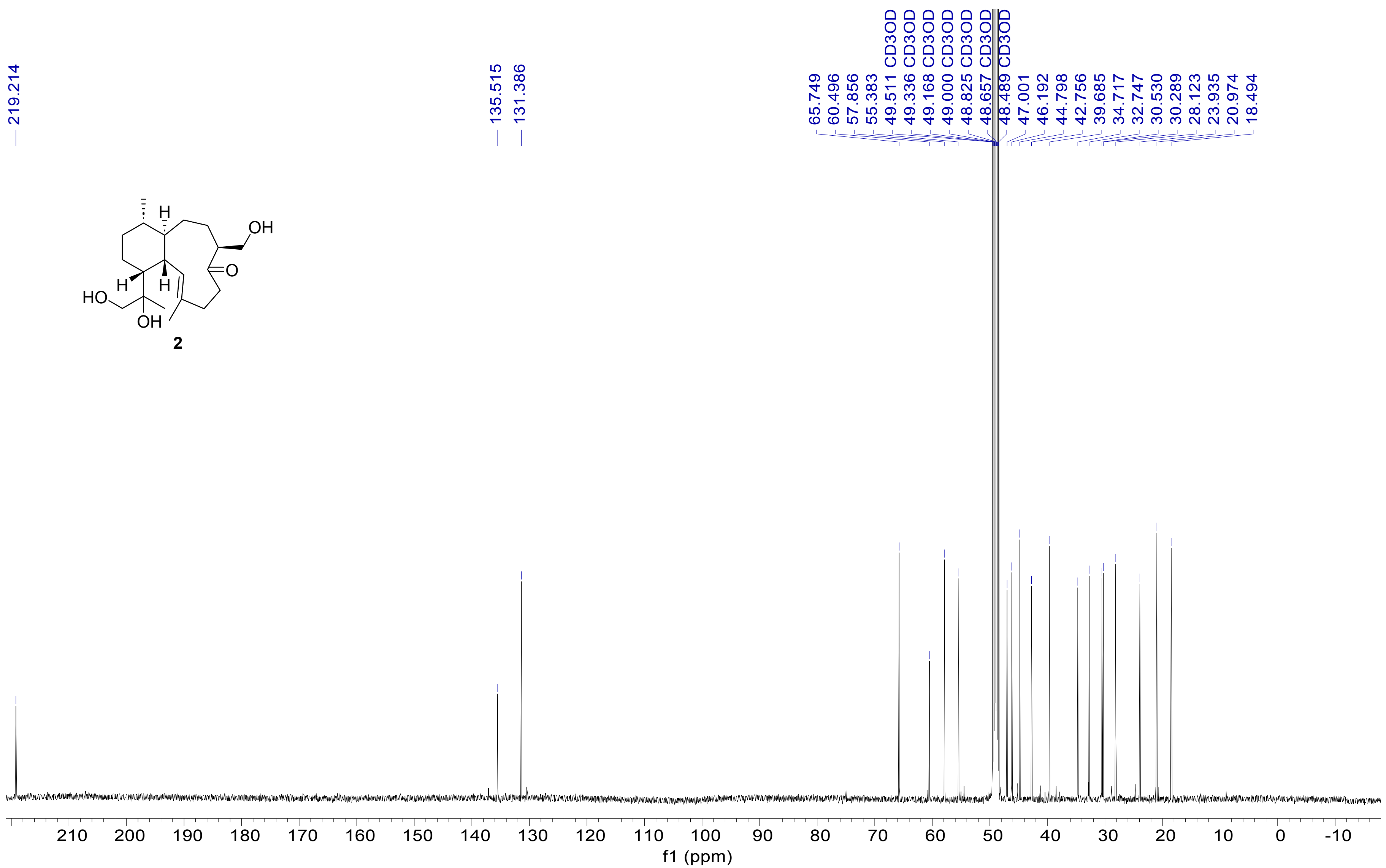

Figure S18. $^{13}C\{^{1}H\}$ NMR spectrum (125.64 MHz, 303K) of euthailol B (**2**) in CD_3OD.

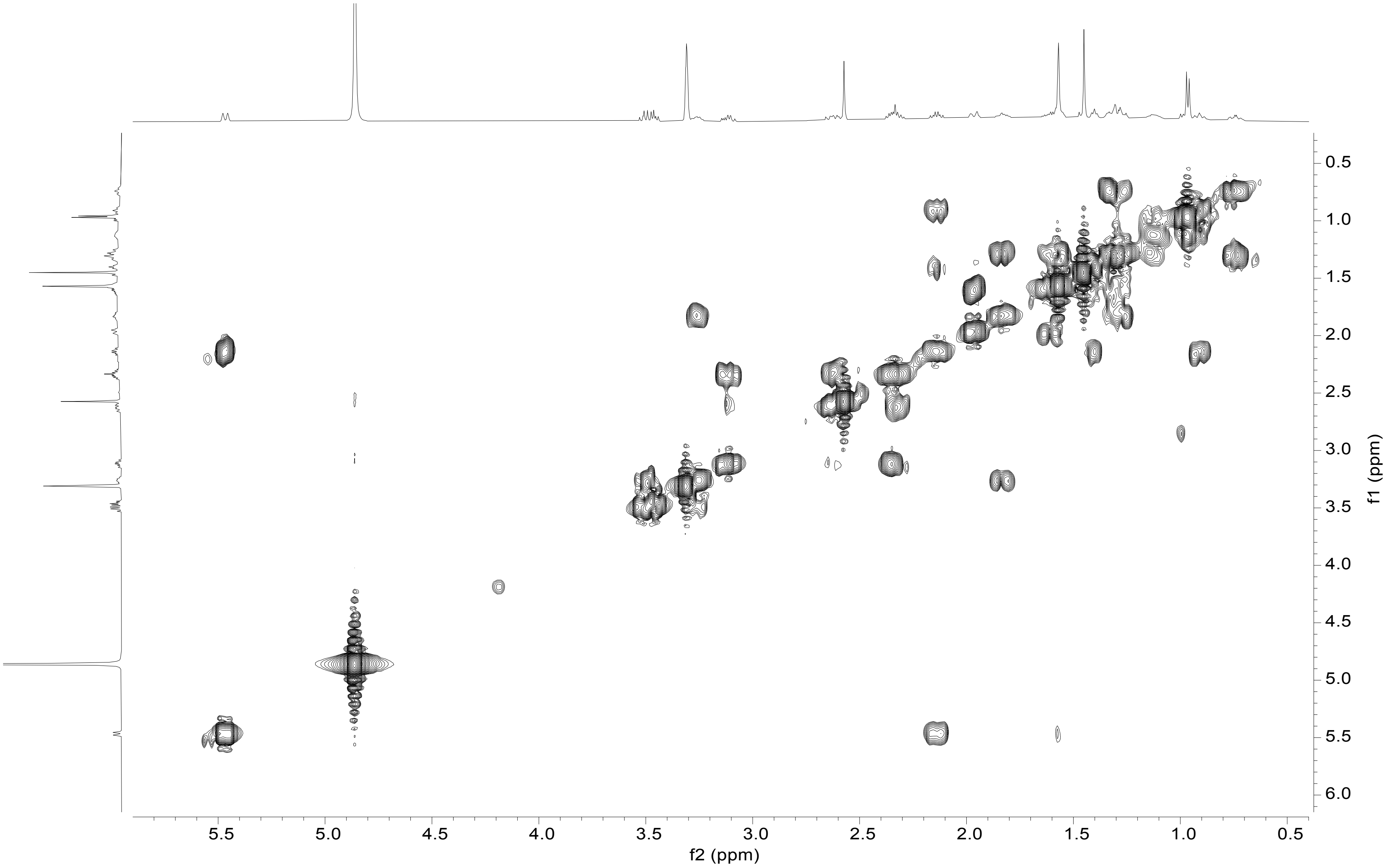

Figure S19. ^1H–^1H COSY spectrum (303K) of euthailol B (**2**) in CD_3OD.

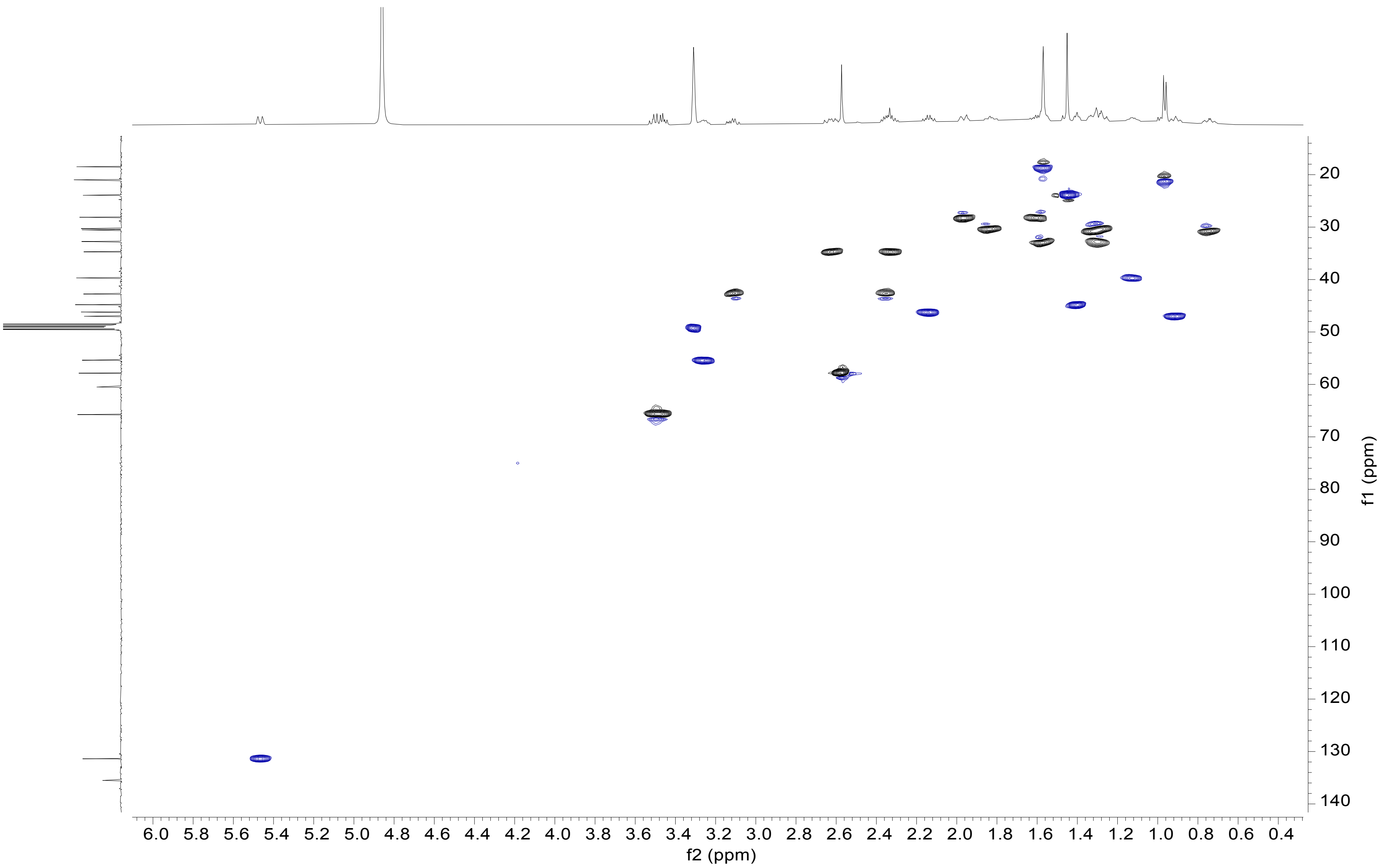

Figure S20. HSQC spectrum (303K) of euthailol B (**2**) in CD_3OD.

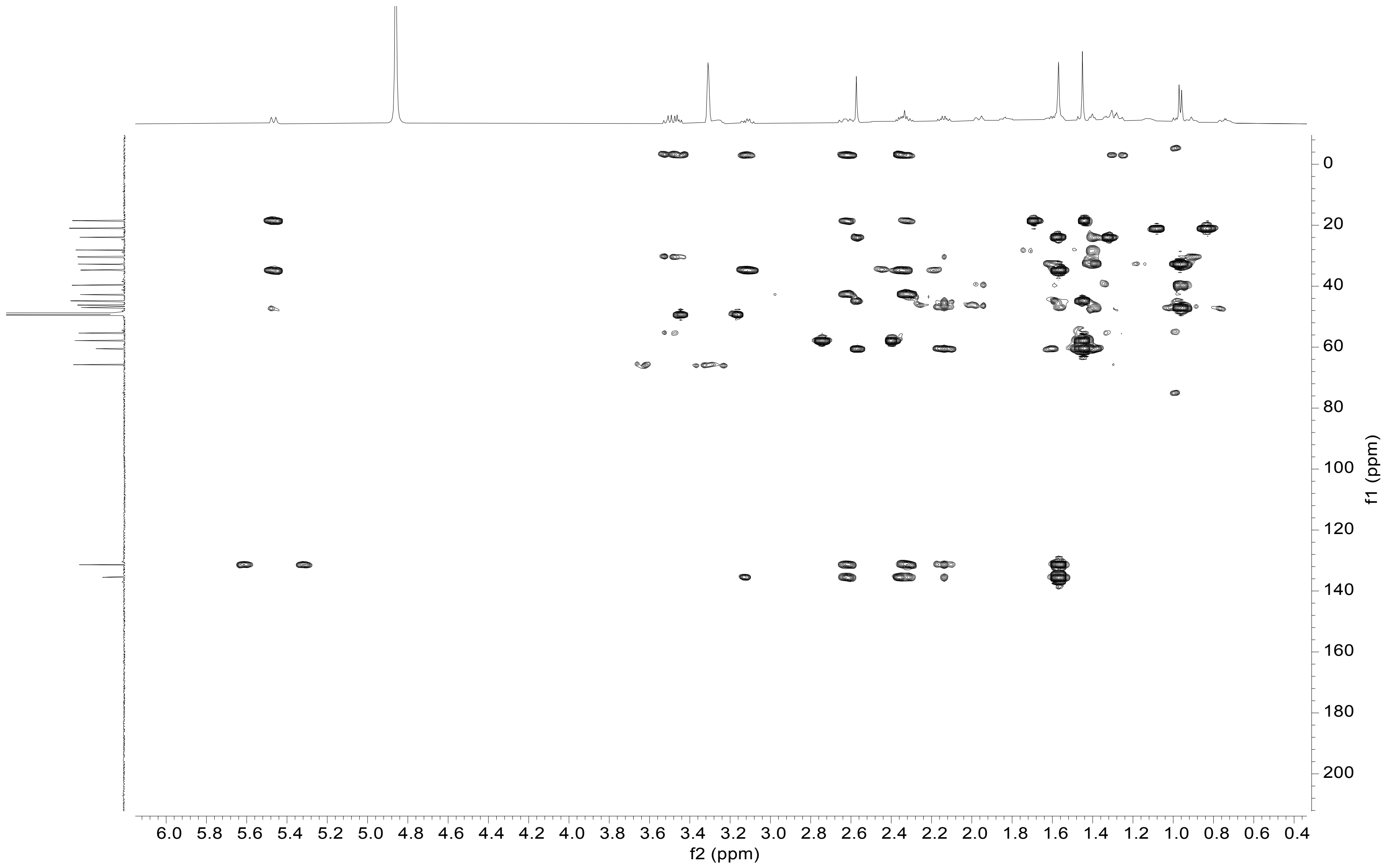

Figure S21. HMBC spectrum (303K) of euthailol B (**2**) in CD_3OD.

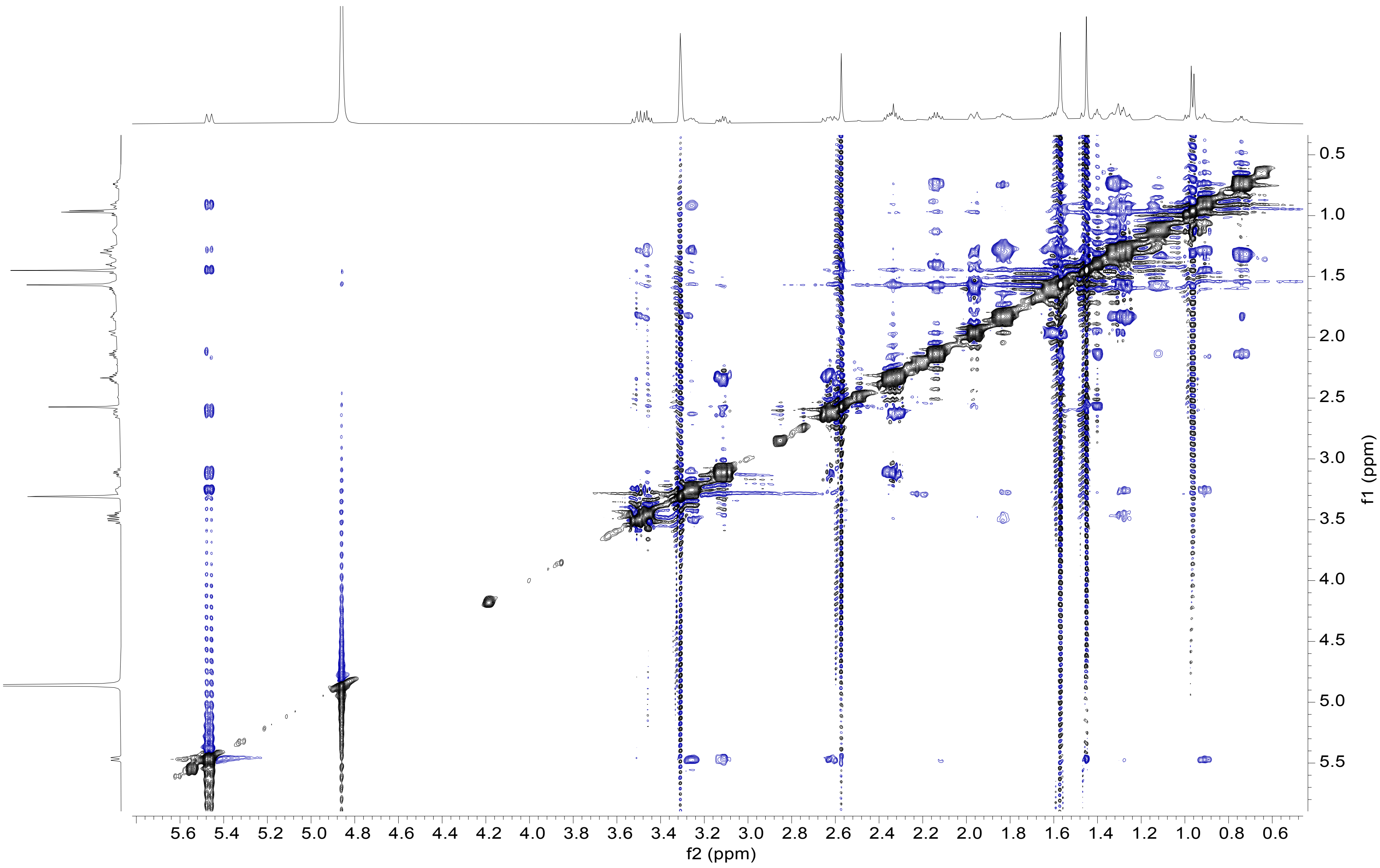

Figure S22. NOESY spectrum (303K) of euthailol B (**2**) in CD_3OD.

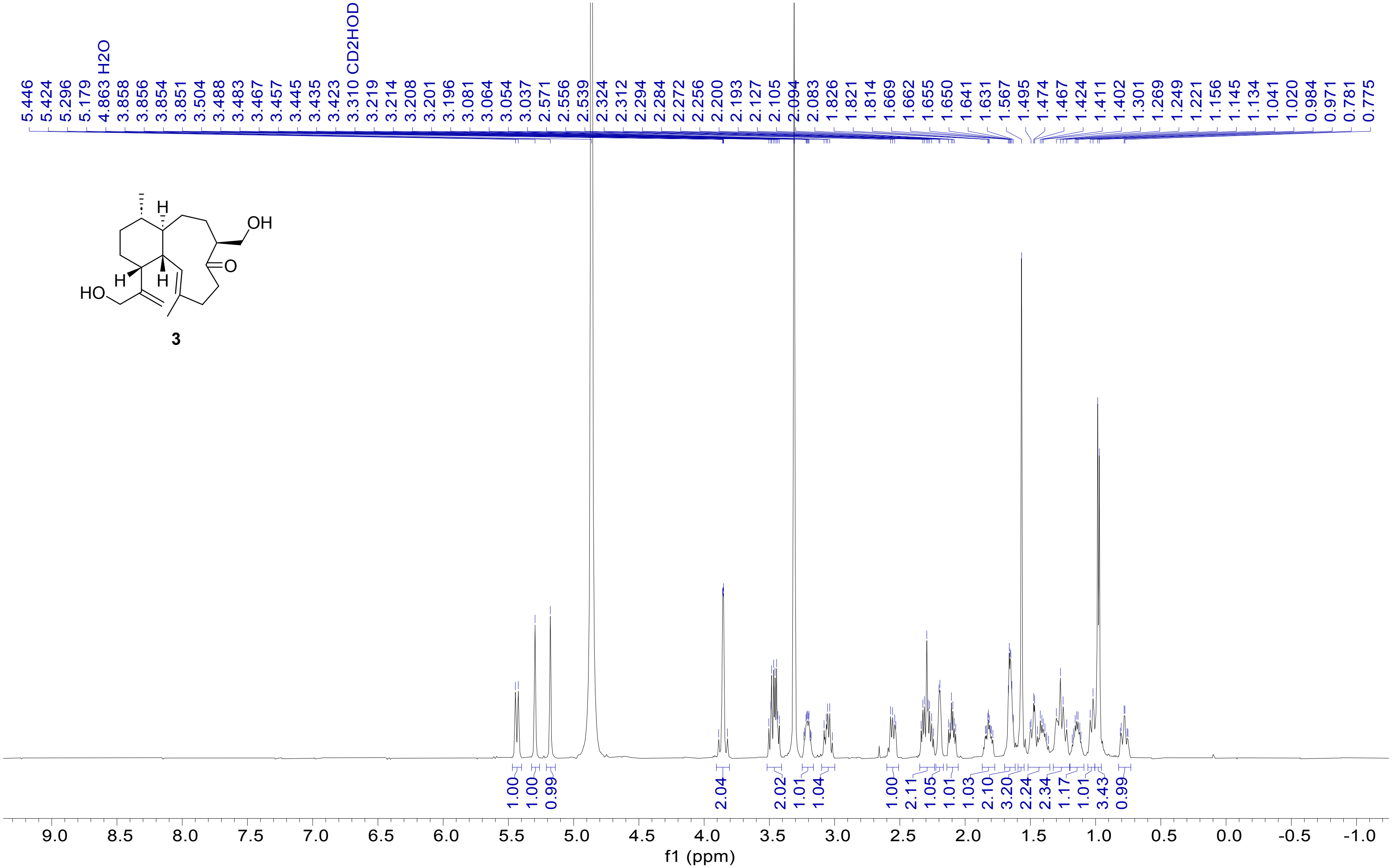

Figure S23. ^{1}H NMR spectrum (499.63 MHz, 303K) of euthailol C (**3**) in CD_3OD.

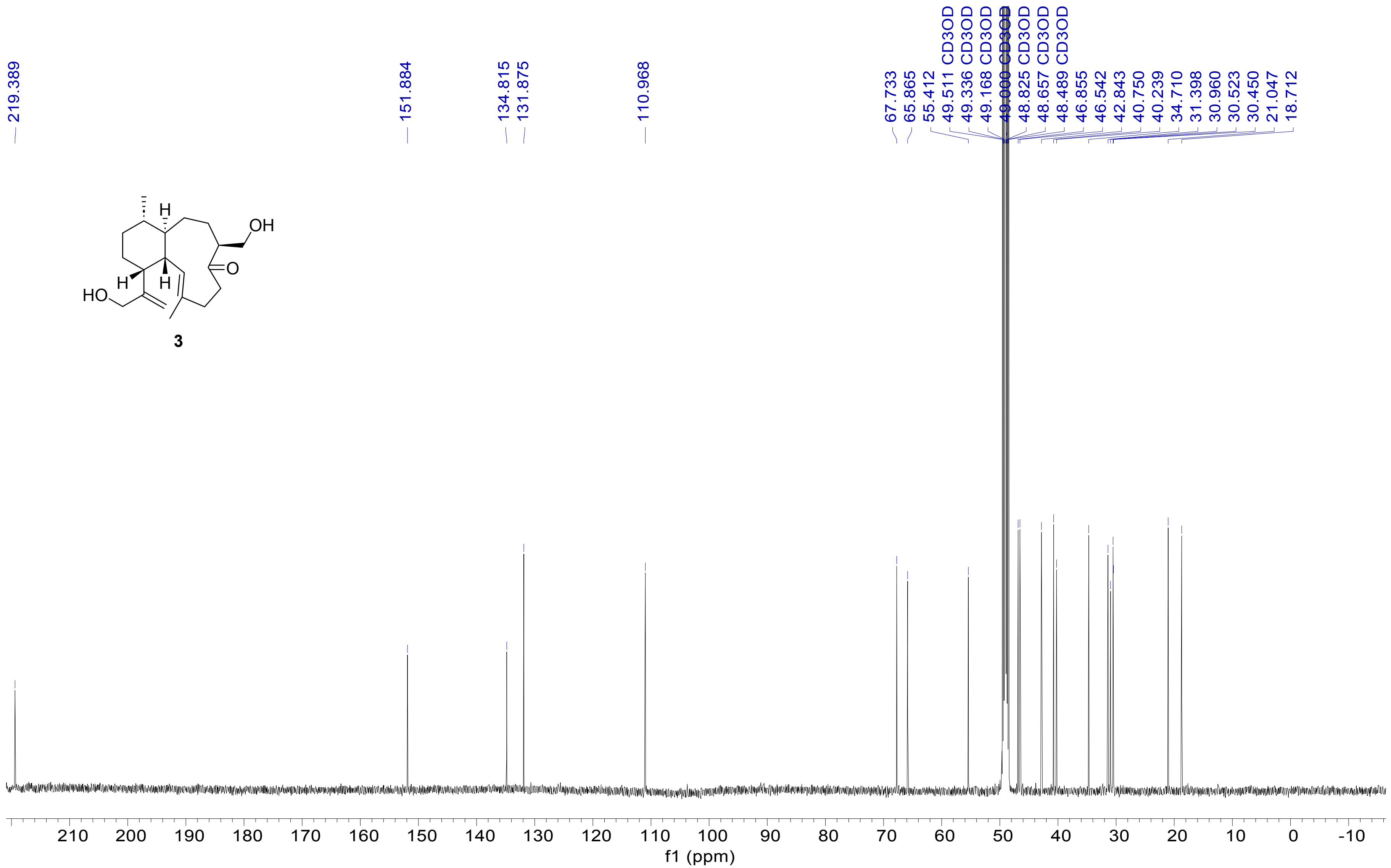

Figure S24. $^{13}C\{^{1}H\}$ NMR spectrum (125.64 MHz, 303K) of euthailol C (**3**) in CD_3OD.

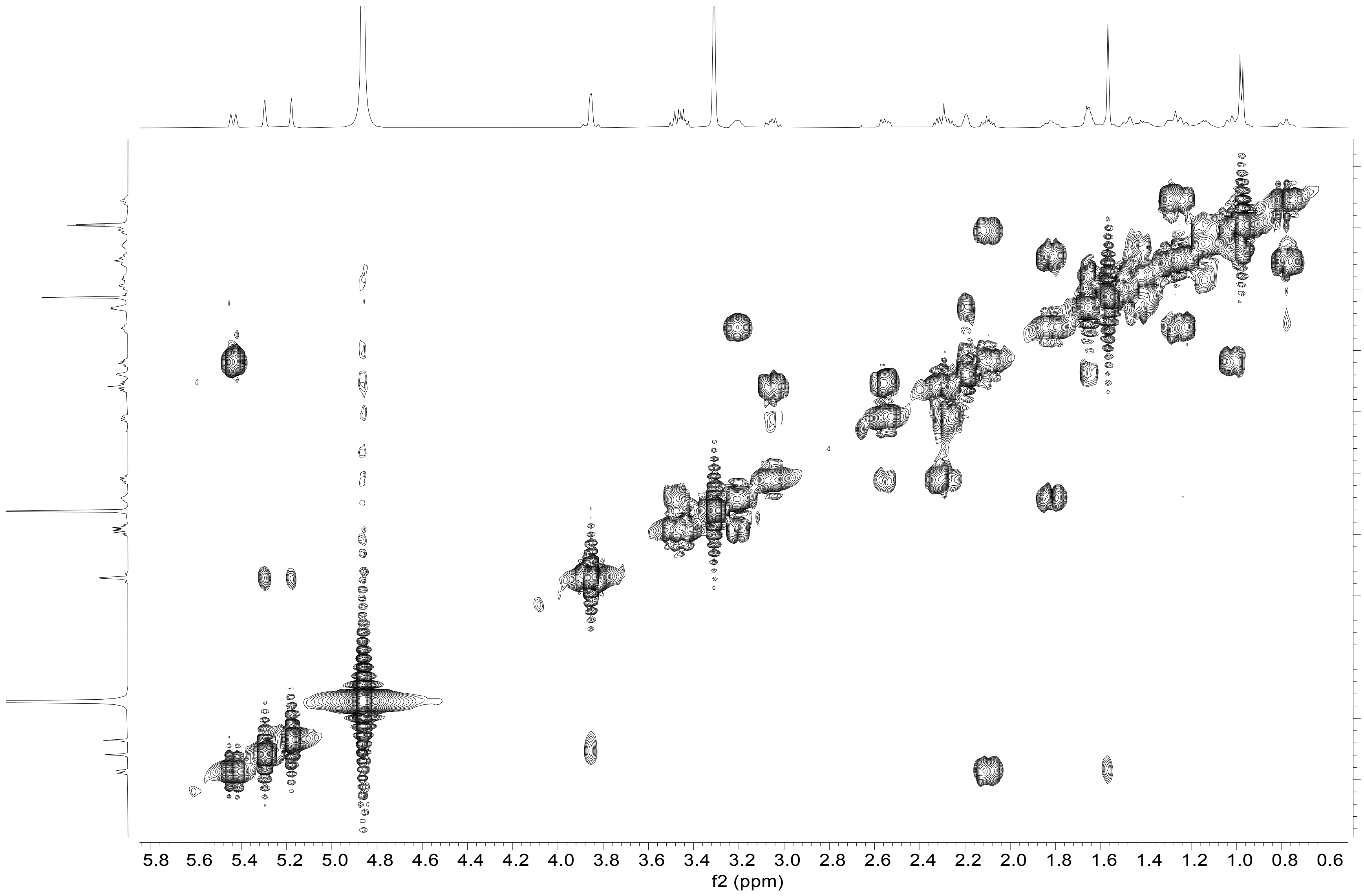

Figure S25. ^{1}H–^{1}H COSY spectrum (303K) of euthailol C (**3**) in CD_3OD.

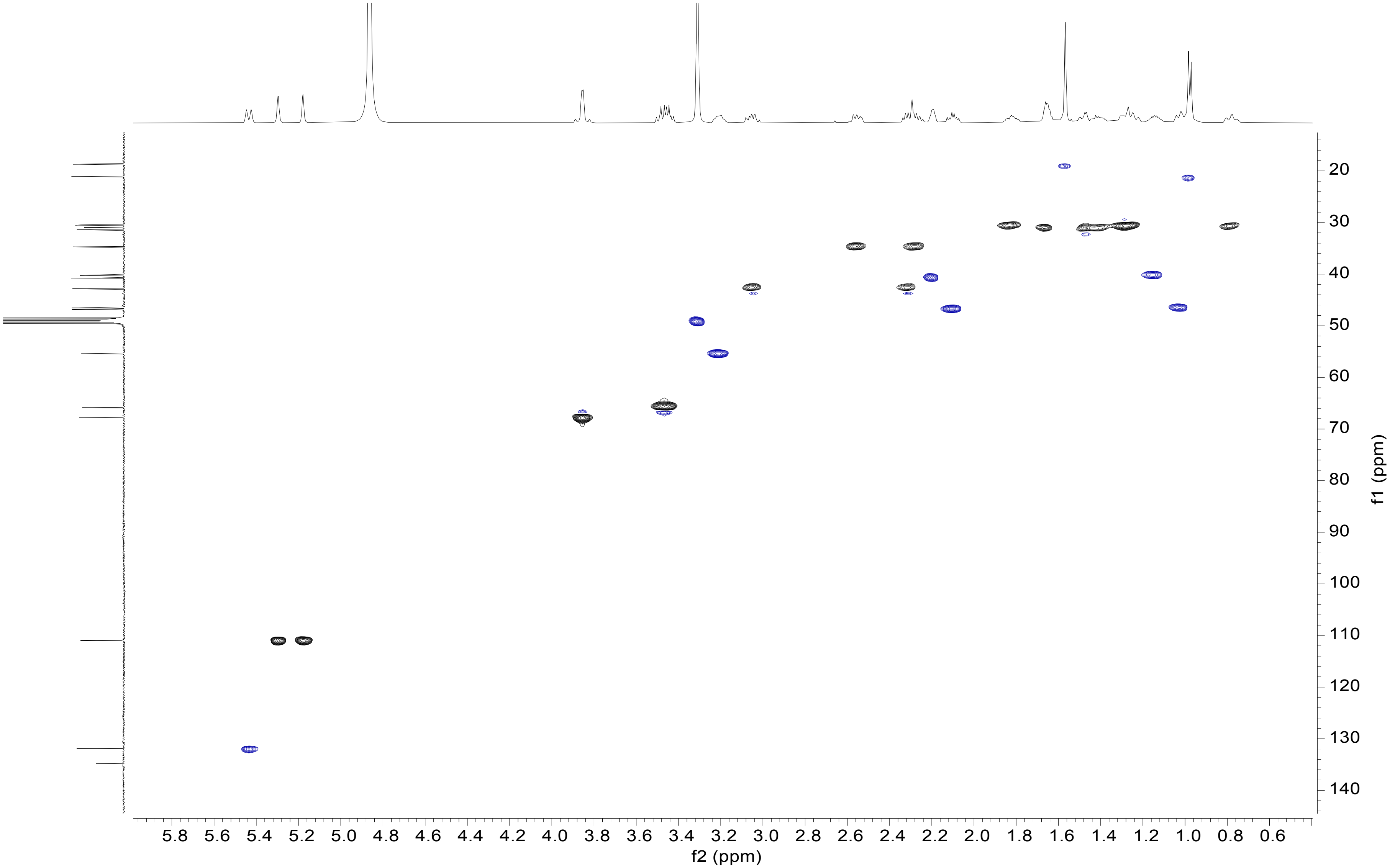

Figure S26. HSQC spectrum (303K) of euthailol C (**3**) in CD_3OD.

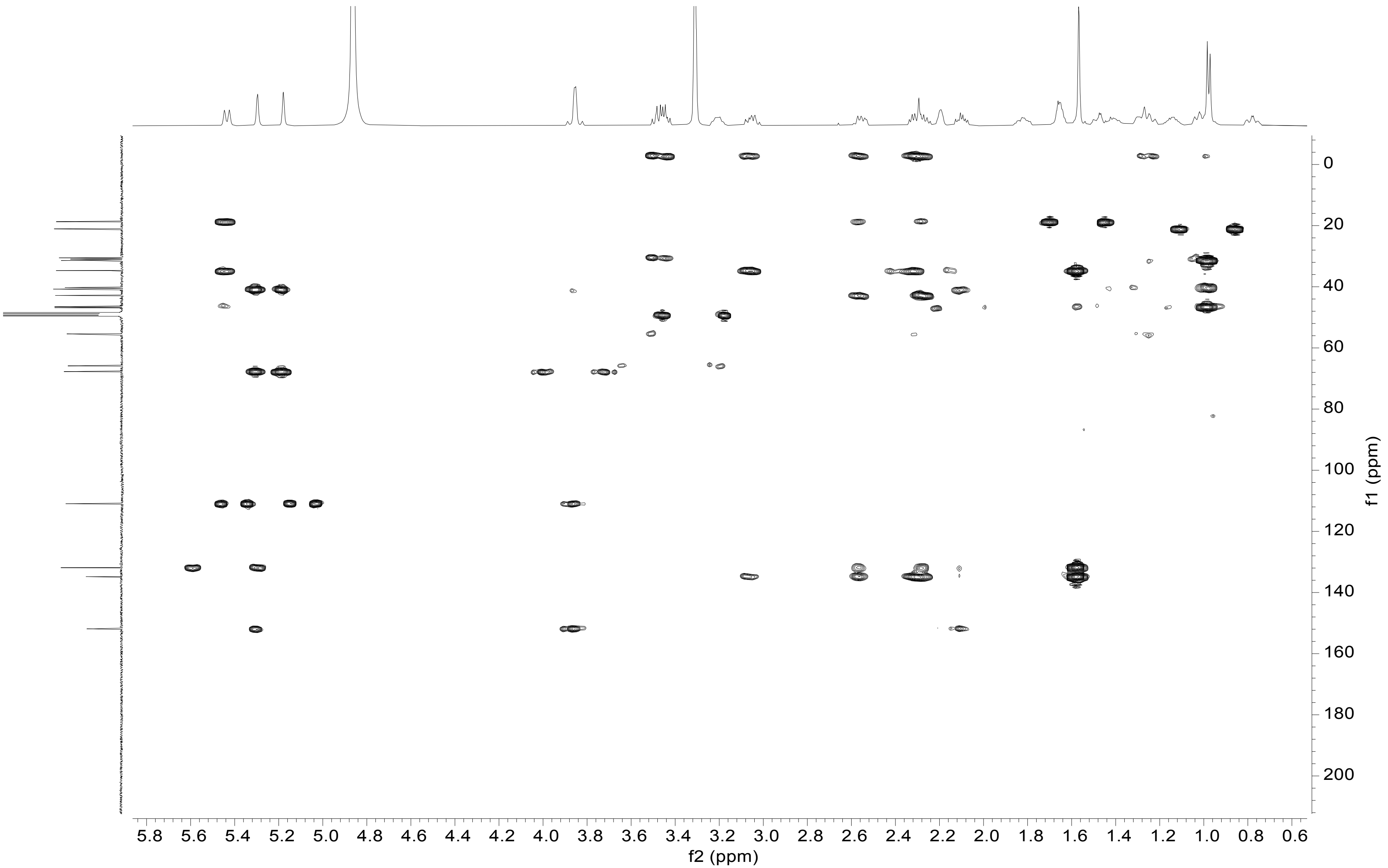

Figure S27. HMBC spectrum (303K) of euthailol C (**3**) in CD_3OD.

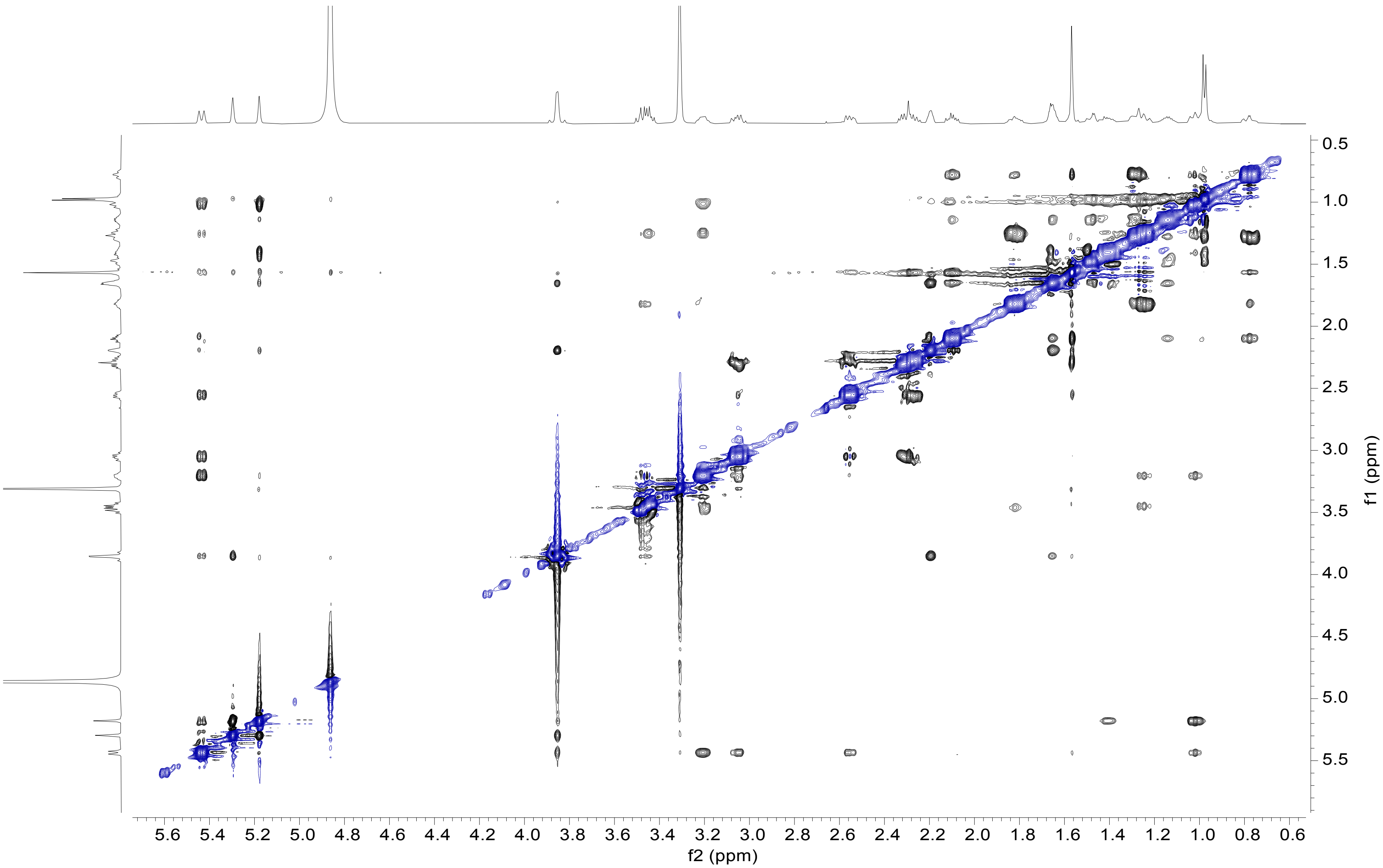

Figure S28. NOESY spectrum (303K) of euthailol C (**3**) in CD_3OD.

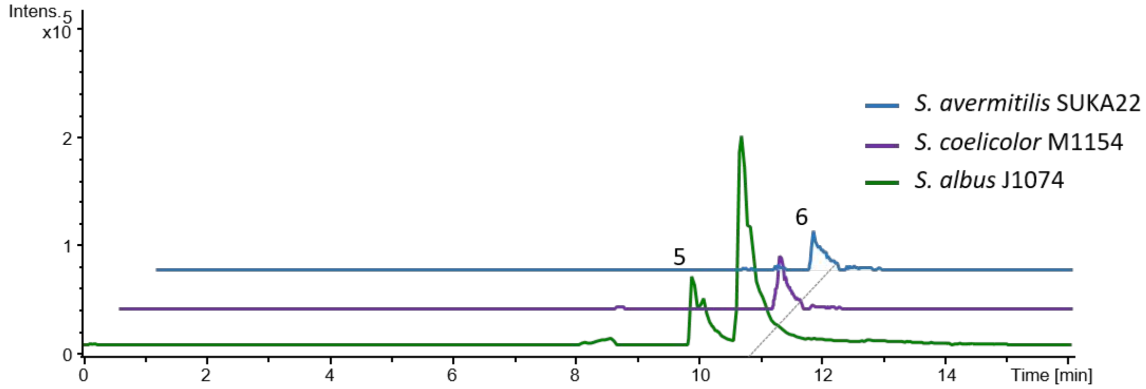

Fig S29: Extracted ion chromatogram of euthailol E (**5**) and F (**6**) at *m/z* 305.2474 $[M+H]^+$ of culture extracts of *S. avermitilis* SUKA22, *S. coelicolor* M1154, and *S. albus* J1074, respectively, that harbor the *eut* BGC.

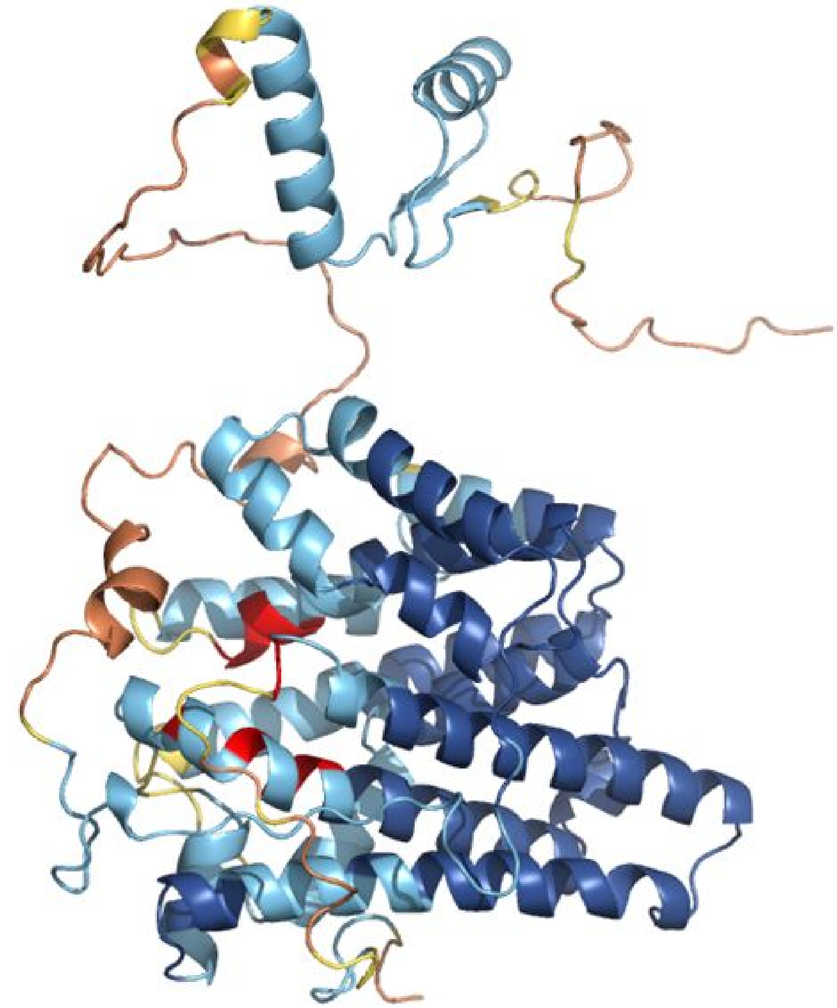

Figure S30: Protein model of the di-domain reductase-cyclase EutB created with AlphaFold 2. Regions of very low confidence (pLDDT < 50) are shown in orange, regions of low confidence (70 > pLDDT > 50) are shown in yellow, regions of high confidence (90 > pLDDT > 70) are shown in light blue and regions of very high confidence (pLDDT> 90) are shown in dark blue. The conserved motifs of class I terpene cyclases (DDXXD motif, NSE triad, RY motif) are colored in red.

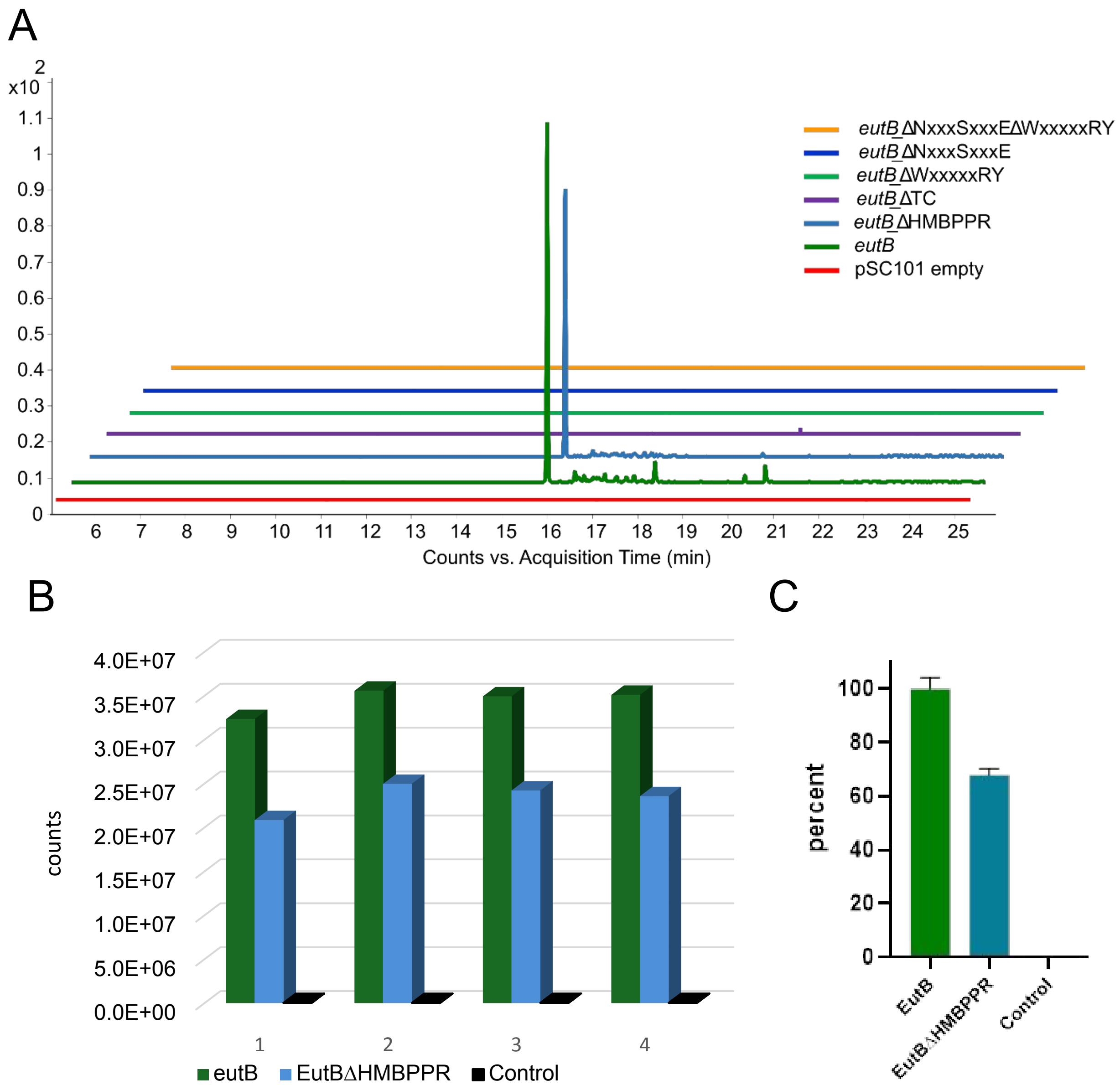

Figure S31. A. Extracted ion chromatogram of the *trans*-eunicellane scaffold (albireticulene) at *m/z* 272.20 $[M+H]^+$ from culture extracts of *E. coli* harboring either the full length di-domain reductase-cyclase (*eutB*) or truncated versions of *eutB* (knockouts of the reductase domain, the cyclase domain or catalytic motifs NxxxSxxxE and WxxxxxRY within the cyclase domain). *E. coli* harboring the empty plasmid served as a negative control. B. Bar graph comparing the absolute product yield of albireticulene from *E. coli* extracts harboring the full-length eutB and eutB in which the reductase domain had been knocked out. C. Bar graph comparing the relative product yield of albireticulene from *E. coli* extracts harboring the full-length eutB and eutB in which the reductase domain had been knocked out. The experiments have been conducted in quadruplicates.

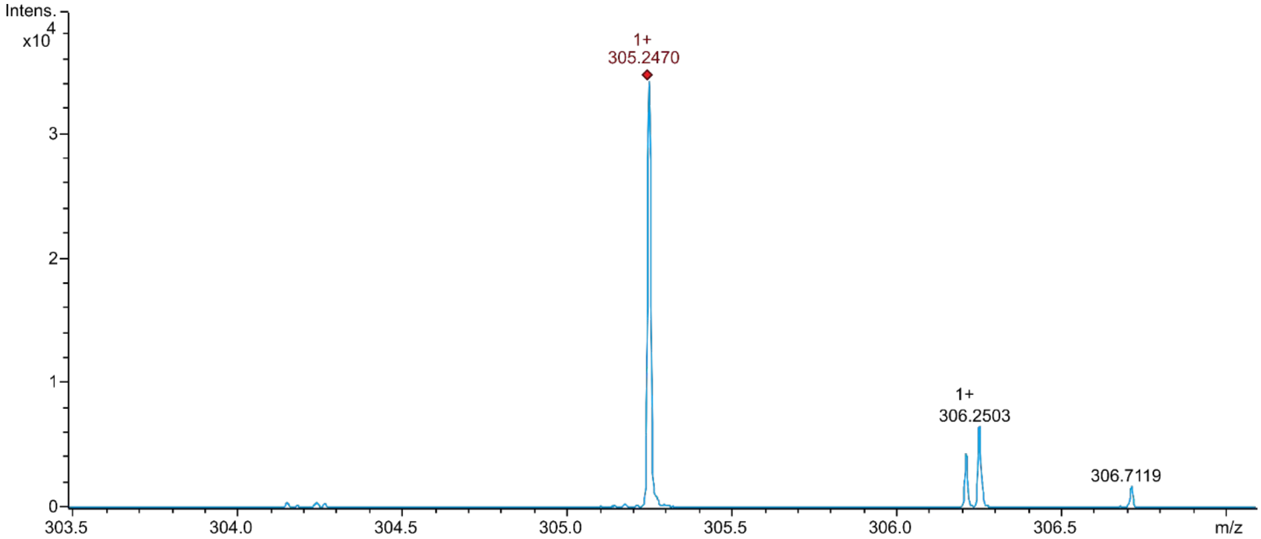

Fig S32: Isotopic pattern of euthailol D (**4**).

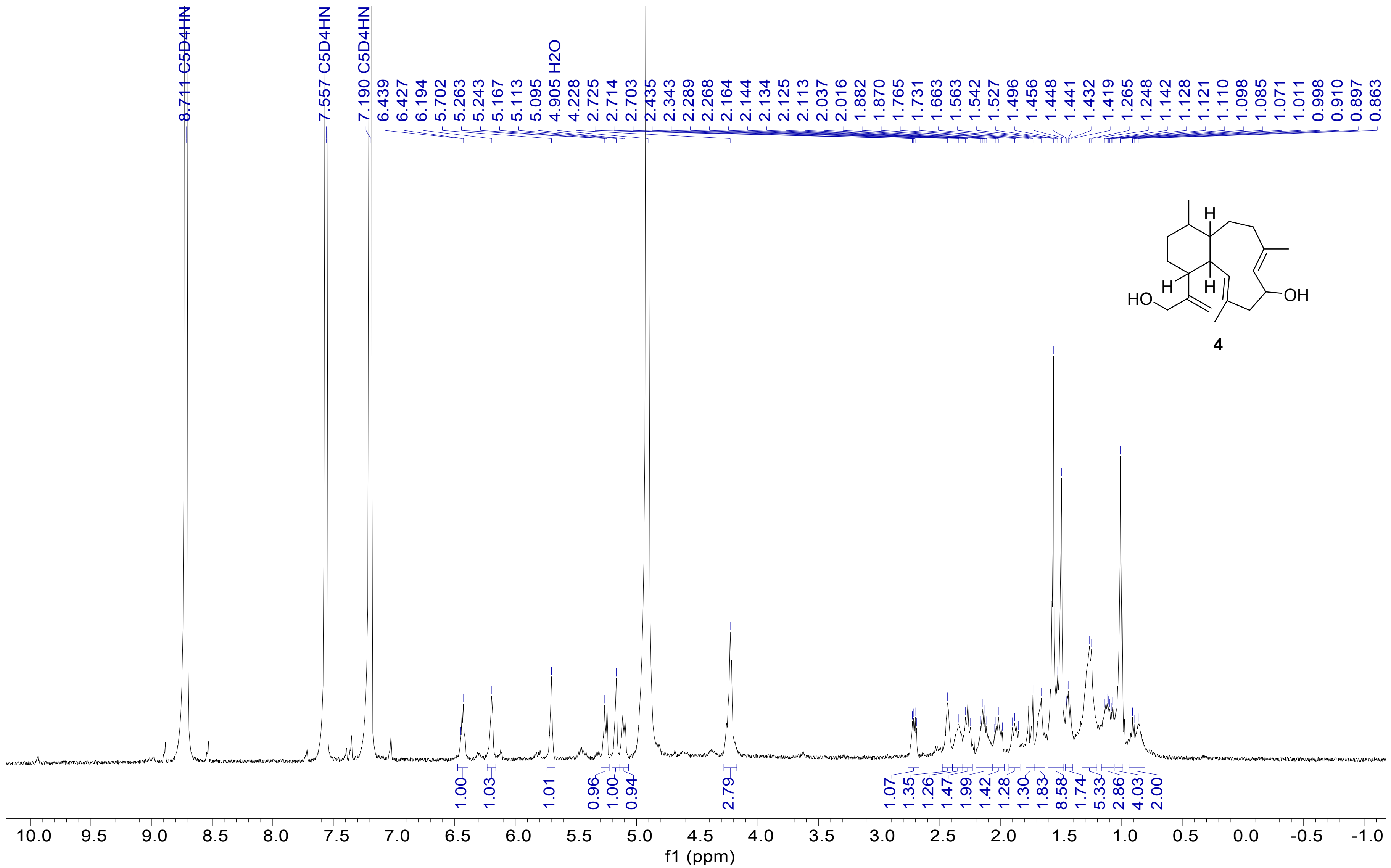

Figure S33. ^{1}H NMR spectrum (500.18 MHz, 298K) of euthailol D (**4**) in C_5D_5N.

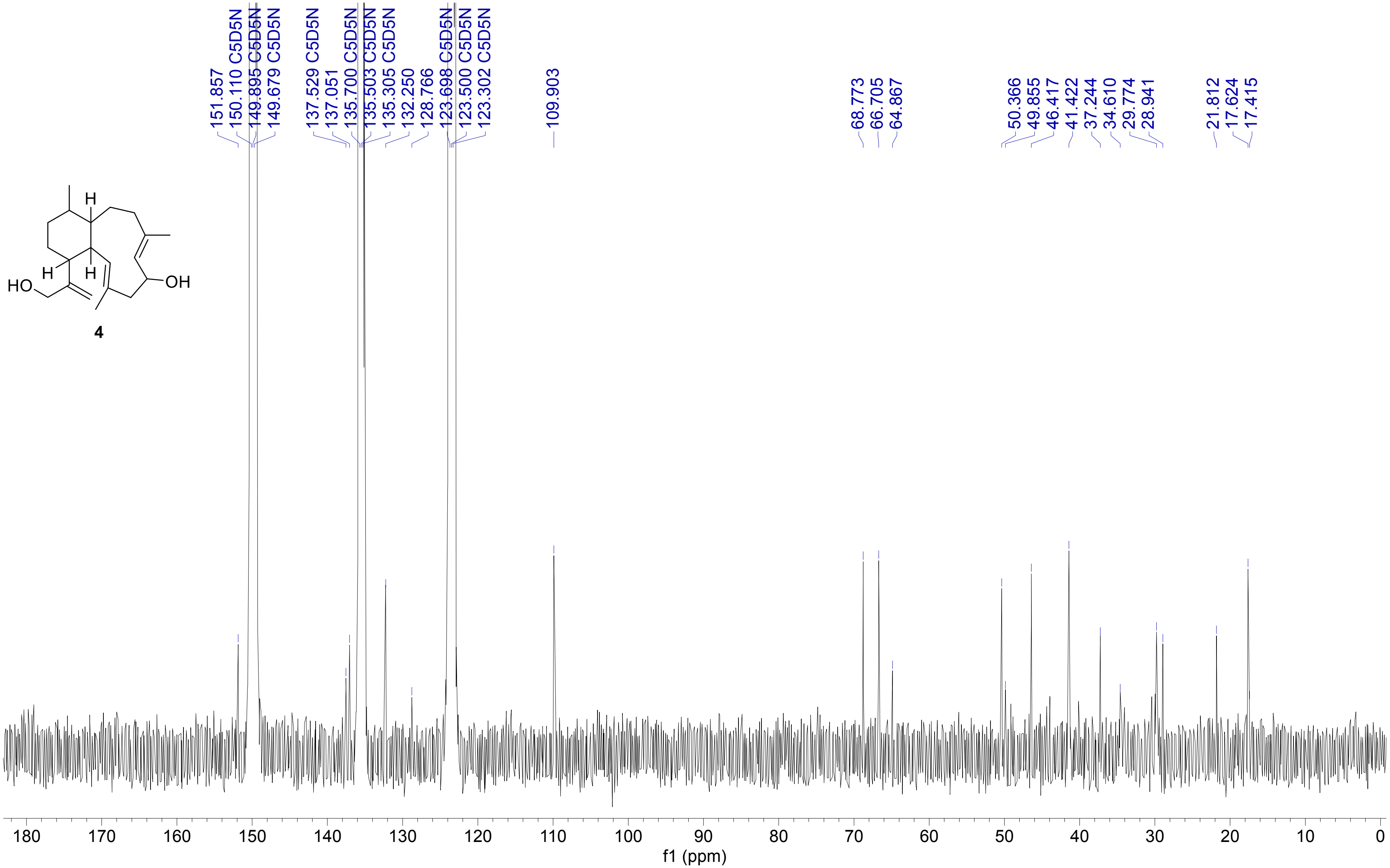

Figure S34. $^{13}C\{^{1}H\}$ NMR spectrum (125.78 MHz, 298K) of euthailol D (**4**) in C_5D_5N.

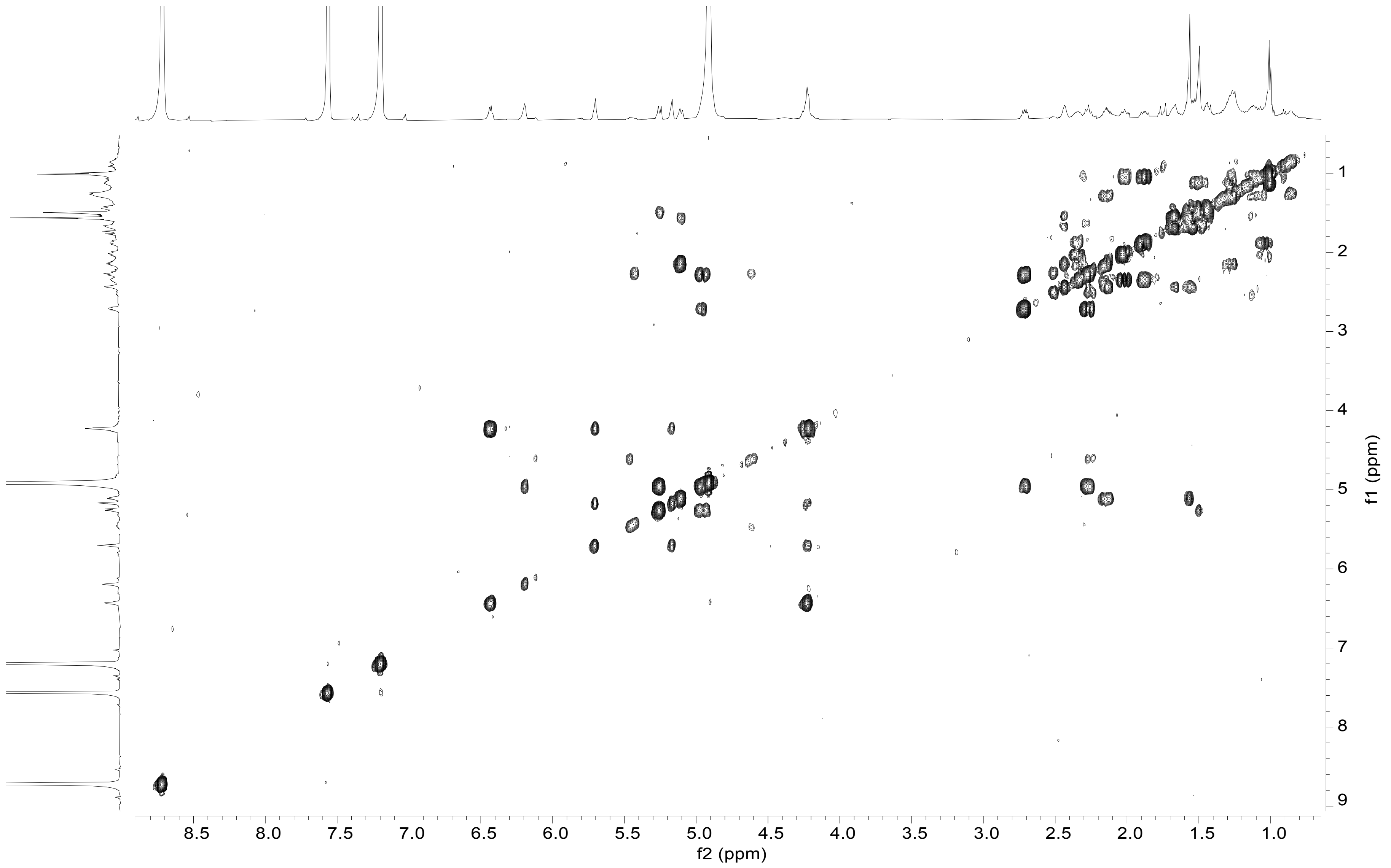

Figure S35. ^{1}H–^{1}H COSY spectrum (298K) of euthailol D (**4**) in C_5D_5N.

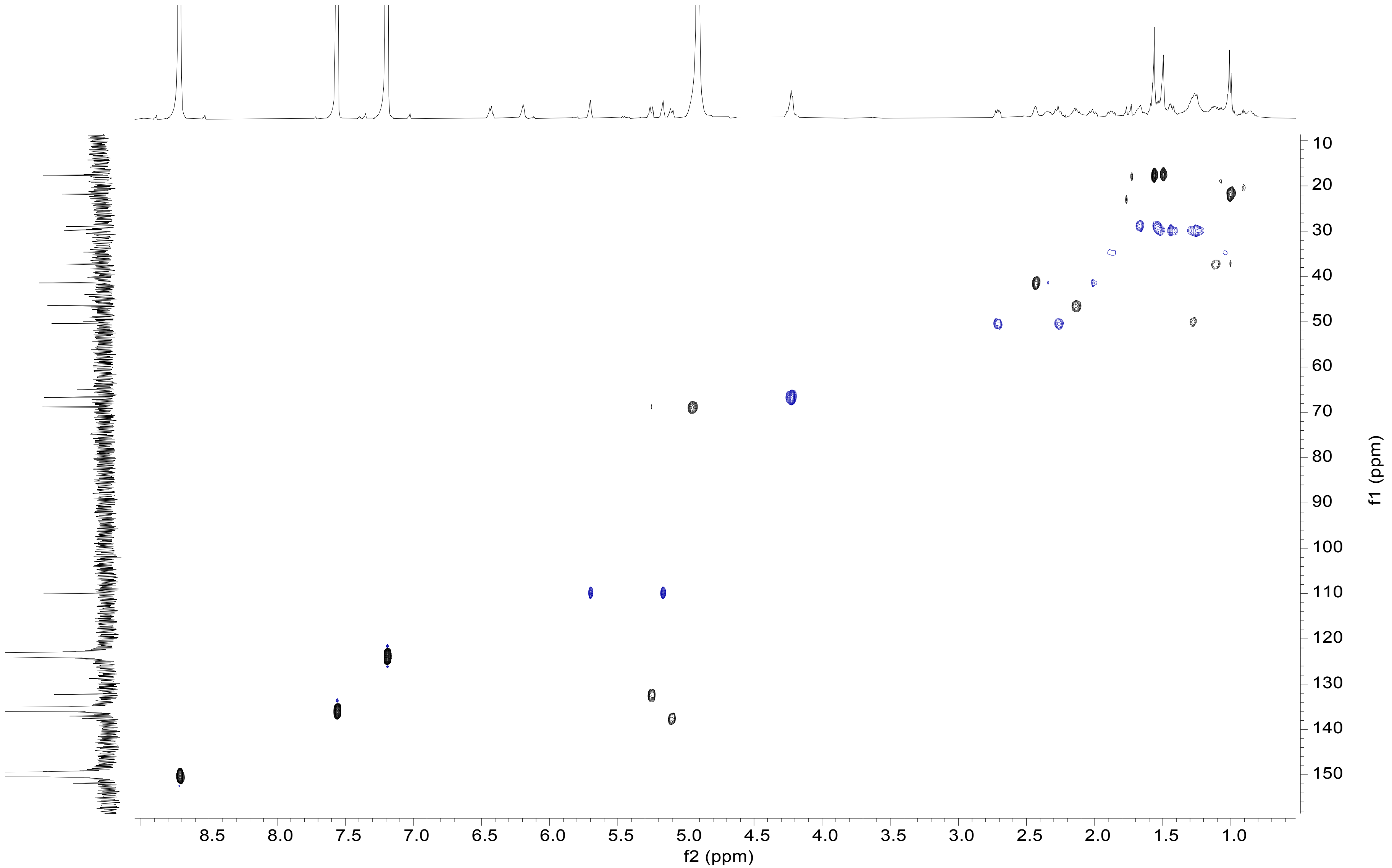

Figure S36. HSQC spectrum (298K) of euthailol D (**4**) in C_5D_5N.

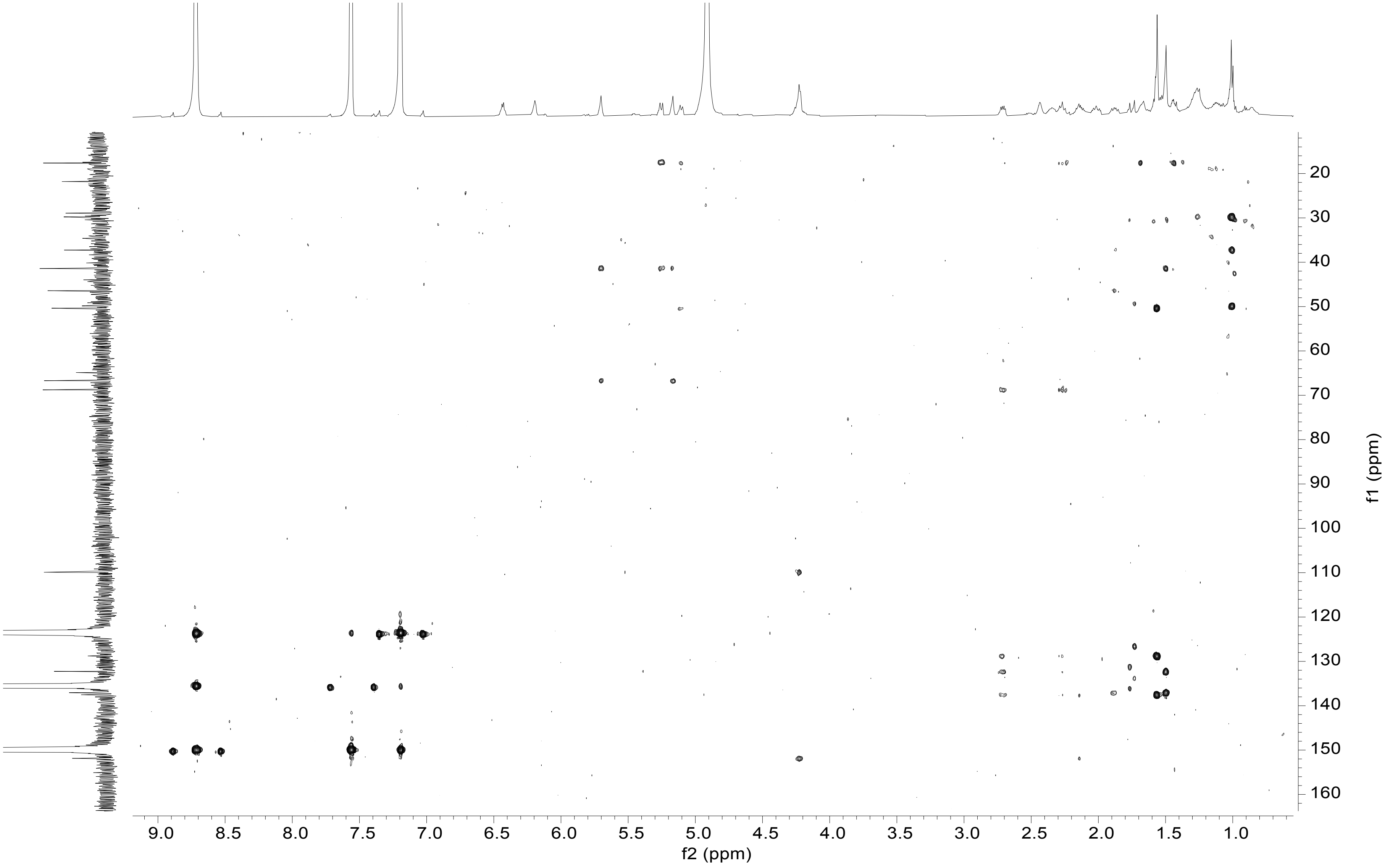

Figure S37. HMBC spectrum (298K) of euthailol D (**4**) in C_5D_5N.

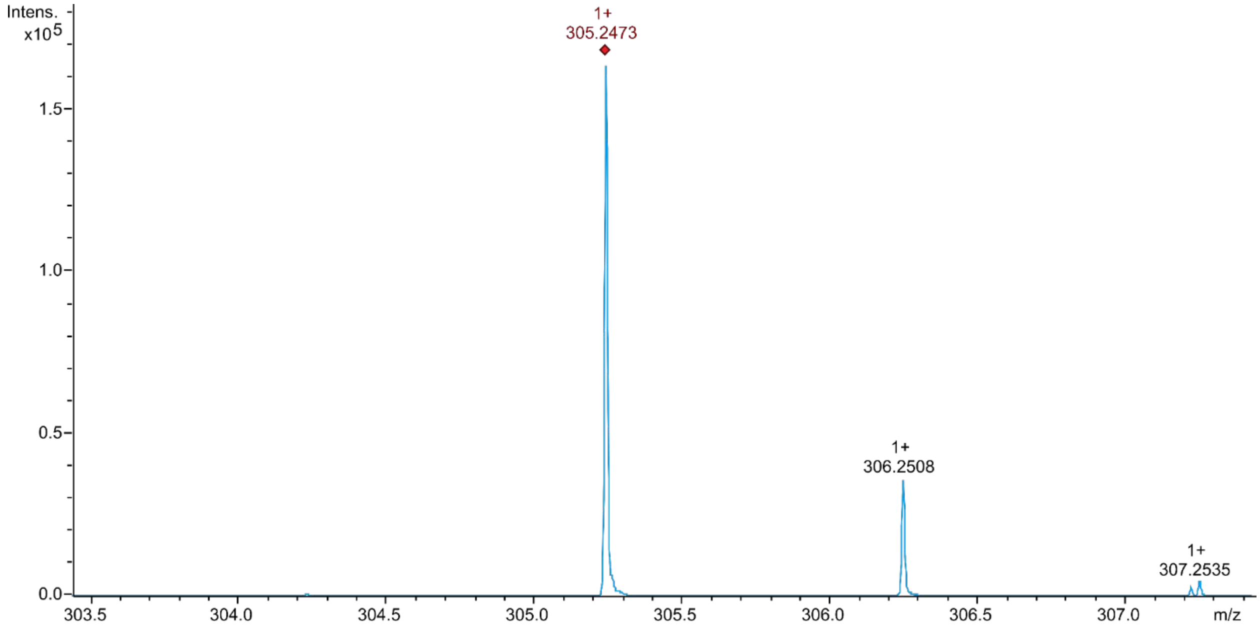

Fig S38: Isotopic pattern of euthailol E (**5**).

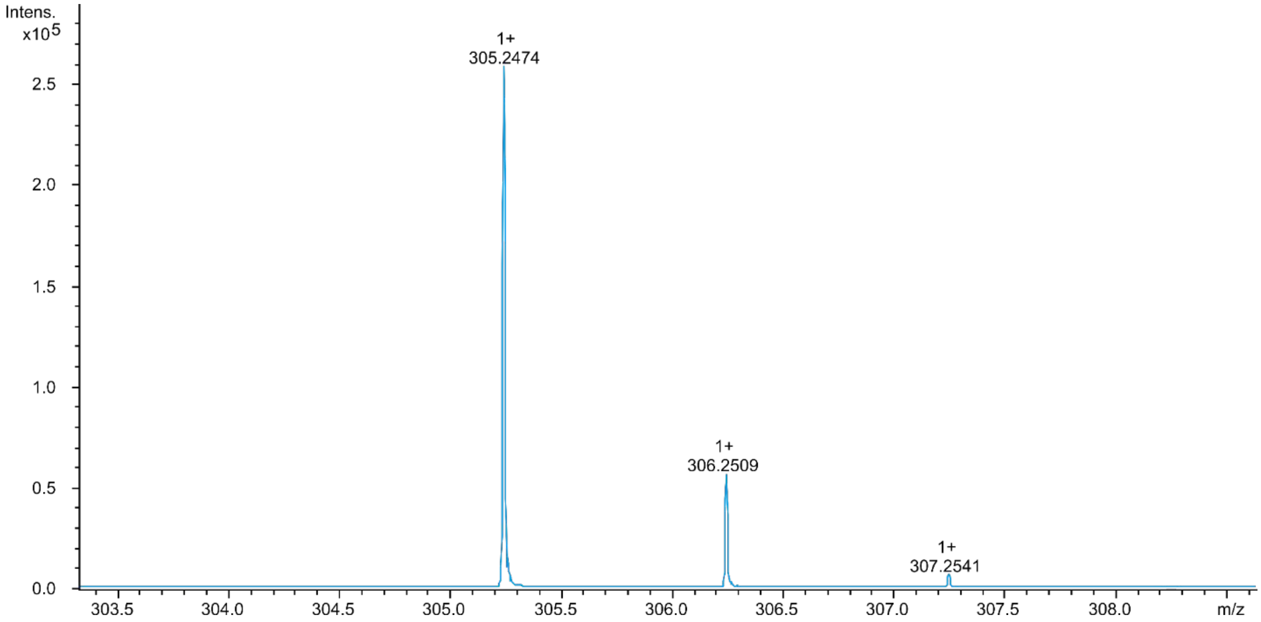

Fig S39: Isotopic pattern of euthailol F (**6**).

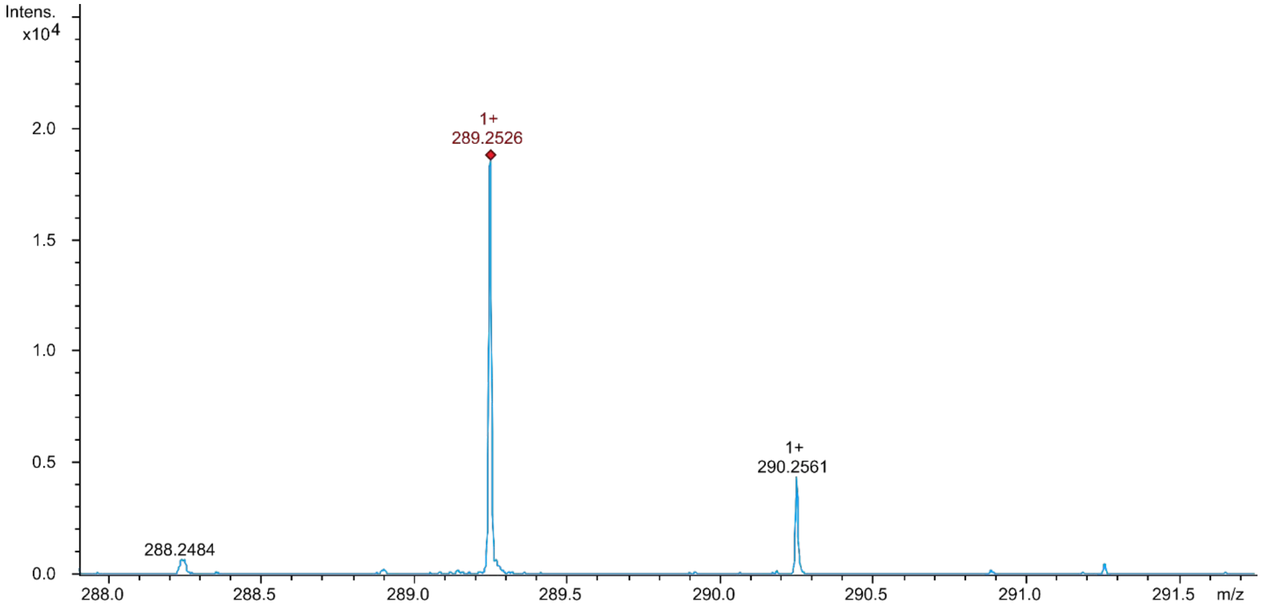

Fig S40: Isotopic pattern of euthailol H (**7**, albireticulone A).

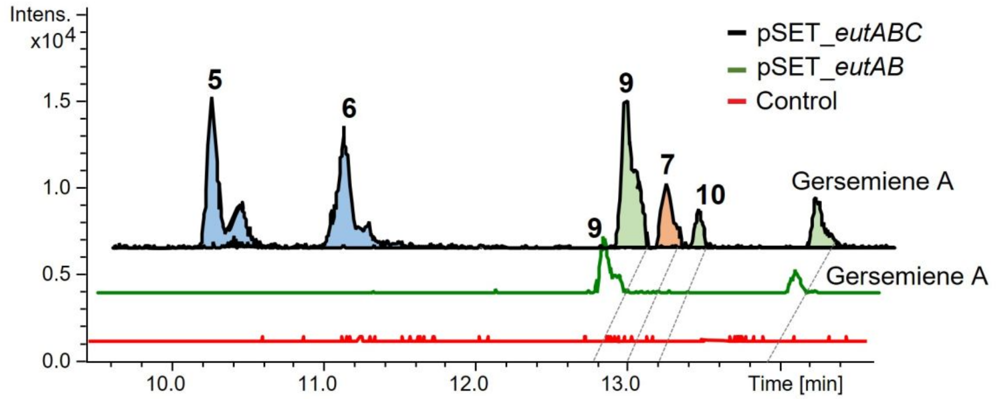

Fig S41: Metabolic profile of *S. albus* strains harboring *eutABC* and *eutAB*. Co-expression of *eutC* with *eutB* results in the isomerization (compound **10**) and oxidation of the isomerized hydrocarbon scaffold (compounds **5–7**). *S. albus* harboring the empty plasmid served as a negative control. EIC of euthailol E (**5**) and F (**6**) at *m/z* 305.2474 $[M+H]^+$, euthailol G (**7**) at *m/z* 289.2526, albireticulene (**9**) and iso-albireticulene (**10**) at *m/z* 273.2577 $[M+H]^+$.

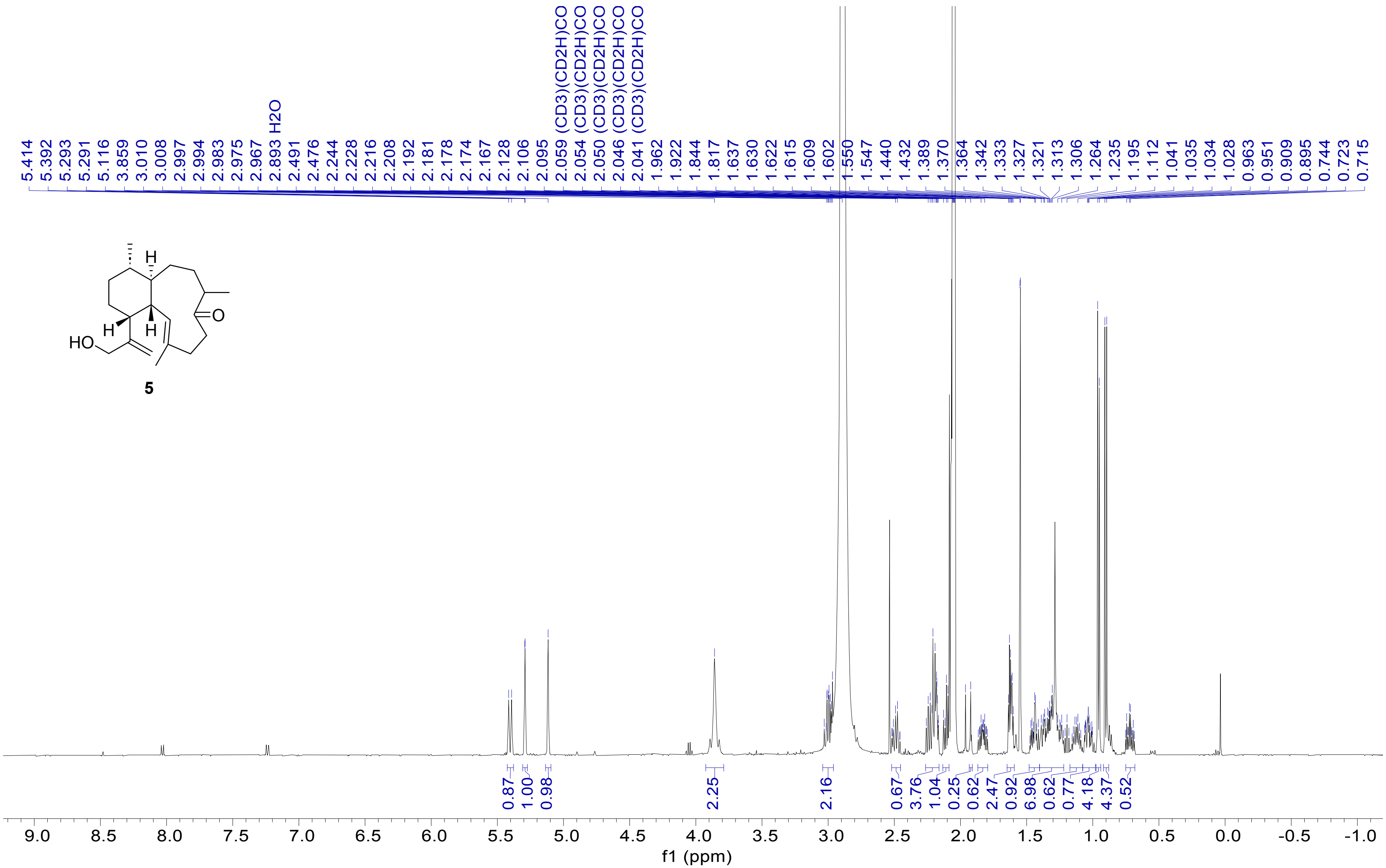

Figure S42. ^{1}H NMR spectrum (500.18 MHz, 298K) of euthailol E (**5**) in $(CD_3)_2CO$.

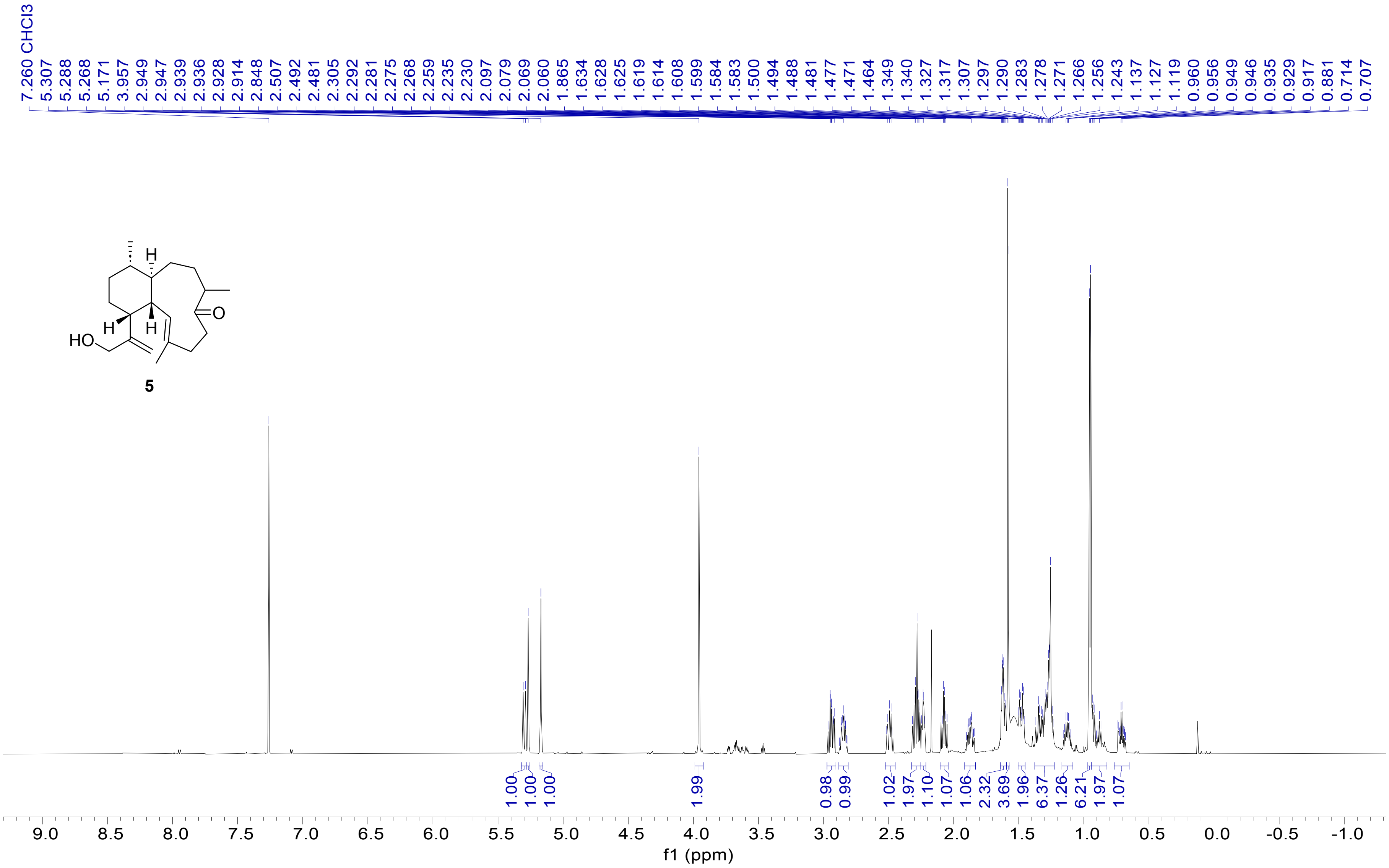

Figure S43. ^{1}H NMR spectrum (600.21 MHz, 298K) of euthailol E (**5**) in $CDCl_3$.

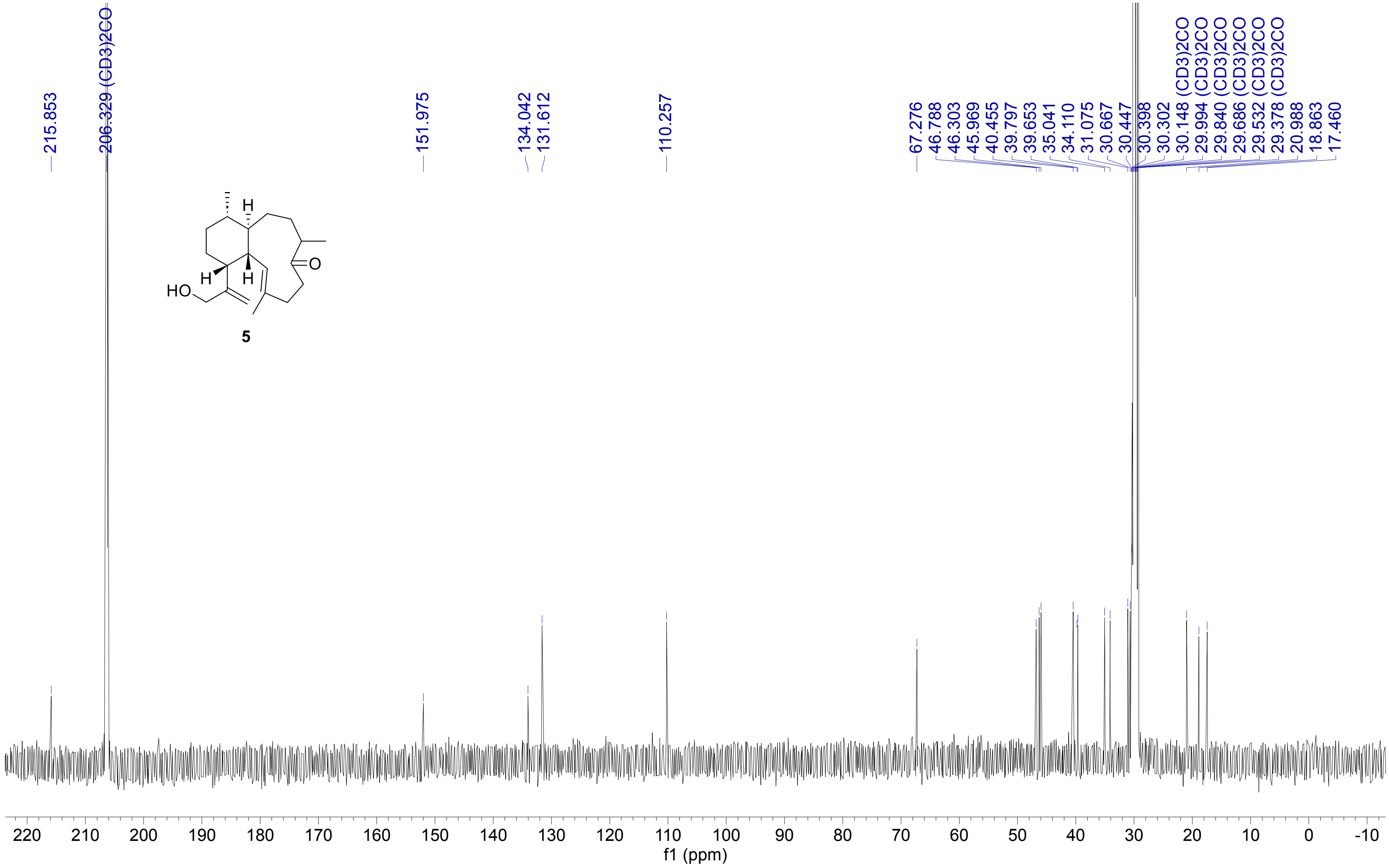

Figure S44. $^{13}C\{^{1}H\}$ NMR spectrum (125.78 MHz, 298K) of euthailol E (**5**) in $(CD_3)_2CO$.

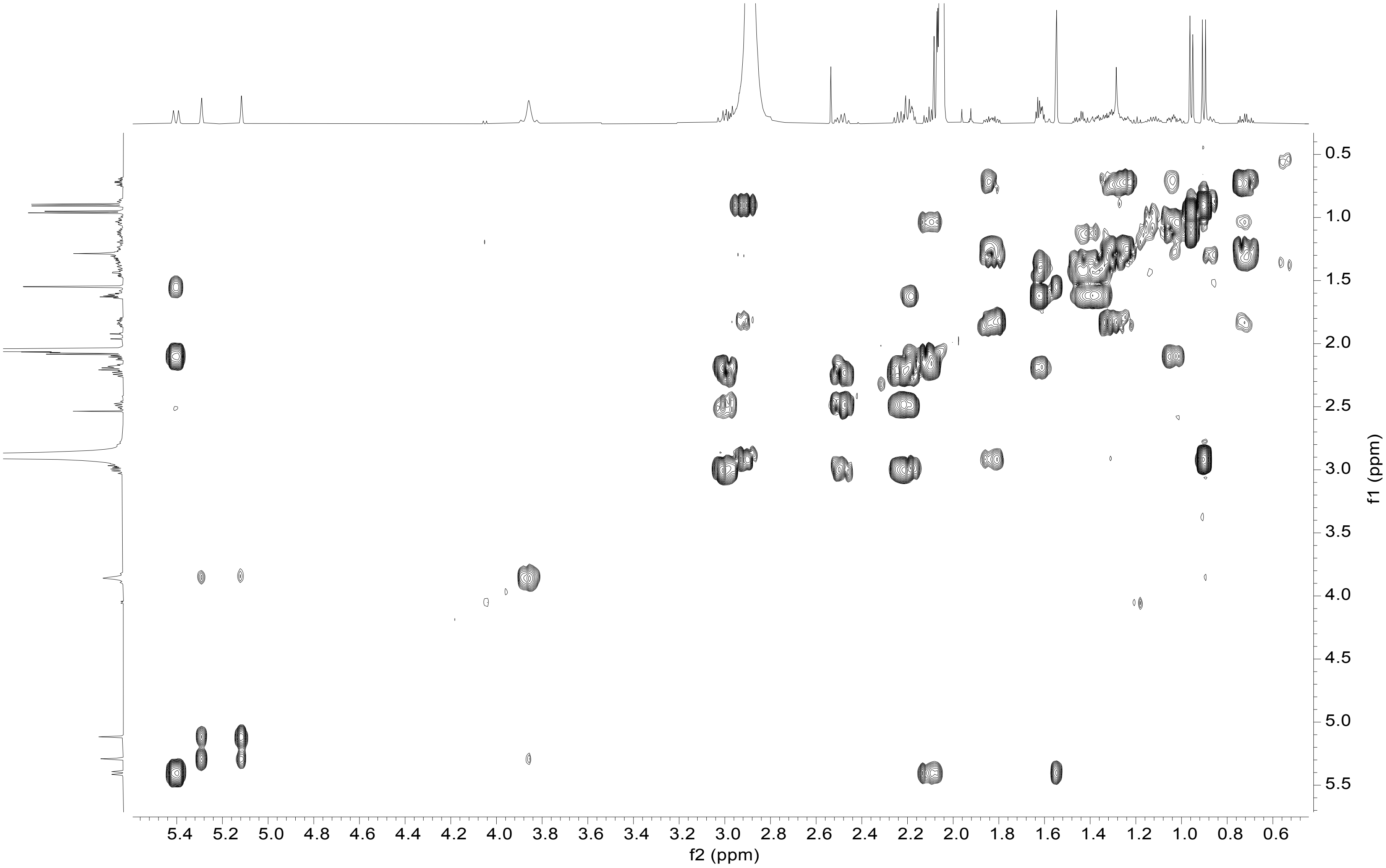

Figure S45. ^{1}H–^{1}H COSY spectrum (298K) of euthailol E (**5**) in $(CD_3)_2CO$.

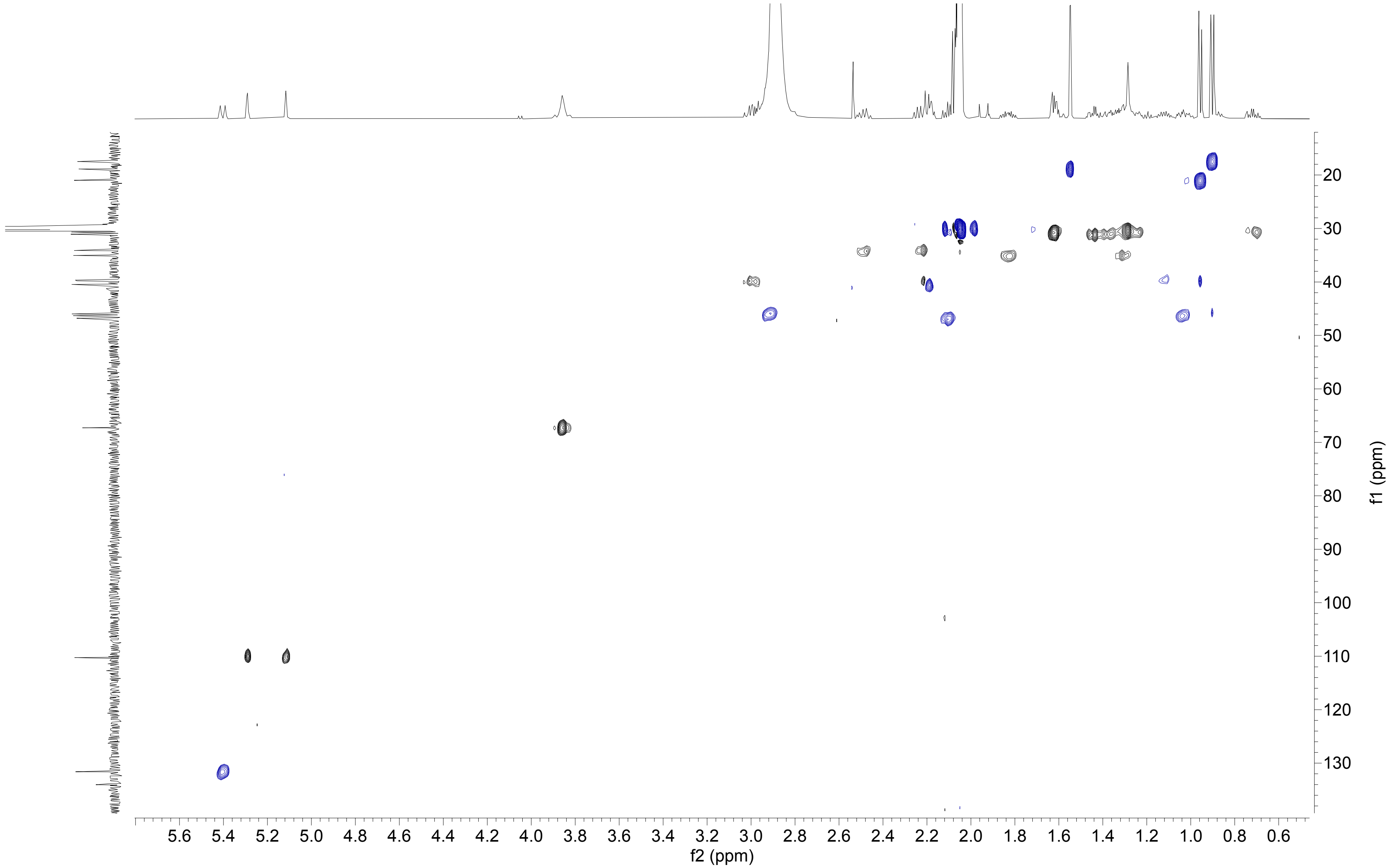

Figure S46. HSQC spectrum (298K) of euthailol E (**5**) in $(CD_3)_2CO$.

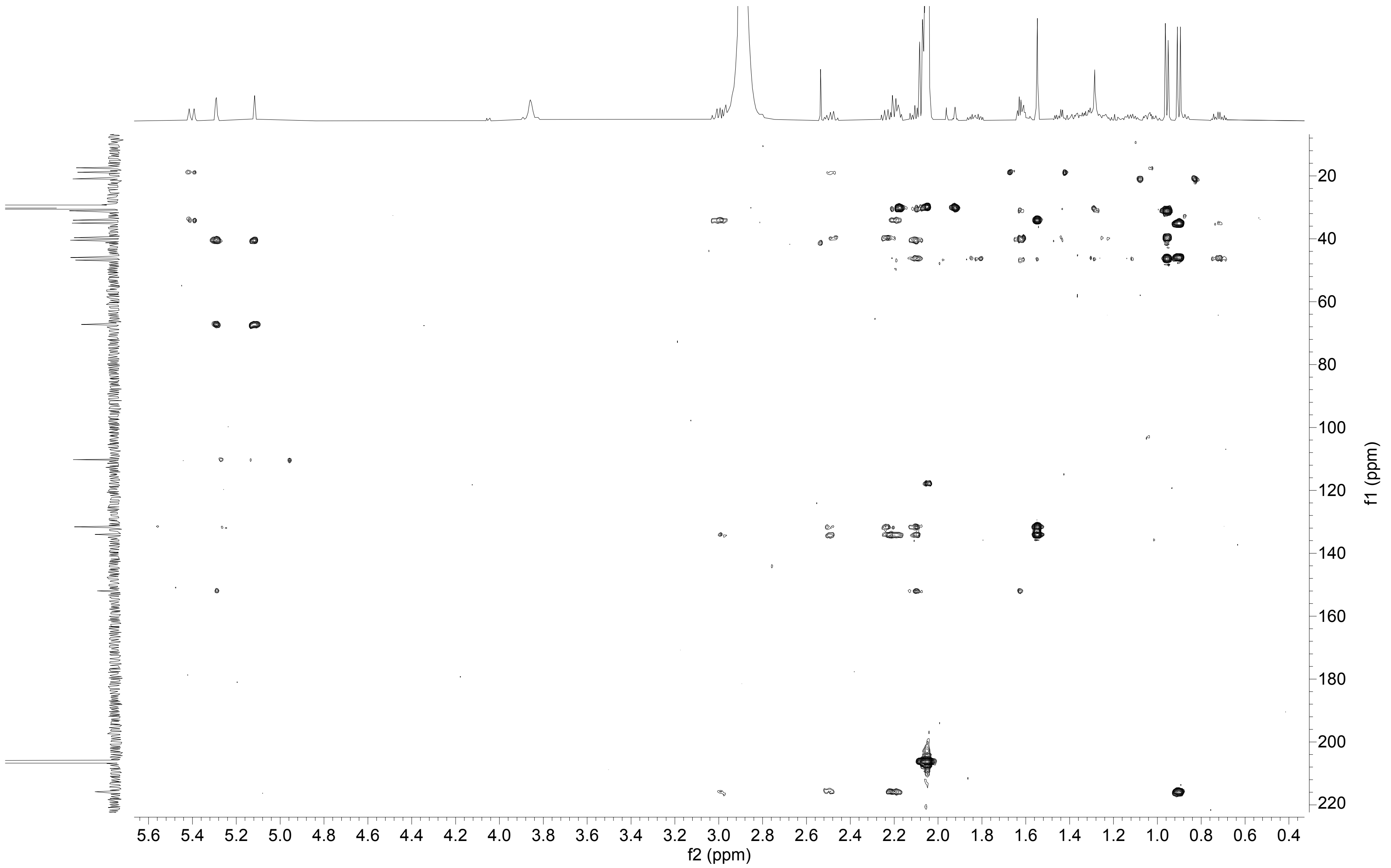

Figure S47. HMBC spectrum (298K) of euthailol E (**5**) in $(CD_3)_2CO$.

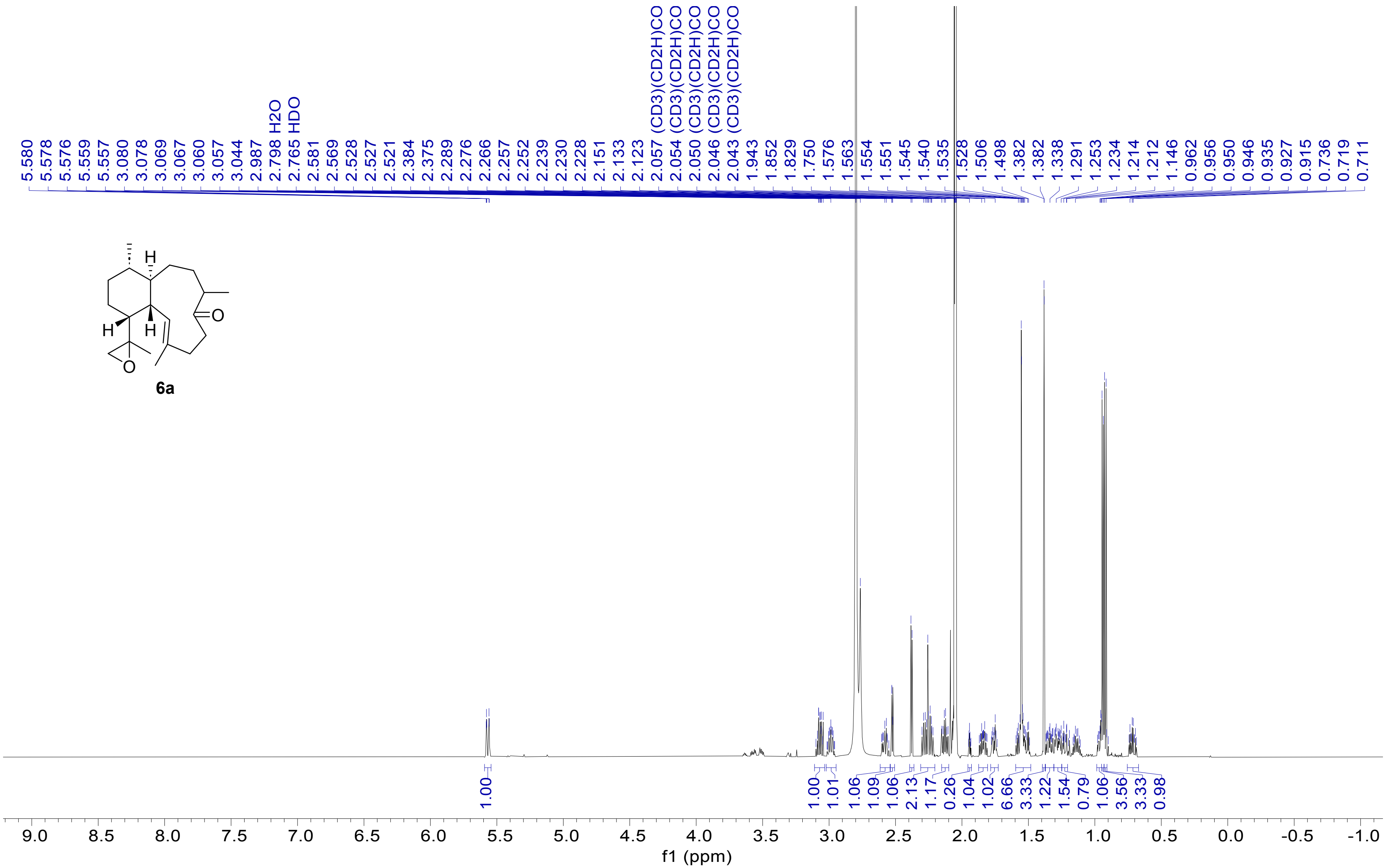

Figure S48. ^{1}H NMR spectrum (600.21 MHz, 298K) of euthailol F (**6a**) in $(CD_3)_2CO$.

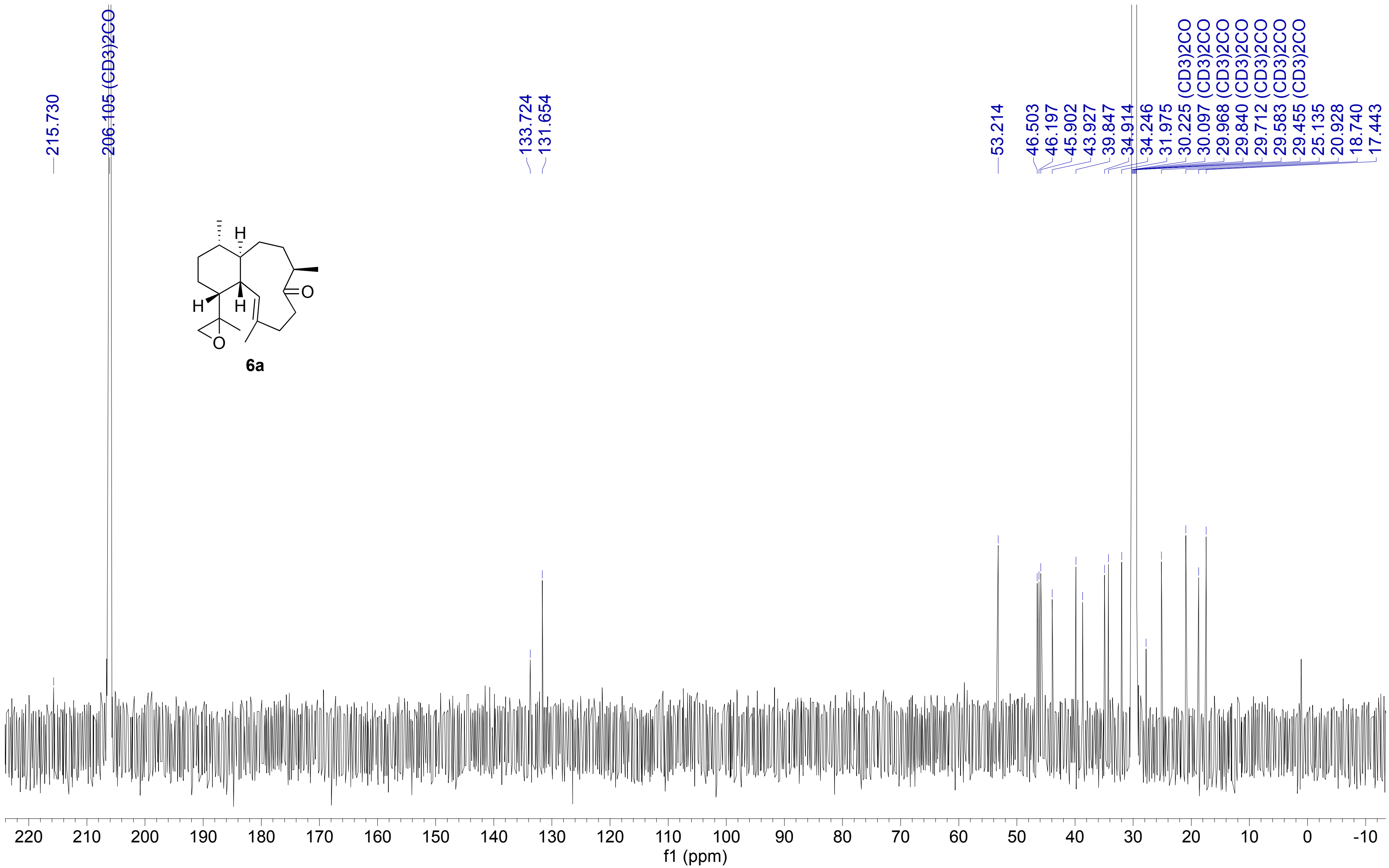

Figure S49. $^{13}C\{^{1}H\}$ NMR spectrum (150.94 MHz, 298K) of euthailol F (**6a**) in $(CD_3)_2CO$.

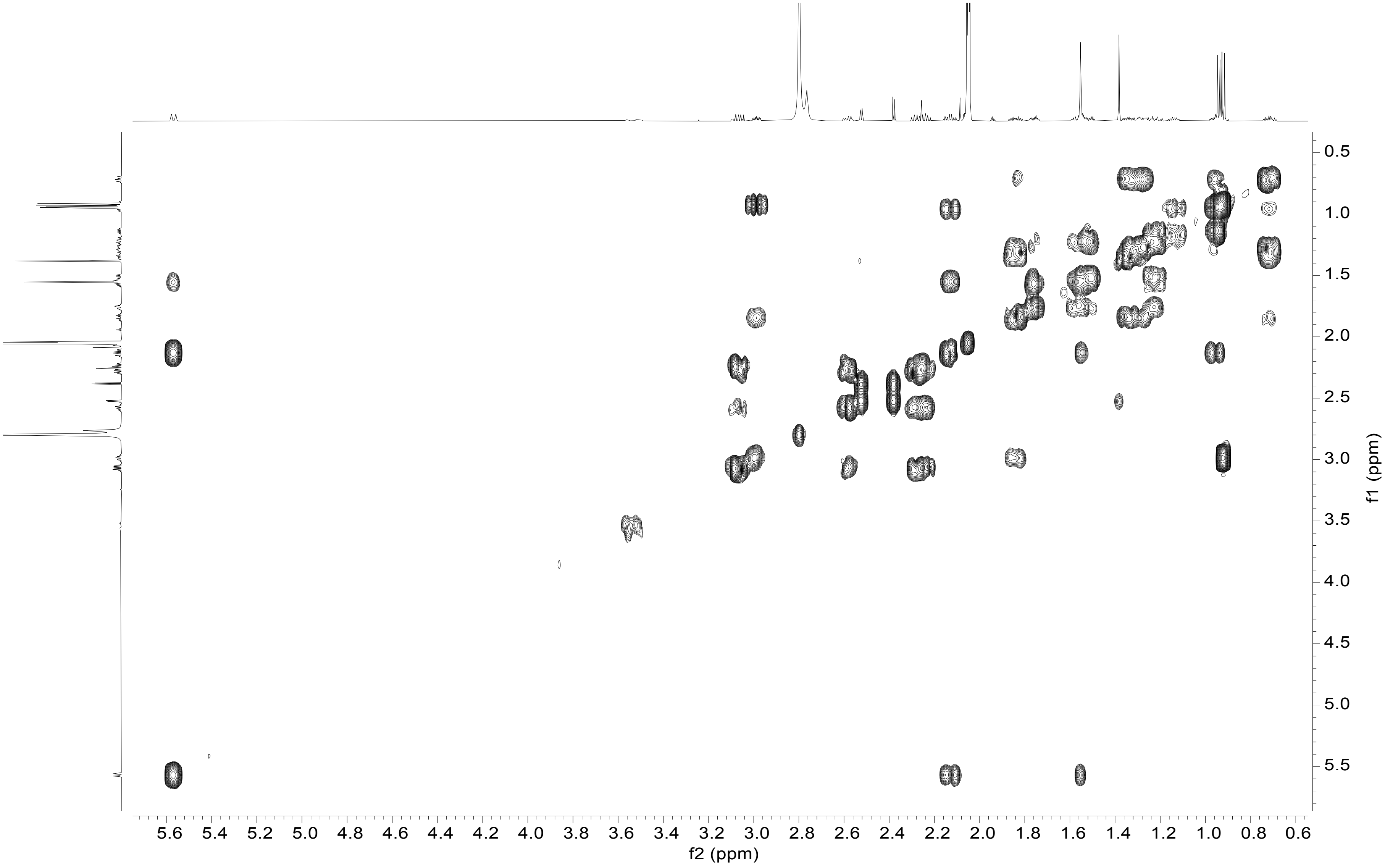

Figure S50. ^{1}H–^{1}H COSY spectrum (298K) of euthailol F (**6a**) in $(CD_3)_2CO$.

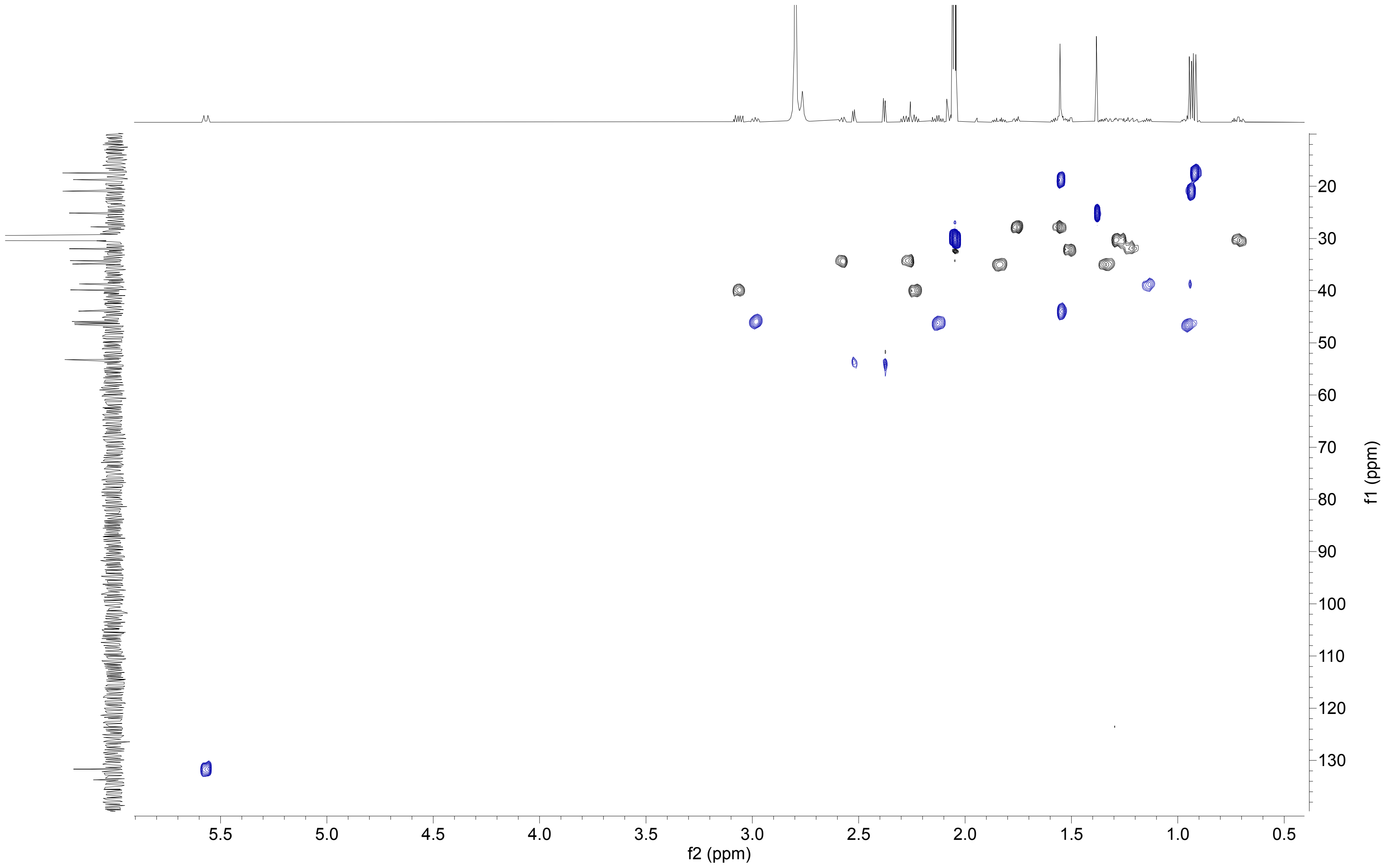

Figure S51. HSQC spectrum (298K) of euthailol F (**6a**) in $(CD_3)_2CO$.

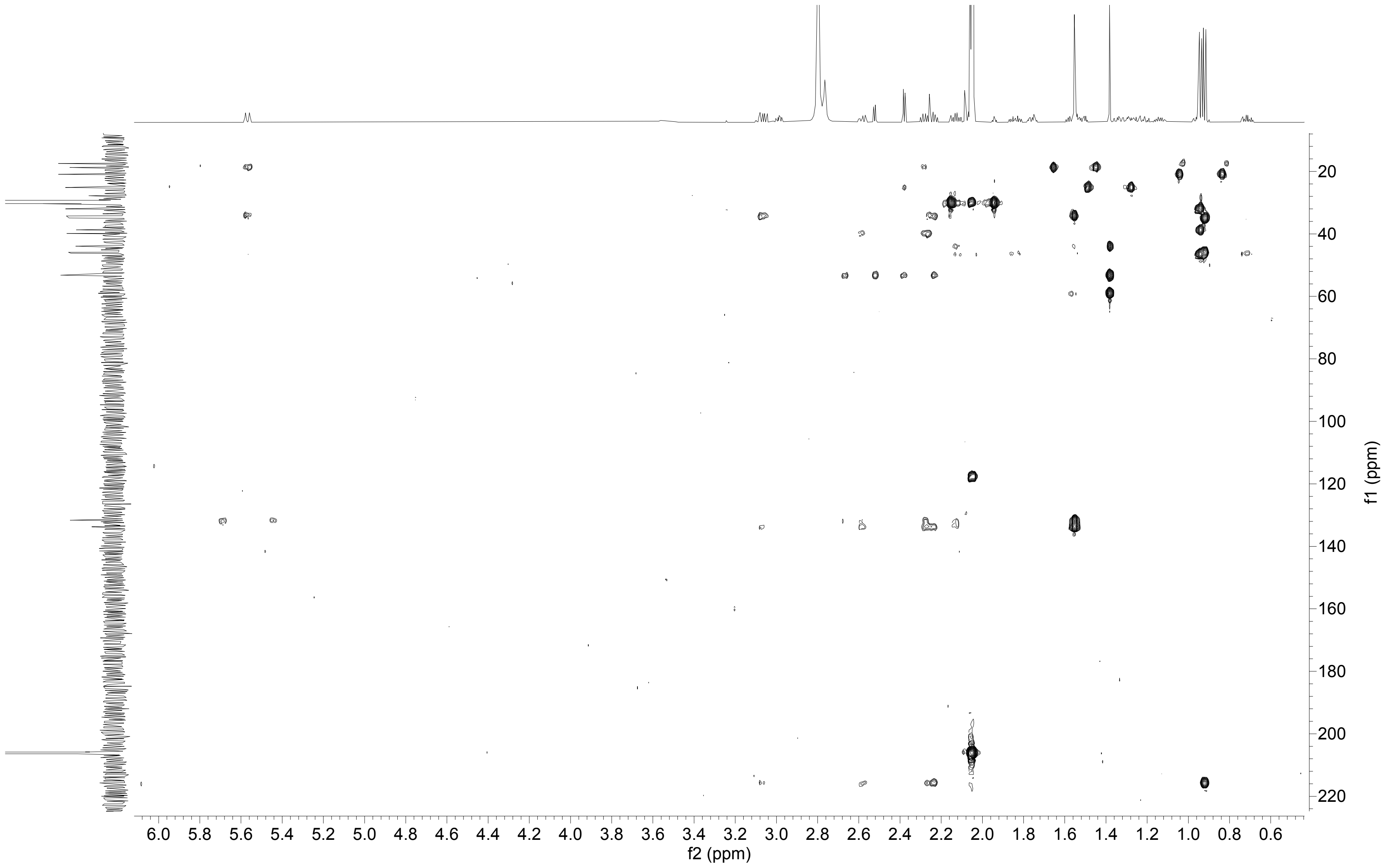

Figure S52. HMBC spectrum (298K) of euthailol F (**6a**) in $(CD_3)_2CO$.

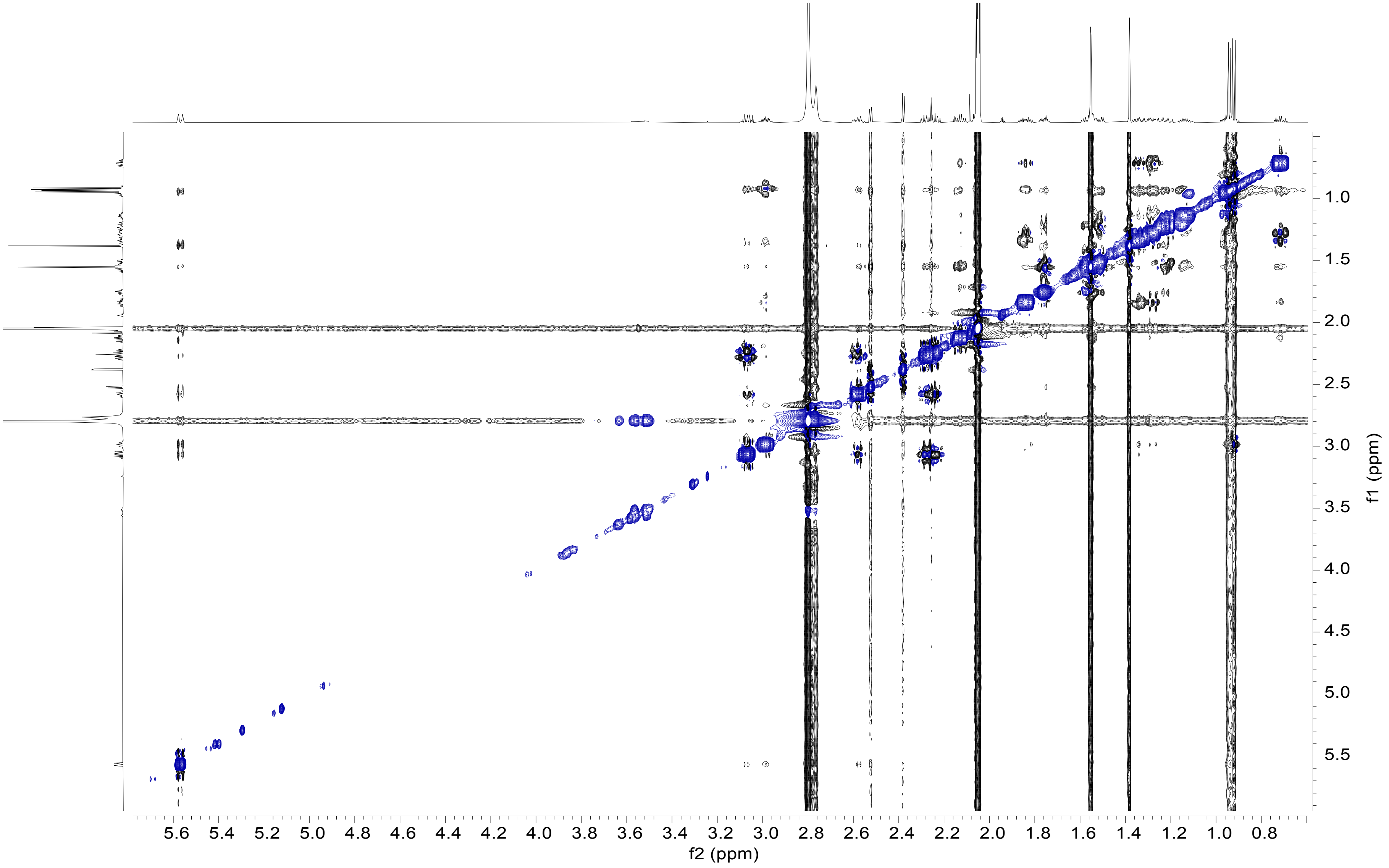

Figure S53. NOESY spectrum (298K) of euthailol F (**6a**) in $(CD_3)_2CO$.

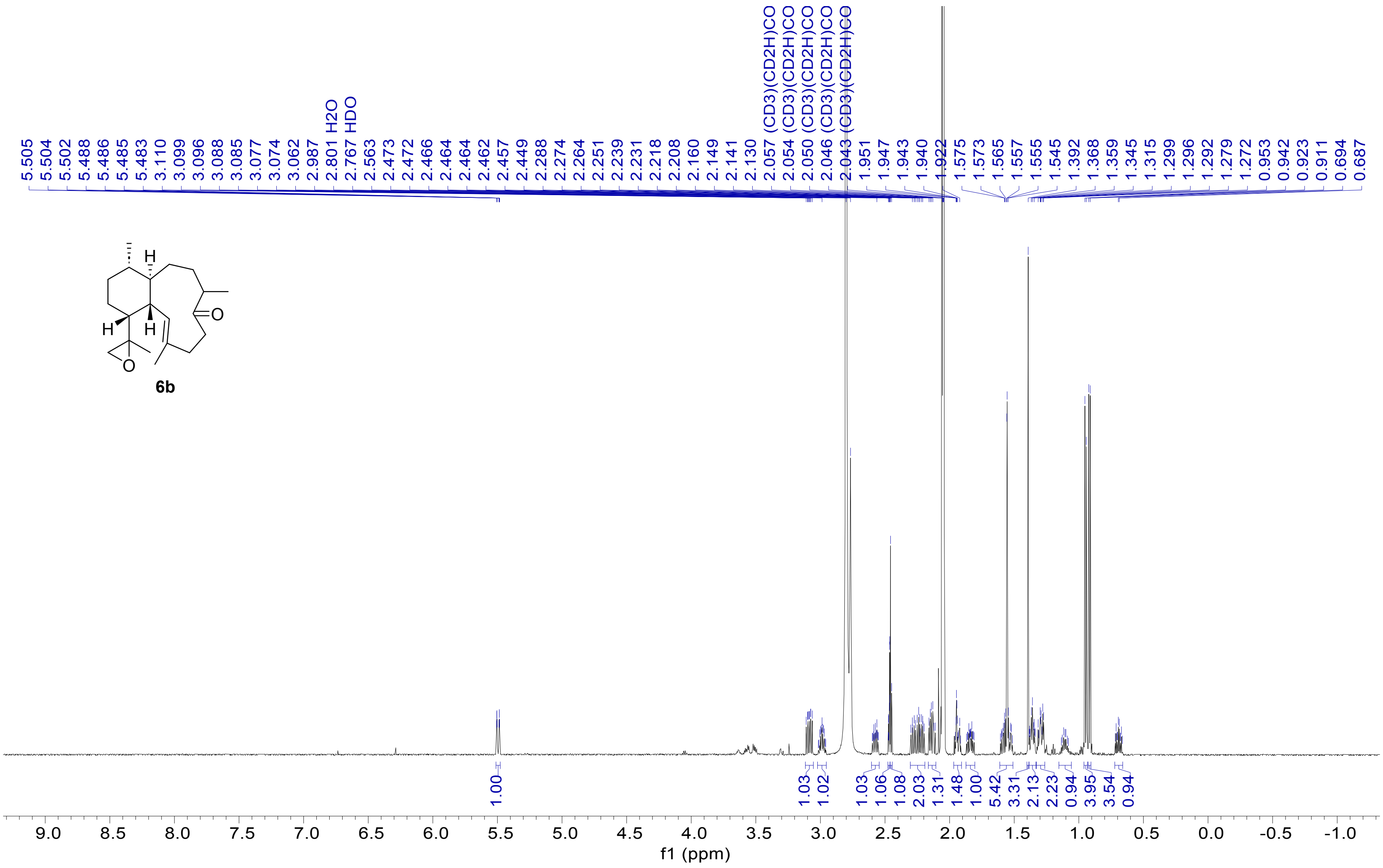

Figure S54. ^{1}H NMR spectrum (600.21 MHz, 298K) of euthailol G (**6b**) in $(CD_3)_2CO$.

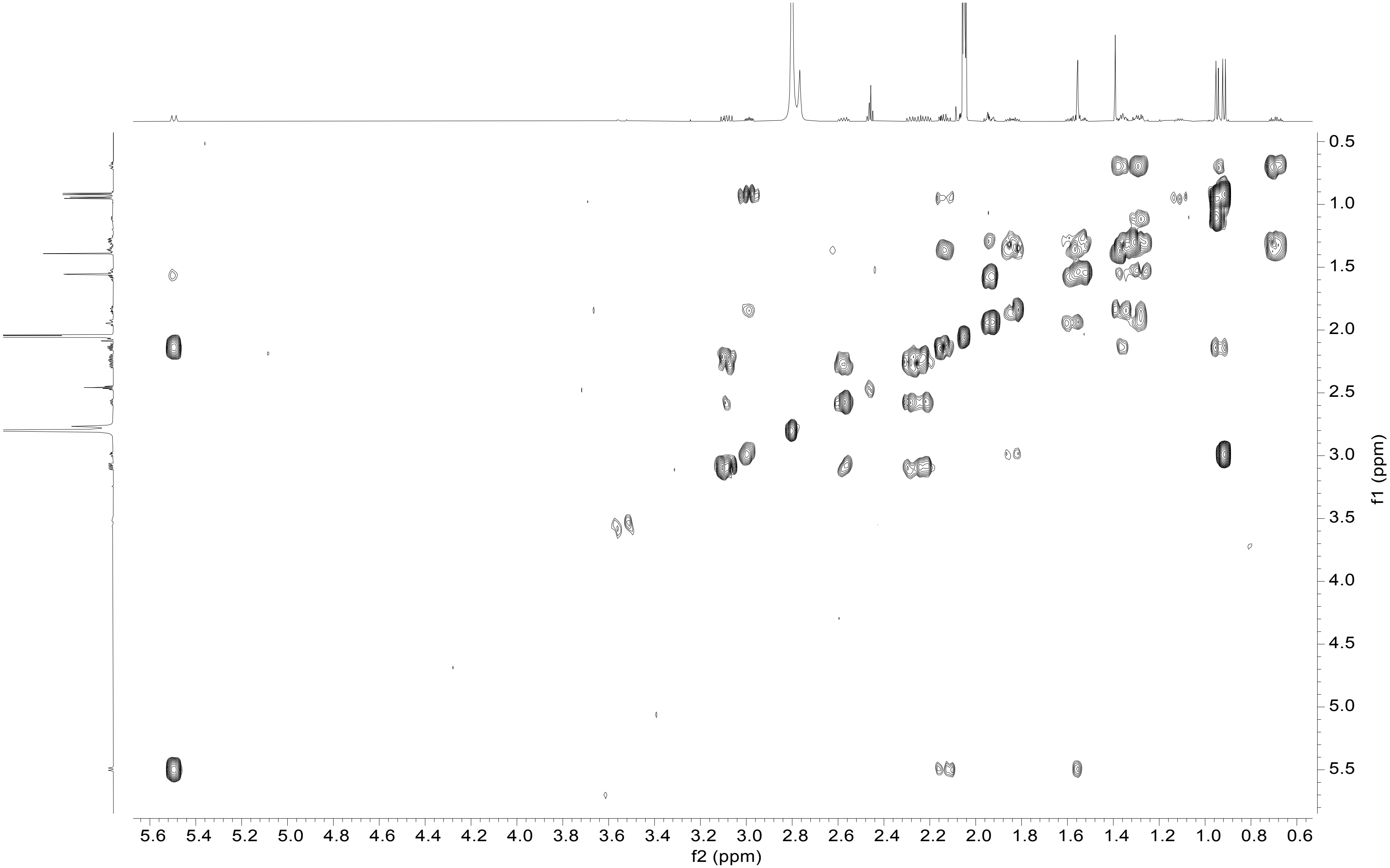

Figure S55. ^1H–^1H COSY spectrum (298K) of euthailol G (**6b**) in $(CD_3)_2CO$.

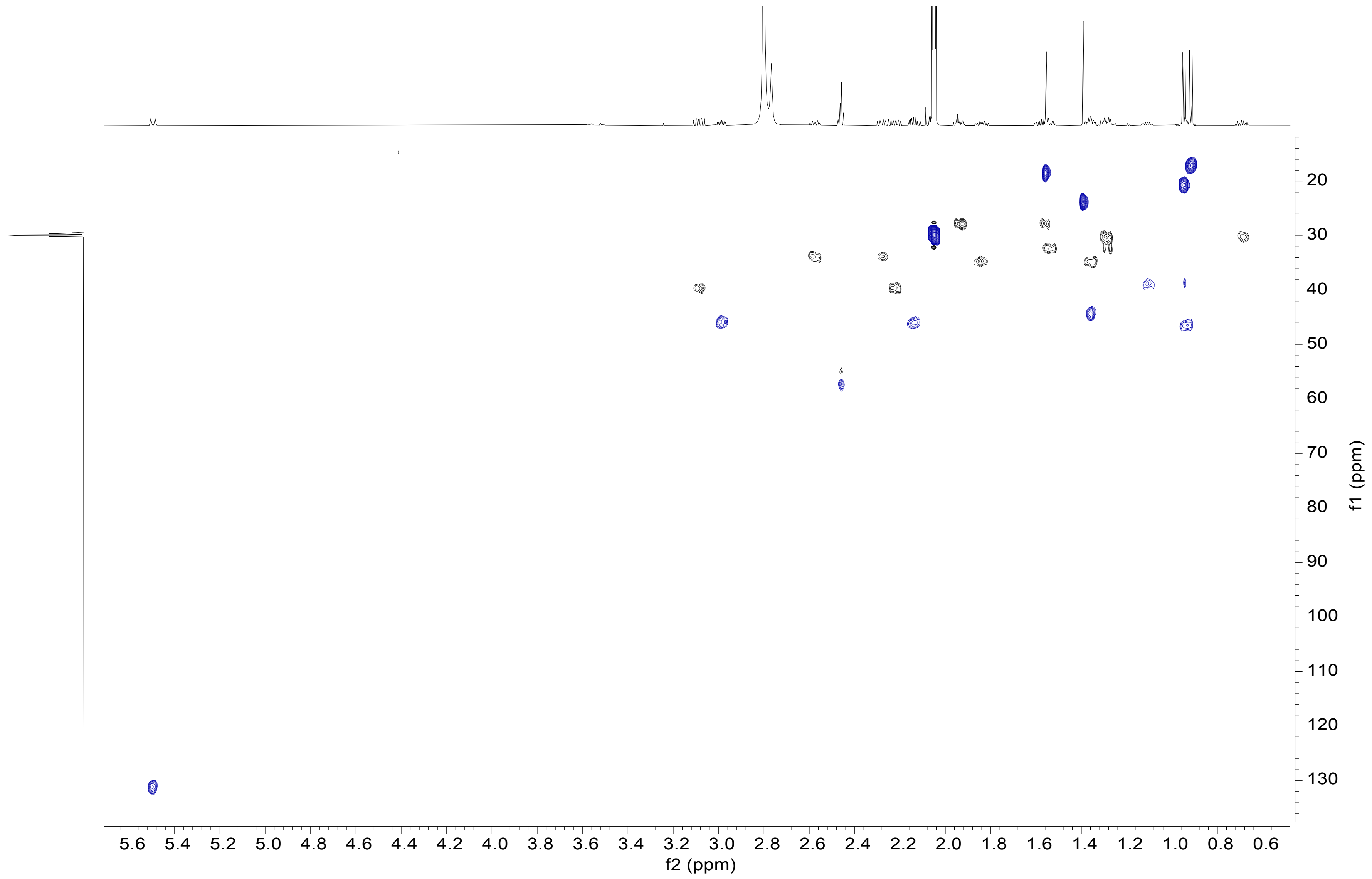

Figure S56. HSQC spectrum (298K) of euthailol G (**6b**) in $(CD_3)_2CO$.

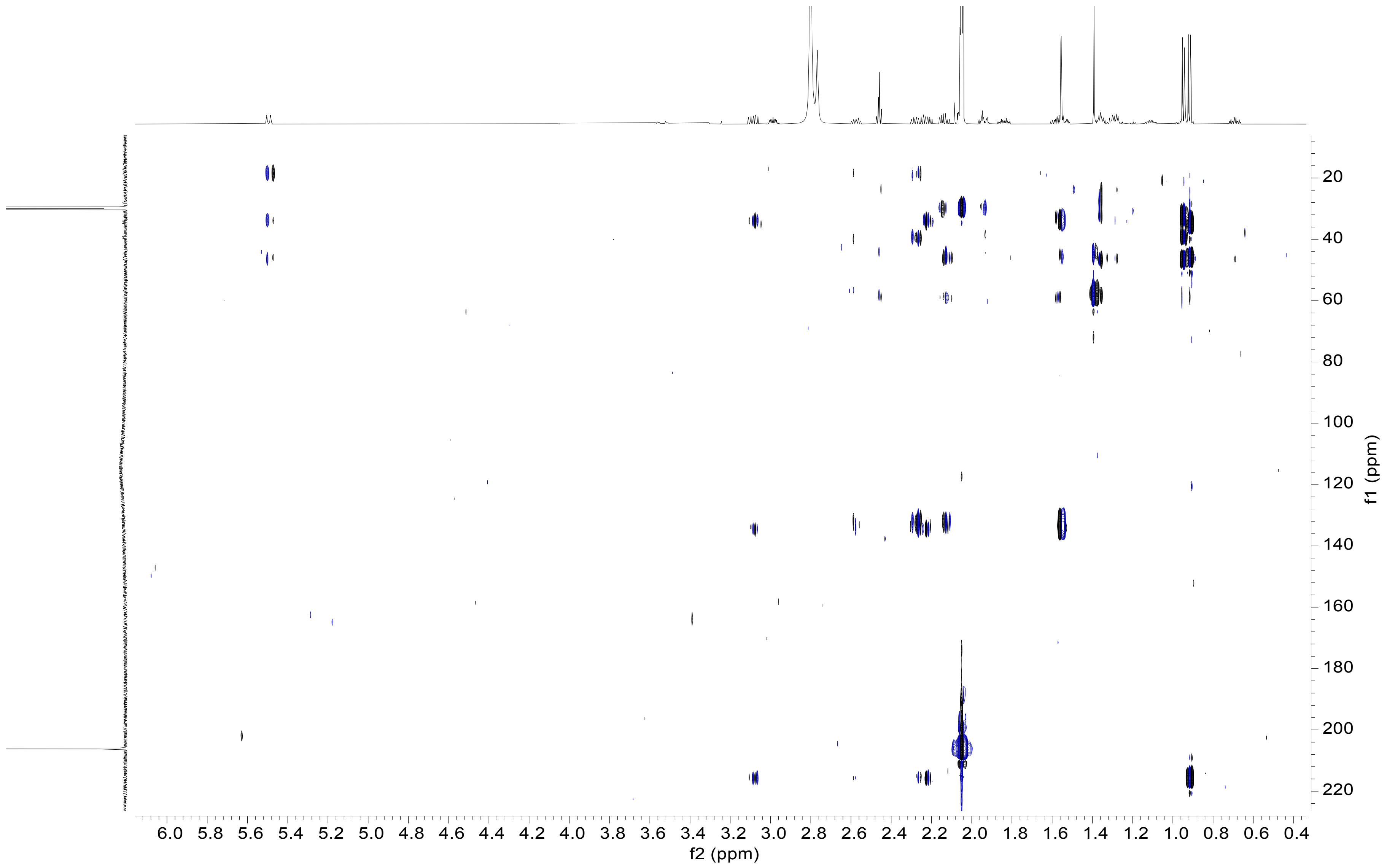

Figure S57. HMBC spectrum (298K) of euthailol G (**6b**) in $(CD_3)_2CO$.

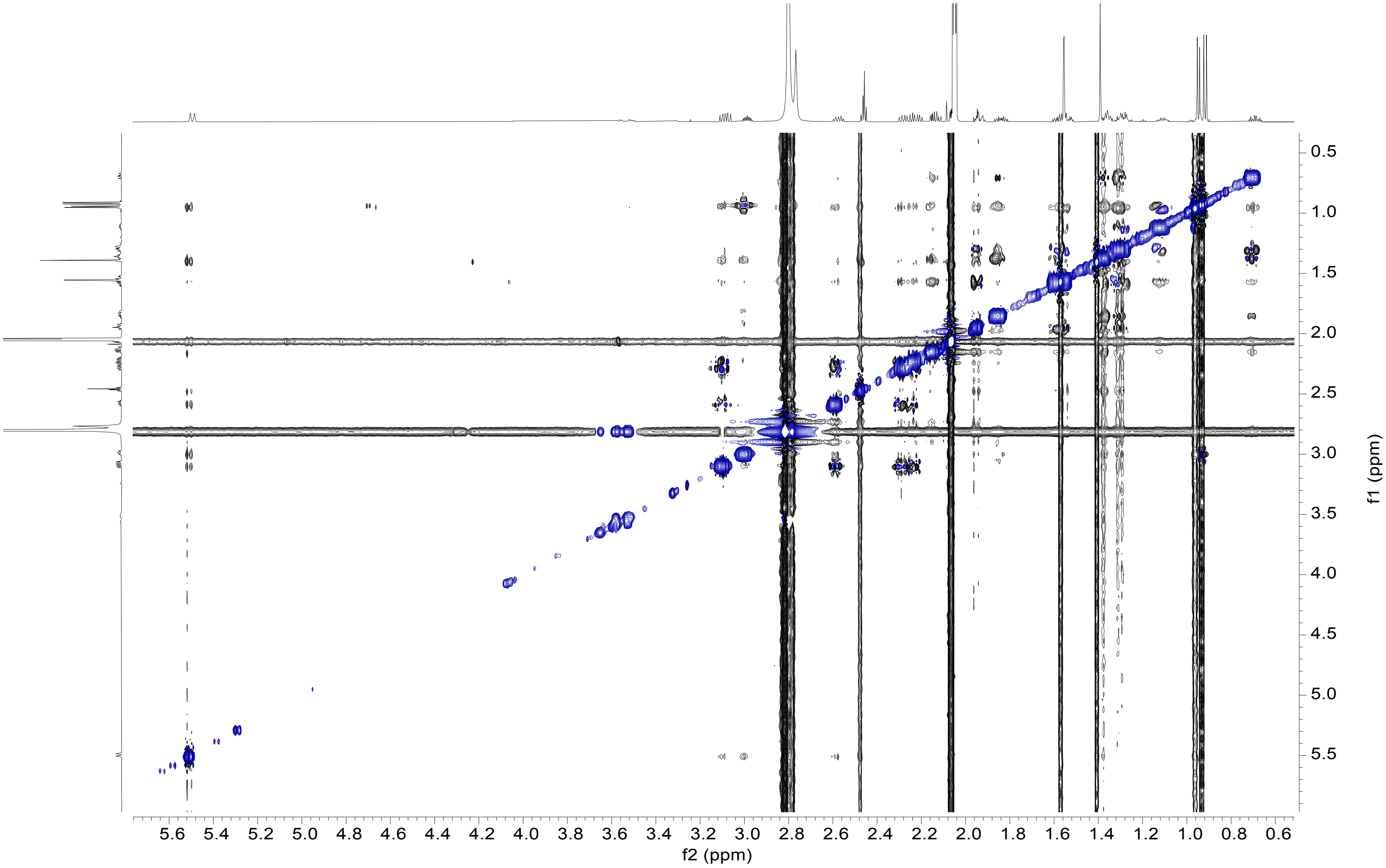

Figure S58. NOESY spectrum (298K) of euthailol G (**6b**) in $(CD_3)_2CO$.

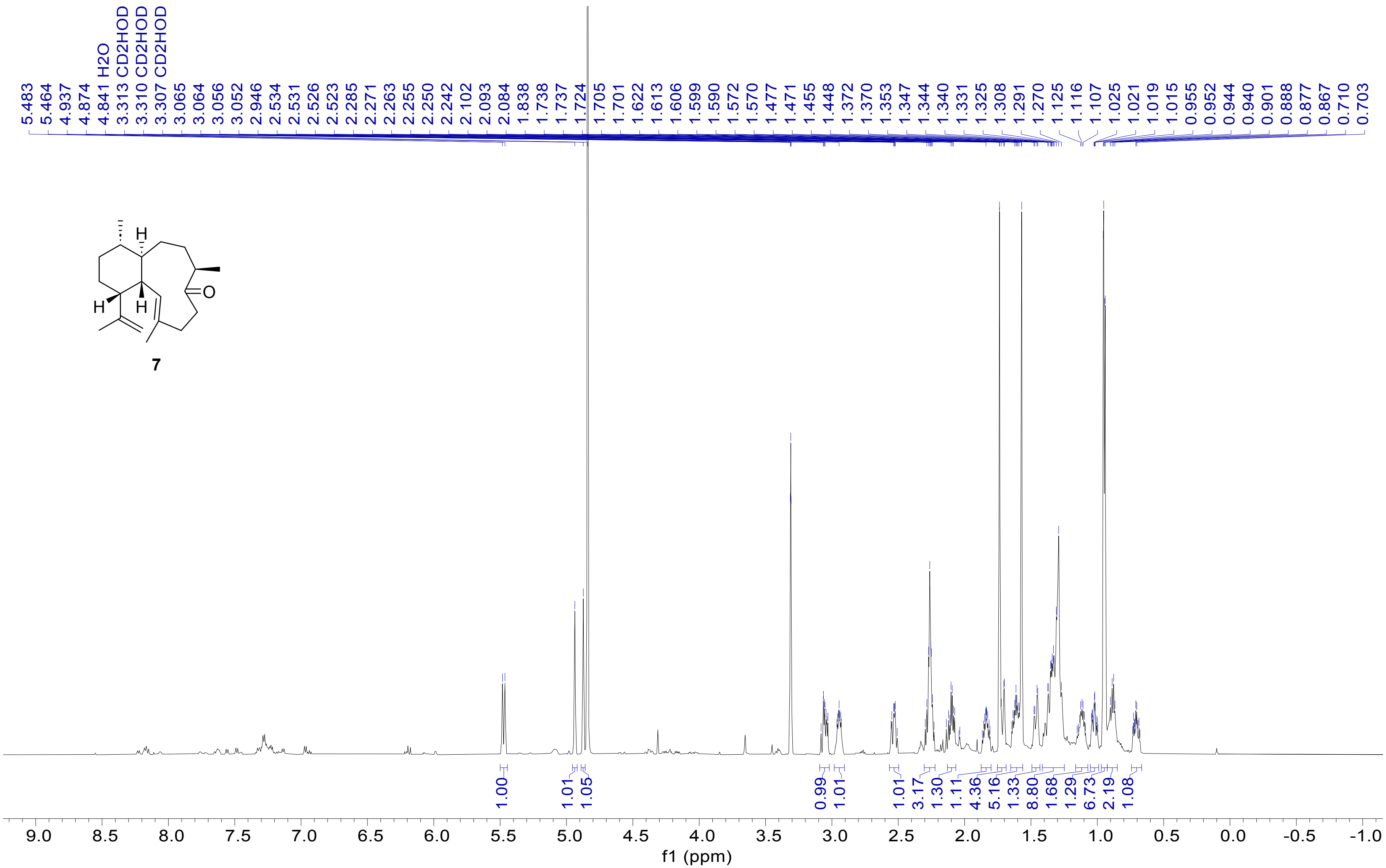

Figure S59. ^{1}H NMR spectrum (600.21 MHz, 298K) of euthailol H (**7**, albireticulone A) in CD_3OD.

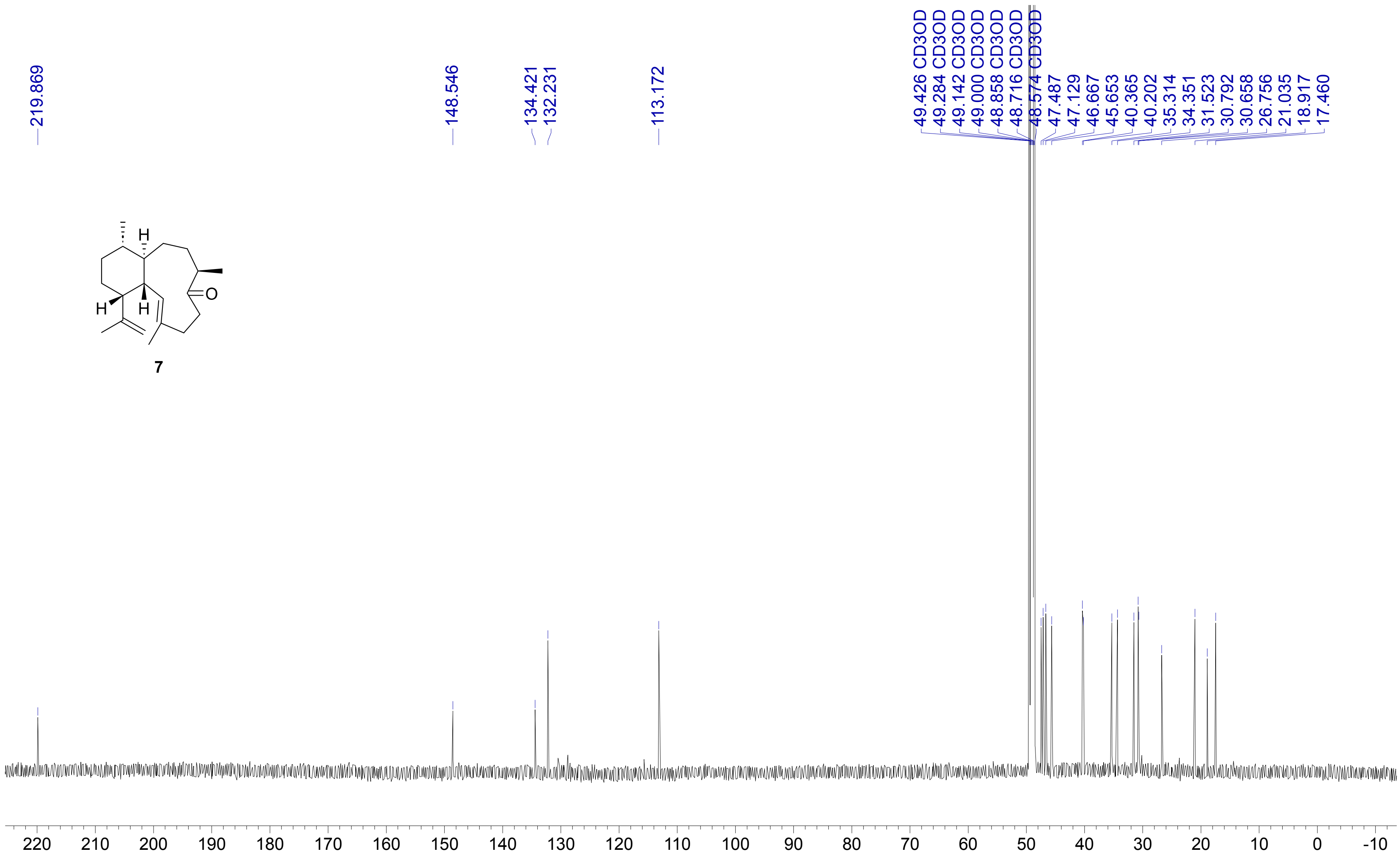

Figure S60. $^{13}C\{^1H\}$ NMR spectrum (150.94 MHz, 298K) of euthailol H (**7**, albireticulone A) in CD_3OD.

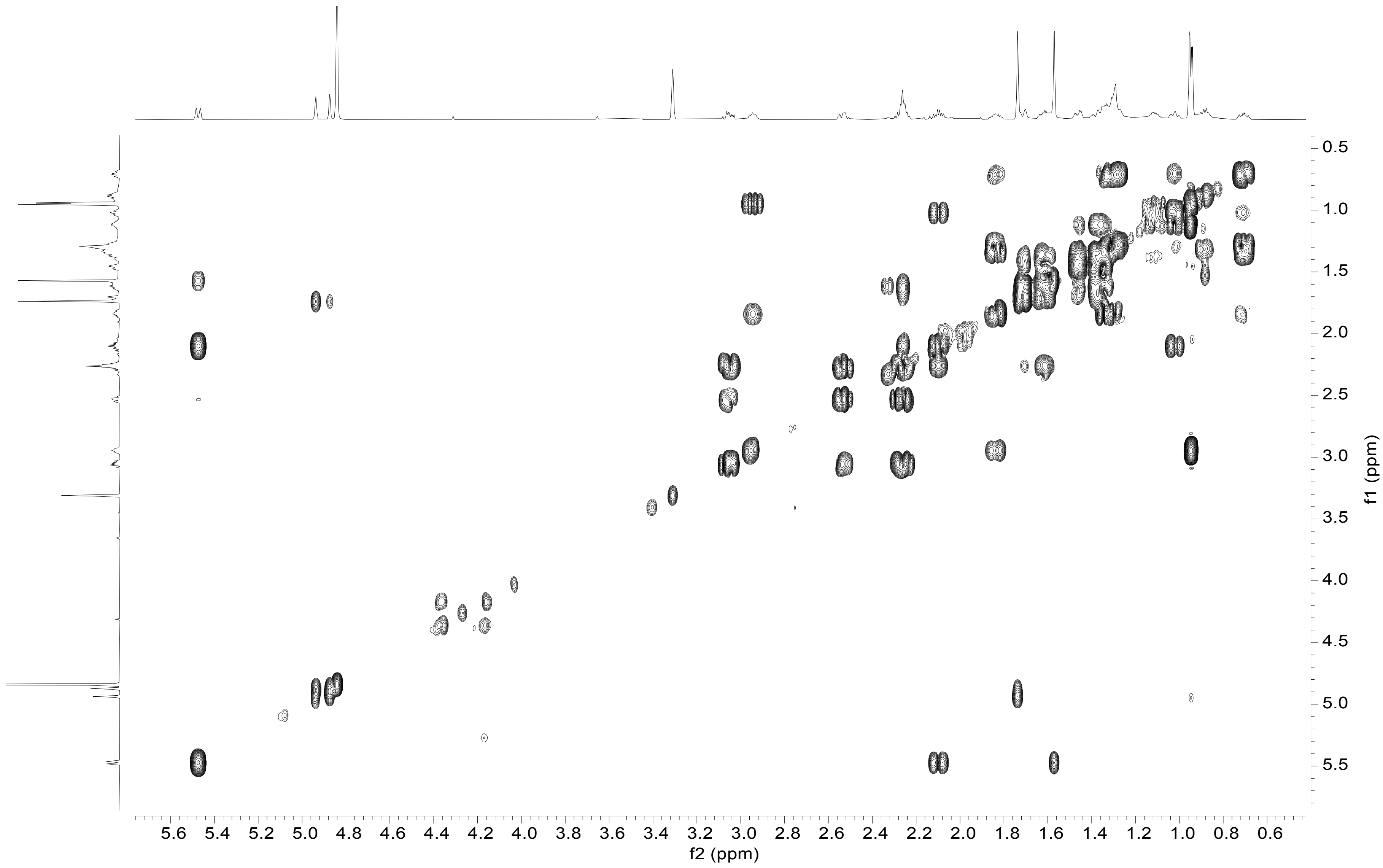

Figure S61. ^{1}H–^{1}H COSY spectrum (298K) of euthailol H (**7**, albireticulone A) in CD_3OD.

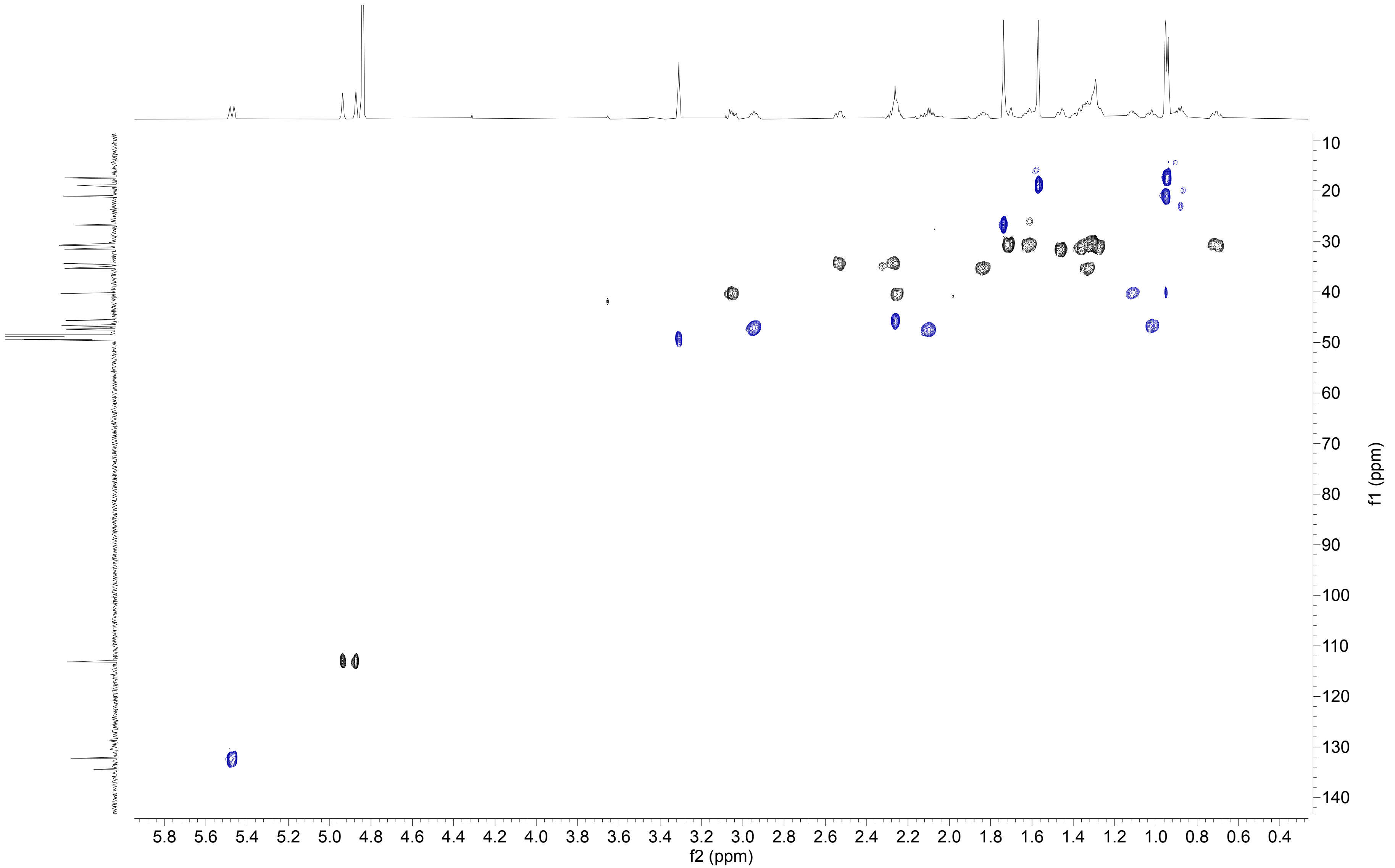

Figure S62. HSQC spectrum (298K) of euthailol H (**7**, albireticulone A) in CD_3OD.

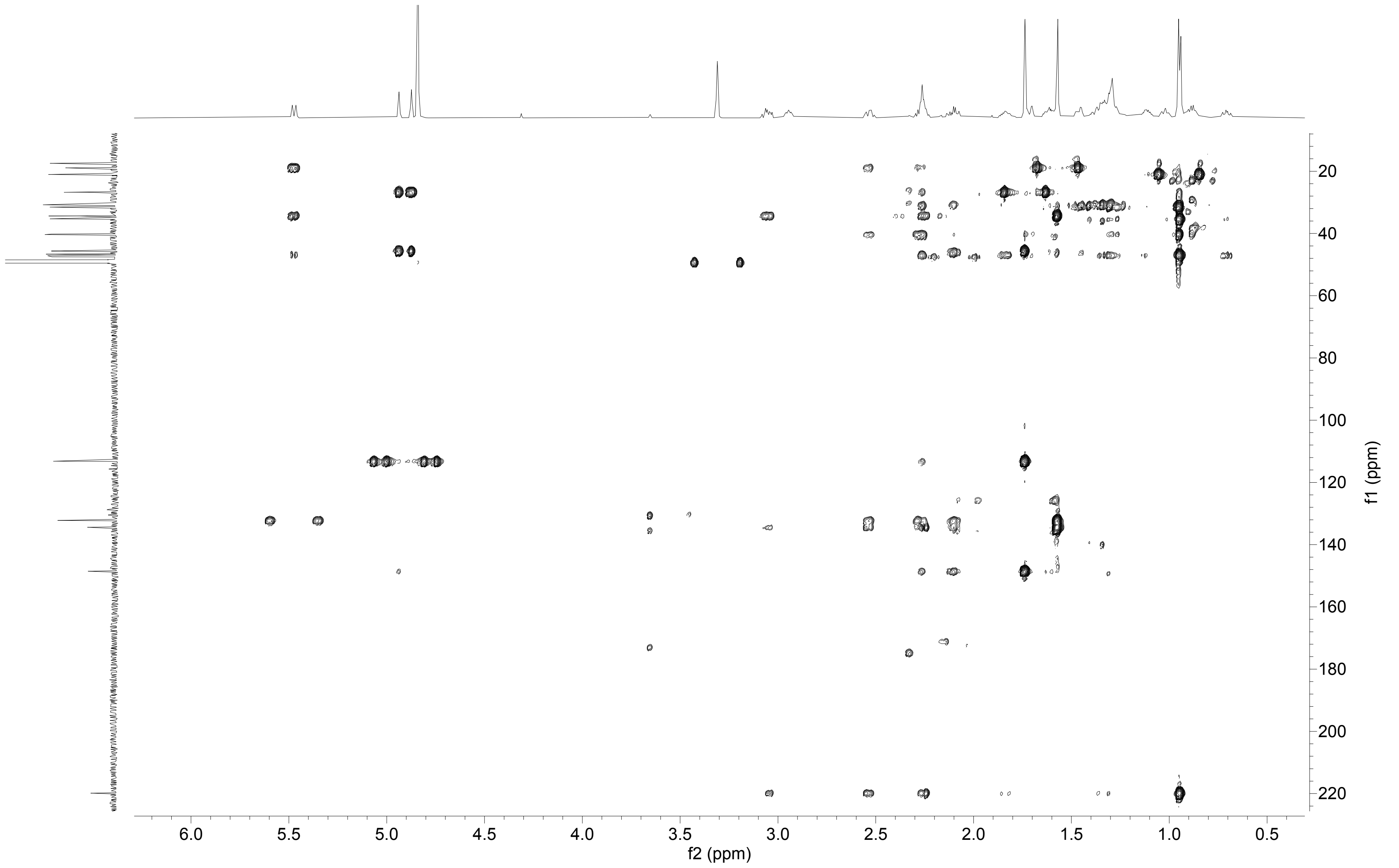

Figure S63. HMBC spectrum (298K) of euthailol H (**7**, albireticulone A) in CD_3OD.

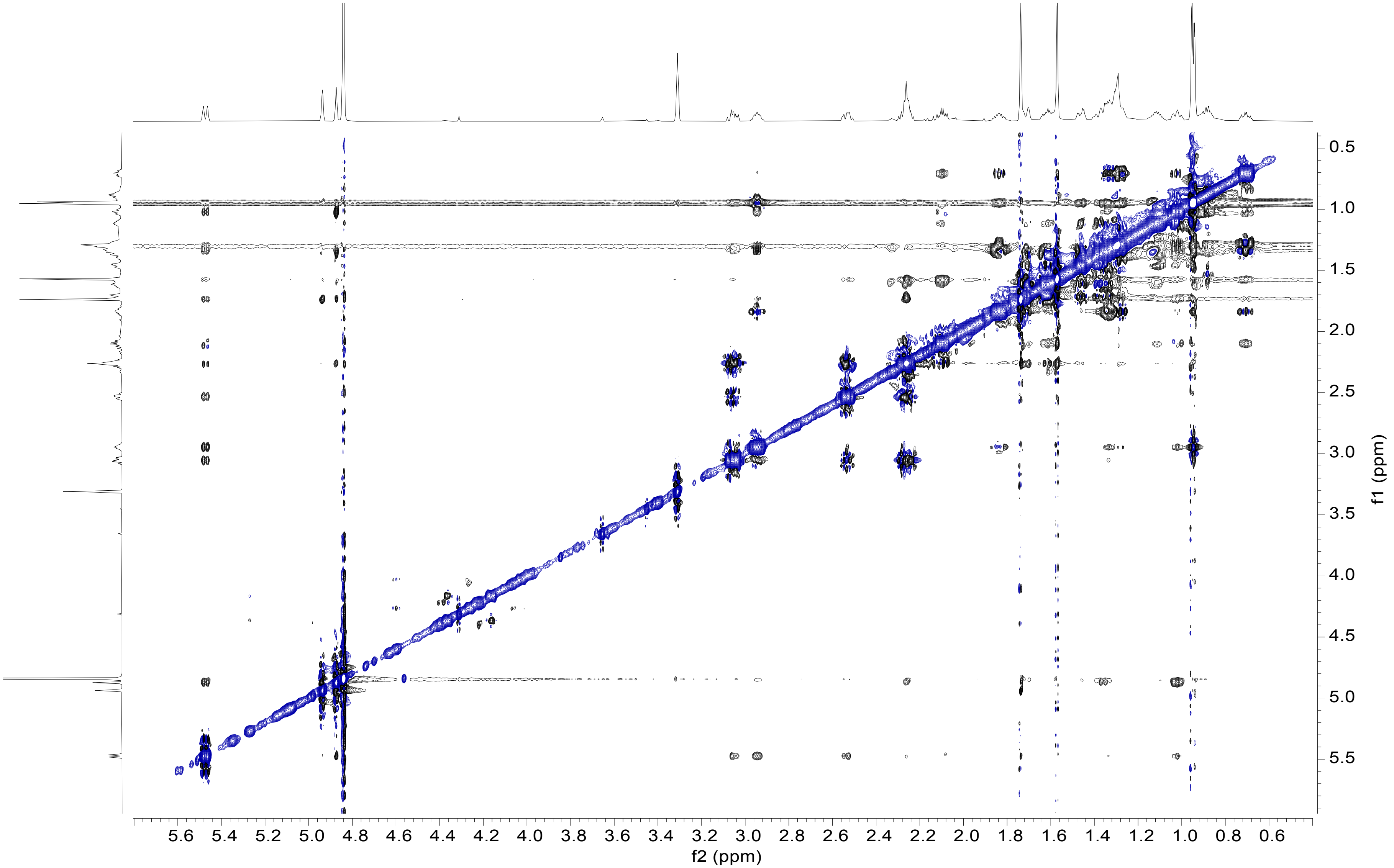

Figure S64. NOESY spectrum (298K) of euthailol H (**7**, albireticulone A) in CD_3OD.

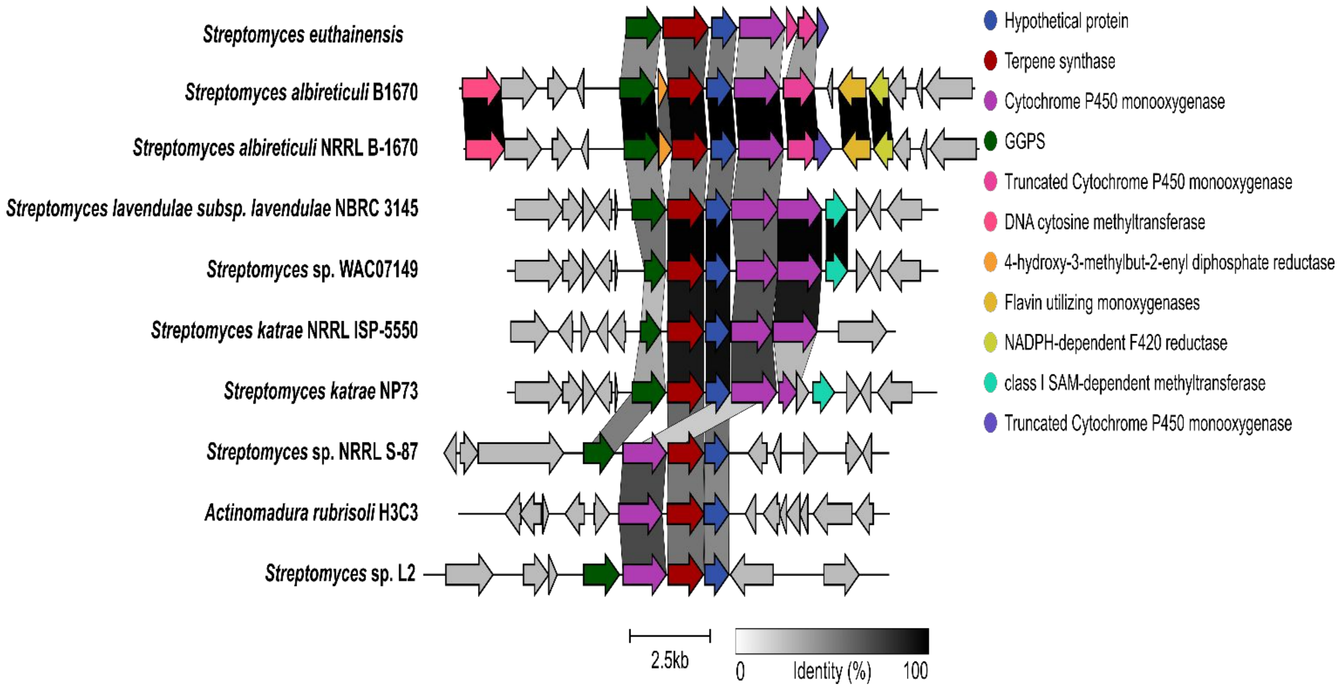

Figure S65: Comparative gene cluster analysis of *eut* BGC with terpene BGCs encoding hypothetical proteins with homology to EutC.

Table S1. ^{1}H (499.63 MHz) and ^{13}C (125.64 MHz) chemical shifts of euthailol A (**1**) in CD_3OD at 303K

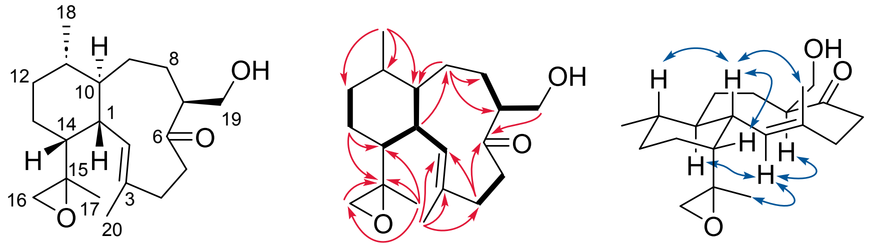

1 — COSY, key HMBC, key NOESY

no.	δ_C, type	δ_H, mult. (J in Hz)
1	46.3, CH	2.13, td (11.4, 6.1)
2	131.7, CH	5.57, d (11.4)
3	134.8, C	
4	34.8, CH_2	2.66, m
		2.32, m
5	42.9, CH_2	3.11, m
		2.36, m
6	219.2, C	
7	55.4, CH	3.26, m
8	30.3, CH_2	1.85, m
		1.27[a]
9	30.6, CH_2	1.30, m
		0.78, m
10	47.0, CH	0.93, m
11	39.4, CH	1.15, m
12	32.3, CH_2	1.55, m
		1.26[a]
13	28.2, CH_2	1.77, m
		1.61, m
14	44.8, CH	1.51, td (6.1, 2.3)
15	60.7, C	
16	54.3, CH_2	2.57, d (4.7)
		2.50, d (4.7)
17	24.9, CH_3	1.47, s
18	21.0, CH_3	0.97, d (6.3)
19	65.8, CH_2	3.51, dd (11.0, 7.9)
		3.46, dd (11.0, 5.9)
20	18.6, CH_3	1.57, s

[a]Overlapping signals.

Table S2. ^{1}H (499.63 MHz) and ^{13}C (125.64 MHz) chemical shifts of euthailol B (**2**) in CD_3OD at 303K

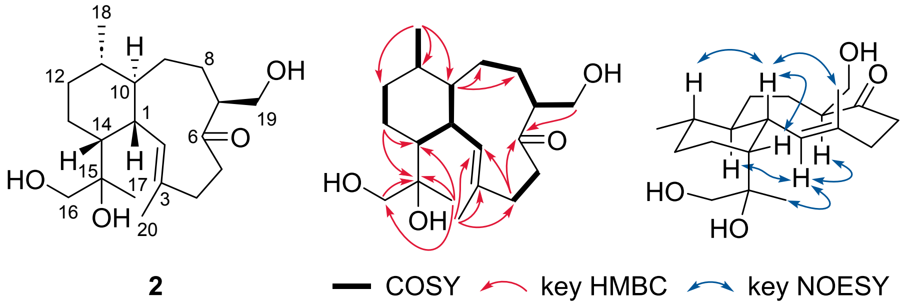

2

no.	δ_C, type	δ_H, mult. (J in Hz)
1	46.2, CH	2.14, td (11.2, 6.7)
2	131.4, CH	5.47, d (6.7)
3	135.5, C	
4	34.7, CH_2	2.61, m
		2.32, m
5	42.8, CH_2	3.12, m
		2.35, m
6	219.2, C	
7	55.4, CH	3.26, m
8	30.3, CH_2	1.83, m
		1.29[a]
9	30.5, CH_2	1.32, m
		0.74, m
10	47.0, CH	0.91, tt (11.2, 3.0)
11	39.7, CH	1.13, m
12	32.7, CH_2	1.57[b]
		1.30[a]
13	28.1, CH_2	1.96, m
		1.60, m
14	44.8, CH	1.40, br t (6.1)
15	60.5, C	
16	57.9, CH_2	2.57, s
17	23.9, CH_3	1.45, s
18	21.0, CH_3	0.96, d (6.3)
19	65.7, CH_2	3.51, dd (10.5, 8.0)
		3.46, dd (10.5, 5.9)
20	18.6, CH_3	1.57, s

[a]Overlapping signals. [b]Overlapping with a signal for H_3-20

Table S3. ^{1}H (499.63 MHz) and ^{13}C (125.64 MHz) chemical shifts of euthailol C (**3**) in CD_3OD at 303K

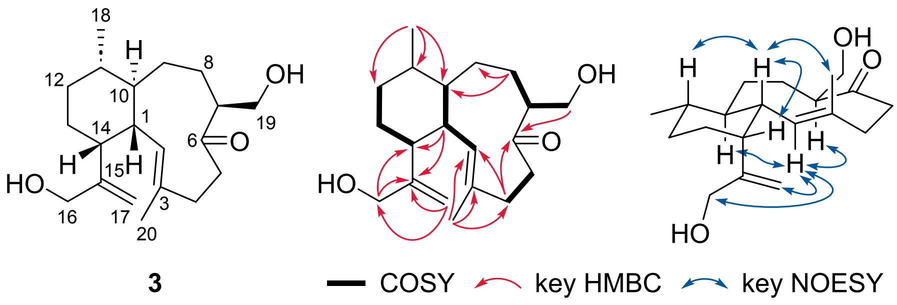

no.	δ_C, type	δ_H, mult. (J in Hz)
1	46.9, CH	2.10, td (11.0, 5.5)
2	131.9, CH	5.44, d (11.0)
3	134.8, CH	
4	34.7, CH_2	2.56, m
		2.28, m
5	42.8, CH_2	3.05, m
		2.31, m
6	219.4, C	
7	55.4, CH	3.21, m
8	30.5, CH_2	1.82, m
		1.26[a]
9	31.0, CH_2	1.27[a]
		0.78, m
10	46.5, CH	1.02, m
11	40.2, CH	1.14, m
12	31.0, CH_2	1.47, m
		1.39, m
13	31.4, CH_2	1.65, m
14	40.8, CH	2.20, m
15	151.9, C	
16	67.7, CH_2	3.85, m
17	111.0, CH_2	5.30, s
		5.18, br s
18	21.0, CH_3	0.98, d (6.3)
19	65.9, CH_2	3.49, dd (10.8, 8.9)
		3.43, dd (10.8, 5.9)
20	18.7, CH_3	1.57, d (0.8)

[a]Overlapping signals.

Tabele S4. ^{1}H (500.18 MHz) and ^{13}C (125.78 MHz) chemical shifts of euthailol D (**4**) in C_5D_5N at 298K

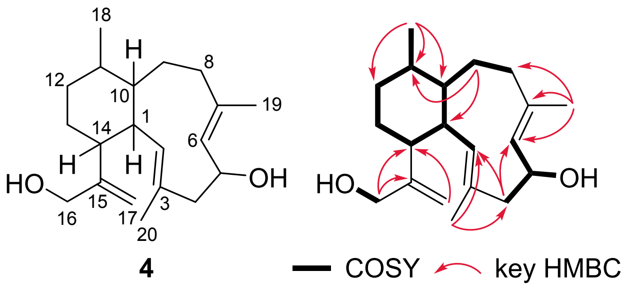

4

no.	δ_C, type	δ_H, mult. (J in Hz)
1	46.4, CH	2.14, m
2	137.5, CH	5.10, d (9.2)
3	128.8, C	
4	50.3, CH_2	2.71, dd (11.3, 5.5)
		2.27, br t (11.3)
5	68.8, CH	4.91[a]
6	132.2, CH	5.25, d (9.8)
7	137.1, C	
8	41.4, CH_2	2.34, m
		2.01, m
9	34.6, CH_2	1.88, dd (15.1, 9.2)
		1.04[b]
10	49.9, CH	1.26, m[c]
11	37.2, CH	1.11, m
12	29.8, CH_2	1.44, m
		1.25, m[c]
13	28.9, CH_2	1.66, m
		1.53, m[d]
14	41.4, CH	2.44, br s
15	151.9, C	
16	66.7, CH_2	4.23, br s
17	109.9, CH_2	5.70, s
		5.17, s
18	21.8, CH_3	
19	17.4, CH_3	1.50, s
20	17.6, CH_3	1.56, s
OH-5		6.19, br s
OH-17		6.43, dd (11.6, 5.6)

[a]Overlapping with H_2O signal. [b]Overlapping with a signal for H_3-18. [c]Overlapping signals. [d]Overlapping with a signal for H_3-19.

Tabel S5. ^{1}H and ^{13}C NMR chemical shifts of euthailol E (**5**) at 298K

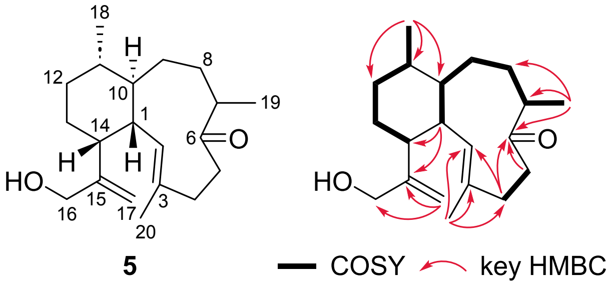

no.	δ_C, type[a]	δ_H, mult. (J in Hz)[a]	δ_H, mult. (J in Hz)[b]
1	46.8, CH	2.11, dd (10.9, 5.5)	2.07, td (11.0, 5.5)
2	131.6, CH	5.40, br d (10.9)	5.29, d (11.0)
3	134.0, C		
4	34.1, CH_2	2.49, m	2.49, m
		2.24, dd (14.8, 7.1)	2.30, dd (14.4, 6.9)
5	39.8, CH_2	3.00, m	2.94, m
		2.21[c]	2.26, m
6	215.9, C		
7	46.0, CH	2.91[d]	2.85, m
8	35.0, CH_2	1.83, m	1.87, m
		1.32, m	1.27[c]
9	30.4, CH_2	1.25, m	1.27[c]
		0.72, ddt (14.5, 10.2, 4.3)	0.71, m
10	46.3, CH	1.03, m	0.93, m
11	39.7, CH	1.12, m	1.13, m
12	31.1, CH_2	1.45, ddd (13.7, 7.7, 3.8)	1.48, ddd (13.8, 7.5, 3.7)
		1.39, m	1.34, m
13	30.7, CH_2	1.63[c]	1.62, m
		1.61[c]	1.61, m
14	40.5, CH	2.18[c]	2.24, br dd (7.9, 5.1)
15	152.0, C		
16	67.3, CH_2	3.86, br s	3.96, s
17	110.3, CH_2	5.29, d (1.0)	5.27, s
		5.12, s	5.17, s
18	21.0, CH_3	0.96, d (6.3)	0.951, d (6.3)
19	17.5, CH_3	0.90, d (6.9)	0.954, d (7.0)
20	18.9, CH_3	1.55, d (1.3)	1.58, d (1.1)

[a]Recorded in $(CD_3)_2CO$ at 500.18 MHz for ^{1}H and 125.78 MHz for ^{13}C. [b]Recorded in $CDCl_3$ at 600.21 MHz for ^{1}H and 150.94 MHz for ^{13}C. [c]Overlapping signals. [d]Overlapping with a signal for H_2O.

Table S6. ^{1}H (600.21 MHz) and ^{13}C (150.94 MHz) NMR chemical shifts of euthailol F (**6a**) and G (**6b**) in $(CD_3)_2CO$ at 298K

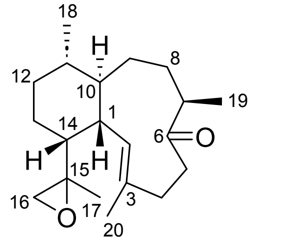

6a and **6b**
15*R* or 15*S* diastereomers
(not clarified)

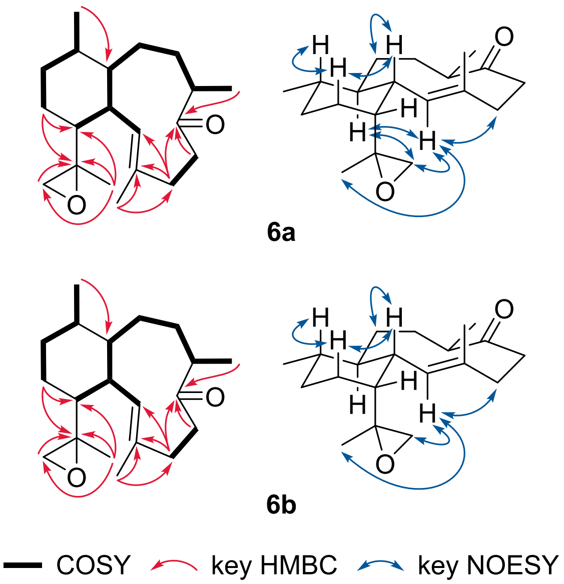

	**6a**		**6b**	
no.	δ_C, type	δ_H, mult. (*J* in Hz)	δ_C, type[a]	δ_H, mult. (*J* in Hz)
1	46.2, CH	2.13, ddd (11.4, 11.4, 5.7)	46.0, CH	2.14, ddd (11.3, 11.3, 6.6)
2	131.7, CH	5.57, dq (11.4, 1.5)	131.3, CH	5.50, dq (11.3, 1.4)
3	133.7, C		134.4, C	
4	34.2, CH_2	2.58, m	33.9, CH_2	2.57, m
		2.27, m		2.27, m
5	39.8, CH_2	3.07, m	39.6, CH_2	3.09, ddd (13.4, 8.3, 6.9)
		2.24, m		2.22, m
6	215.7, C		215.6, C	
7	45.9, CH	2.99, m	45.8, CH	2.99, m
8	34.9, CH_2	1.84, dddd (14.7, 10.1, 4.3, 4.3)	34.7, CH_2	1.84, dddd (13.7, 10.5, 6.4, 4.3)
		1.34, dddd (13.8, 10.6, 6.3, 4.3)		1.36[b]
9	30.1, CH_2	1.28, m	30.2, CH_2	1.29[b]
		0.71, dddd (14.7, 10.1, 4.3, 4.3)		0.69, dddd (14.7, 9.9, 4.3, 4.3)
10	46.5, CH	0.96, m	46.4, CH	0.94, m
11	38.7, CH	1.14, m	38.8, CH	1.11, m
12	32.0, CH_2	1.52, m	32.3, CH_2	1.54, m
		1.23, m		1.29[b]
13	27.8, CH_2	1.76, m	27.8, CH_2	1.94, m
		1.56[b]		1.57, m
14	43.8, CH	1.55[b]	44.3, CH	1.36[b]
15	58.9, C		58.9, C	
16	53.2, CH_2	2.52, dd (5.1, 0.7)	56.1, CH_2	2.47, dd (5.2, 0.5)
		2.38, d (5.1)		2.45, d (5.2)
17	25.1, CH_3	1.38, d (0.7)	23.8, CH_3	1.39, s
18	20.9, CH_3	0.94, d (6.3)	20.7, CH_3	0.95, d (6.4)
19	17.4, CH_3	0.92, d (7.0)	17.2, CH_3	0.92, d (6.9)
20	18.7, CH_3	1.55, d (1.5)	18.6, CH_3	1.56, d (1.4)

[a]assigned by HSQC and HMBC correlations. [b]Overlapping signals.

Table S7. ^{1}H (600.21 MHz) and ^{13}C (150.94 MHz) NMR chemical shifts of euthailol H (**7**, albireticulone A) in CD_3OD at 298K

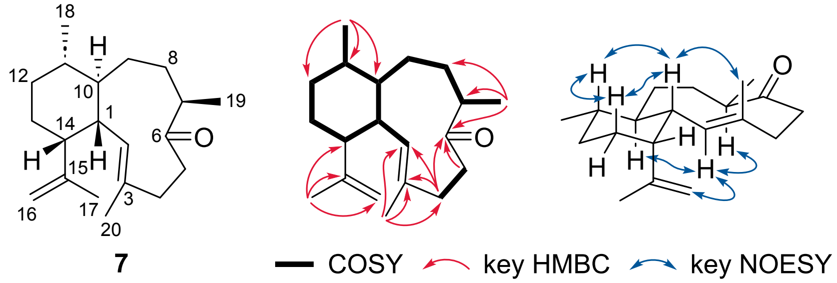

7

	**7**			albireticulone A[a]
no.	δ_C, type[b]	δ_H, mult. (J in Hz)[b]	δ_H, mult. (J in Hz)[c]	δ_H, mult. (J in Hz)
1	47.5, CH	2.10, td (11.1, 5.5)	2.06, td (11.1, 5.5)	2.05, td (11.1, 1.4)
2	132.2, CH	5.47, br d (11.1)	5.41, dt (11.1, 1.3)	5.40, d (11.1)
3	134.4, C			
4	34.4, CH_2	2.53, m	2.52, m	2.52, m
		2.27[d]	2.29, m	2.29, m
5	40.4, CH_2	3.05, m	2.96, m	2.96, m
		2.26[d]	2.28, m	2.28, m
6	219.9, C			
7	47.1, CH	2.95, m	2.85, m	2.85, ddd (10.1, 6.8, 2.6)
8	35.3, CH_2	1.84, m	1.86, m	1.85, ddt (12.7, 9.2, 5.3)
		1.34, m	1.27[d]	1.27, m
9	30.7, CH_2	1.31, m	1.27[d]	1.26, m
		0.71, ddt (14.5, 10.2, 4.3)	0.70, m	0.69, td (11.7, 7.2)
10	46.7, CH	1.02, br tdd (11.1, 3.9, 2.4)	0.98, m	0.96, m
11	40.2, CH	1.12, m	1.11, m	1.10, tq (10.9, 6.3, 4.4)
12	31.5, CH_2	1.46, m	1.45, m	1.44, m
		1.36, m	1.32, m	1.31, m
13	30.8, CH_2	1.71, m	1.70, m	1.69, m
		1.61, m	1.56, m	1.55, m
14	45.7, CH	2.26[d]	2.25, m	2.25, m
15	148.5, C			
16	113.2, CH_2	4.94, s	4.93, br quint (1.6)	4.92, s
		4.87, s	4.86, br t (0.8)	4.85, s
17	26.8, CH_3	1.74, br s	1.74, br dd (1.3, 0.8)	1.74, s
18	21.0, CH_3	0.949, d (6.3)	0.93, d (6.4)	0.92, d (6.4)
19	17.5, CH_3	0.946, d (7.0)	0.96, d (7.0)	0.95, d (7.0)
20	18.9, CH_3	1.57, d (1.1)	1.59, d (1.3)	1.58, s

[a]Previously reported in $CDCl_3$ (600 MHz).[1] [b]Recorded in CD_3OD. [c]Recorded in $CDCl_3$. [d]Overlapping signals.

Table S8: Antibacterial effect of euthailols **4, 5, 6a, 6b, 7** (albireticulone A)**,** trimethoprim (Tmp.) and ampicillin (Amp.) against ESKAPE pathogens. The value >256 µg/mL represents no bioactivity observed.

**Bacteria**	**Compounds MIC µg/mL**					**Antibiotics**	
	**4**	**5**	**6a**	**6b**	**7**	**Tmp.**	**Amp.**
Acinetobacter baumanni	64	128	>256	>256	>256	128	16
Enterobacter cloacae	128	>256	>256	>256	>256	8	>256
Pseudomonas aeruginosa	32	64	64	64	64	4	32
Klebsiella pneumoniae	>256	>256	>256	>256	>256	>256	64
Staphylococcus aureus	128	>256	>256	>256	>256	128	64
Enterococcus faecium	>256	>256	>256	>256	>256	64	32
Arthobacter pascens	16	32	>256	>256	16	8	2

Table S9: Strains used in this study

Strains	Genotype/Description	Sources
E. coli DH5α	*fhuA2 lac(del)U169 phoA glnV44 Φ80' lacZ(del)M15 gyrA96 recA1 relA1 endA1 thi-1 hsdR17*	Lab stock
E. coli BL21 Star(DE3)	F–*ompT hsd*SB (rB–, mB–) *gal dcm rne*131 (DE3)	Lab stock
E. coli BL21(DE3) MEV20	F–*ompT hsdSB* (*rB*–, *mB*–) *gal dcm (DE3) ERG12 ERG8 MVD1 idi ggps atoB HMGS tHMGR fdxD fprD fldA fdr fer1 fenr pdx pdr*	Kindly provided by Prof. Christopher A. Voigt, Synthetic Biology Center, Department of Biological Engineering, Massachusetts Institute of Technology, Cambridge, MA, USA[2]
E. coli ET12567 (pUZ8002)	F- *dam*-13::Tn9 *dcm*-6 *hsdM hsdR zjj*-202::Tn10 *recF*143 *galK2 galT22 ara*-14 *lacY*1 *xyl*-5 *leuB6 thi*-1 *tonA*31 *rpsL*136 *his*G4 *tsx*-78 *mtl*-1 *glnV*44	Kindly provided by Prof. Dr. Helge B. Bode, Institute for Molecular Bioscience, Goethe University Frankfurt, Germany
Streptomyces albus J1074	*S. albus* G derivative with the defective SalI-restriction activity	Kindly provided by PD Dr. Bertolt Gust, Department of Pharmceutical Biology, Pharmaceutical Institute, Eberhard Karls University Tübingen, Germany
Streptomyces coelicolor M1154	M145 derivative ∆*act*, ∆*red* ∆*cda*, ∆*cpk rpoB*[*C1298T*], *rpsL*[*A262G*]	Kindly provided by PD Dr. Bertolt Gust, Department of Pharmceutical Biology, Pharmaceutical Institute, Eberhard Karls University Tübingen, Germany
Streptomyces coelicolor M1154	*S. coelicolor* M1154 harboring pIJ10257_MVA and pSET152*_*eut* BGC (full cluster)	This work
Streptomyces avermitilis SUKA22	*S. avermitilis* SUKA22 harboring pIJ10257_MVA	This work
Streptomyces avermitilis SUKA22	*S. avermitilis* SUKA22 harboring pIJ10257_MVA and pSET152*_*eut* BGC (full cluster)	This work
Streptomyces albus AKH001	*S. albus* J1074 harboring pIJ10257_MVA	This work

Streptomyces albus AKH002	*S. albus* J1074 harboring pIJ10257_MVA and empty pSET152*	This work
Streptomyces albus AKH003	*S. albus* J1074 harboring pIJ10257_MVA and pSET152*_*eutAB*	This work
Streptomyces albus AKH004	*S. albus* J1074 harboring pIJ10257_MVA and pSET152*_*eut* BGC (full cluster)	This work
Streptomyces albus AKH005	*S. albus* J1074 harboring pIJ10257_MVA and pSET152*_*eutABEFG*	This work
Streptomyces albus AKH006	*S. albus* J1074 harboring pIJ10257_MVA and pSET152*_*eutABD*	This work
Streptomyces albus AKH007	*S. albus* J1074 harboring pIJ10257_MVA and pSET152*_*eutABDEFG*	This work
Streptomyces albus AKH008	*S. albus* J1074 harboring pIJ10257_MVA and pSET152*_*eutABC*	This work
Streptomyces albus AKH0098	*S. albus* J1074 harboring pIJ10257_MVA and pSET152*_*eutABCD*	This work
Streptomyces albus AKH009	*S. albus* J1074 harboring pIJ10257_MVA and pSET152*_*eutABC*	This work
Streptomyces albus AKH010	*S. albus* J1074 harboring pIJ10257_MVA and pSET152*_*eutABCEFG*	This work
E. coli BL21 (DE3) MEV20 AKH011	*E. coli* BL21 (DE3) MEV20 harboring pSC101 empty plasmid	This work
E. coli BL21 (DE3) MEV20 AKH012	*E. coli* BL21 (DE3) MEV20 harboring pSC101_*eutB*	This work
E. coli BL21 (DE3) MEV20 AKH013	*E. coli* BL21 (DE3) MEV20 harboring pSC101_*eutB* ΔHMBPPR	This work
E. coli BL21 (DE3) MEV20 AKH014	*E. coli* BL21 (DE3) MEV20 harboring pSC101_*eutB* ΔNxxxSxxxE motif	This work
E. coli BL21 (DE3) MEV20 AKH015	*E. coli* BL21 (DE3) MEV20 harboring pSC101_*eutB* ΔWxxxxxRY motif	This work
E. coli BL21 (DE3) MEV20 AKH016	*E. coli* BL21 (DE3) MEV20 harboring pSC101_*eutB* ΔNxxxSxxxE ΔWxxxxxRY motif	This work
Staphylococcus aureus DSM 11823	*Staphylococcus aureus* Rosenbach 1884	DSMZ
Streptococcus mutans DSM 20523	*Streptococcus mutans* Clarke 1924	DSMZ

Table S10: Plasmids used in this study

Plasmids	Description	Sources
pHL01	pSC101 empty plasmid	This study
pHL02	pSC101 based plasmid harboring *eutB* gene, encoding full length terpene cyclase	This study
pHL03	pSC101 based plasmid harboring truncated terpene cyclase without HMBPPR domain	This study
pHL04	pSC101 based plasmid harboring truncated terpene cyclase without cyclase domain	This study
pHL05	pSC101 based plasmid harboring truncated terpene cyclase without NxxxSxxxE motif	This study
pHL06	pSC101 based plasmid harboring truncated terpene cyclase without WxxxxxRY motif	This study
pHL07	pSC101 based plasmid harboring truncated terpene cyclase without NxxxSxxxE and WxxxxxRY motif	This study
pHL08	*E. coli/Streptomyces* shuttle vector pSET152 with ermE promoter	This study
pHL09	Integrative plasmid pSET152* harboring *eutAB*	This study
pHL10	Integrative plasmid pSET152* harboring *eutABCDEFG*	This study
pHL11	Integrative plasmid pSET152* harboring *eutABCEFG*	This study
pHL12	Integrative plasmid pSET152* harboring *eutABEFG*	This study
pHL13	Integrative plasmid pSET152* harboring *eutABCDEFG* (△reductase)	This study
pHL14	Integrative plasmid pSET152* harboring *eutABCD*	This study
pHL15	Integrative plasmid pSET152* harboring *eutABC*	This study
pHL16	Integrative plasmid pSET152* harboring *eutABD*	This study
pIJ10257	empty plasmid	Kindly provided by PD Dr. Bertolt Gust, Department of Pharmaceutical Biology, Pharmaceutical Institute, Eberhard Karls University
pIJ10257_MVA	Integrative plasmid pJI10257 harboring MVA pathway from *Streptomyces* sp. CL190	This study

Table S11: Primers used in this study

Primer	Sequence	Construct
ST001	ggaggttaatTAATTAAATCATCCCTATGACCC	pHL01
ST002	ggatgatttaATTAACCTCCTTAAAACTGATC	
ST003	atgcaggcttCCTCGCTCACTGACTCGC	pHL02
ST004	ccgcgaattcCAGAAATCATCCTTAGCGAAAGC	
ST005	atgatttctgGAATTCGCGGCCGCTTCTAG	
ST006	gtgagcgaggAAGCCTGCATAACGCGAAG	
ST007	ccaagagatcTAAATCATCCCTATGACCC	pHL03
ST008	aaggaagagtATGAGTATTCAACATTTCCG	
ST009	gaatactcatACTCTTCCTTTTTCAATATTATTGAAGC	
ST010	ggatgatttaGATCTCTTGGCTCGGCTG	
ST011	ttaaaaatgaaGTTTTAAATCAATCTAAAGTATATATGAG	pHL04
ST012	taccacgcatATTAACCTCCTTAAAACTGATC	
ST013	ggaggttaatATGCGTGGTATTCCGATG	
ST014	gatttaaaacTTCATTTTTAATTTAAAAGGATCTAGGTG	
ST015	gctgctgtatACCCTCGCAGGCCGGTCT	pHL05
ST016	ctgcgagggtATACAGCAGCGCATAATCTGAACTGC	
ST017	cgtgaaccacCTGGTTGGACAGCCGCTCG	pHL06
ST018	gtccaaccagGTGGTTCACGCCCCCGGC	
ST019	ggcatgcaagctAGCTTGGATCCTAGGTTC	pHL07
ST020	gtacgactctagAGCTTAGATCTATGCAGG	
ST021	atagatctaagctCTAGAGTCGTACTTCCCTGGC	
ST022	taggatccaagctAGCTTGCATGCCTGCAGG	
ST023	gaggccccatGTGCCAAGCTTGGGCTGC	pHL08
ST024	gtcgggctggCCGATGCAAAGTGCCGATC	
ST025	tttgcatcggCCAGCCCGACCCGAGCAC	
ST026	agcttggcacATGGGGCCTCCTGTTCTATCCTACC	
ST027	cgccgcttgaCGTAATCATGTCATAGCTGTTTCCTG	pHL09
ST028	tcgtggtcatATGGGGCCTCCTGTCCTAG	
ST029	gaggccccatATGACCACGACGACCCCAC	

ST030	catgattacgTCAAGCGGCGGGTTCTCG	
ST031	gccacaatgaCGTAATCATGTCATAGCTGTTTCCTG	pHL10
ST032	tcgtggtcatATGGGGCCTCCTGTCCTAG	
ST033	gaggccccatATGACCACGACGACCCCAC	
ST034	catgattacgTCATTGTGGCGCTGACGG	
ST035	gctcatgacgCGGCTCATCCGCCCTCGT	pHL11
ST036	ggatgagccgCGTCATGAGCCCTTGCAGTC	
ST043	gtggtggccgAGTCCATTCGCGCGCCGC	pHL12
ST044	cgaatggactCGGCCACCACCTTCGTGTTC	
ST045	ggcgtcgaccATGAGGGGCATCCCGATGGGTTC	pHL13
ST046	tgcccctcatGGTCGACGCCCGCGCACC	
ST047	gagtggcccaAGCTGTTTCCTGTGTGAAATTGTTATCC	pHL14
ST048	ggaaacagctTGGGCCACTCCCAAGAGG	
ST049	aggctcctgaTGCCTAATGAGTGAGCTAACTCAC	pHL15
ST050	cgaatggactTCAAGCGGCGGGTTCTCG	
ST051	cgccgcttgaAGTCCATTCGCGCGCCGC	
ST052	tcattaggcaTCAGGAGCCTTTGCACAGGTCC	
ST053	gcaagggctcCTGTGTGAAATTGTTATCCGC	pHL16
ST054	tttcacacagGAGCCCTTGCAGTCGAAG	

Table S12. Comparison of experimental and calculated ^{13}C NMR chemical shifts for euthailol A (**1**).

	δ_C exp[a]	δ_C calc (mPW1PW91)[b]		δ_C calc (B3LYP)[c]	
no.	**1**	(15*R*)-**1**	(15*S*)-**1**	(15*R*)-**1**	(15*S*)-**1**
1	46.3	48.71909	48.67276	49.56217	49.31888
2	131.7	133.59922	133.25237	130.88568	130.79672
3	134.8	142.97936	144.25676	141.16223	142.55541
4	34.8	32.33367	32.07006	32.23751	32.15632
5	42.9	42.35281	42.12377	41.49688	41.39334
6	219.2	225.01055	224.55286	224.21591	223.40032
7	55.4	57.00852	56.96722	57.46252	57.09014
8	30.3	32.51155	32.58786	31.47201	31.47049
9	30.6	28.33222	28.49529	27.45692	27.79939
10	47.0	48.07746	48.18089	48.62538	48.98417
11	39.4	38.96745	38.91688	38.37680	38.25134
12	32.3	32.56979	33.29515	32.48448	32.85087
13	28.2	29.09132	27.77744	28.90369	27.52203
14	44.8	46.84755	47.38000	47.66687	47.97546
15	60.7	59.66624	58.50549	59.60426	58.49917
16	54.3	53.08666	56.77587	52.97617	56.23515
17	24.9	23.76596	22.24050	22.94710	21.70142
18	21.0	19.51936	19.45791	19.51648	19.48067
19	65.8	63.48627	63.83545	63.20350	63.34937
20	18.6	20.11753	20.20765	19.45401	19.21491
MAE[d]		2.04	2.32	2.08	2.26
MSE[e]		2.73	3.02	2.55	2.76
DP4[f]		95.378	4.622	90.204	9.796

[a]Recorded in CD_3OD at 125.64 MHz (303K). [b]Calculated at mPW1PW91/6-311+G(2d,p)//B3LYP/6-31G(d,p). [c]Calculated at B3LYP/6-31G(d,p)//B3LYP/6-31G(d,p). [d]Mean absolute error (ppm). [e]Mean squared error. [f]DP4 probability (%).

Table S13. Comparison of experimental and calculated ^{1}H NMR chemical shifts for euthailol A (**1**).

	δ_H exp[a]	δ_H calc (mPW1PW91)[b]		δ_H calc (B3LYP)[c]	
no.	**1**	(15*R*)-**1**	(15*S*)-**1**	(15*R*)-**1**	(15*S*)-**1**
1	2.13	2.197324	2.196509	2.290923	2.282359
2	5.57	5.730657	5.518613	6.034439	5.725855
4	2.32	2.455467	2.366536	2.410766	2.262312
4	2.66	2.458828	2.451720	2.427782	2.421424
5	2.36	2.333916	2.168440	2.105480	2.105346
5	3.11	3.124631	3.246574	3.206078	3.160708
7	3.26	2.595862	2.600584	2.586656	2.626729
8	1.27	1.823987	1.736770	1.775318	1.763258
8	1.85	1.909991	1.951595	1.964630	1.876779
9	0.78	1.028615	0.669659	0.681783	0.672865
9	1.30	1.068704	1.461636	1.404427	1.401447
10	0.93	0.984668	0.893201	1.253067	1.113012
11	1.15	1.143678	1.149434	1.269620	1.264707
12	1.26	1.262865	1.337122	1.479326	1.505203
12	1.55	1.533161	1.590831	1.530927	1.570702
13	1.61	1.621515	1.567892	1.701033	1.616113
13	1.77	1.845776	2.163588	1.771821	2.200595
14	1.51	1.341226	1.226207	1.201564	1.154104
16	2.5	2.255363	2.280009	2.252404	2.369354
16	2.57	2.303546	2.321725	2.273019	2.389408
17	1.47	1.390761	1.381033	1.490578	1.439828
18	0.97	0.986927	0.992960	0.996923	0.998624
19	3.46	3.613339	3.580307	3.310519	3.327853
19	3.51	3.616365	3.665567	4.036446	4.005862
20	1.57	1.716314	1.725742	1.708754	1.718718
MAE[d]		0.149	0.163	0.211	0.191
MSE[e]		0.218	0.222	0.273	0.253
DP4[f]		94.469	5.531	7.447	92.553

[a]Recorded in CD_3OD at 499.63 MHz (303K). [b]Calculated at mPW1PW91/6-311+G(2d,p)//B3LYP/6-31G(d,p). [c]Calculated at B3LYP/6-31G(d,p)//B3LYP/6-31G(d,p). [d]Mean absolute error (ppm). [e]Mean squared error. [f]DP4 probability (%).

Table S14. Comparison of experimental and calculated ^{13}C NMR chemical shifts for euthailol F (**6a**) and G (**6b**).

	δ_C exp[a]		δ_C calc (mPW1PW91)[b]				δ_C calc (B3LYP)[c]			
no.	**6a**	**6b**	(7*R*,15*R*)-**6**	(7*R*,15*S*)-**6**	(7*S*,15*R*)-**6**	(7*S*,15*S*)-**6**	(7*R*,15*R*)-**6**	(7*R*,15*S*)-**6**	(7*S*,15*R*)-**6**	(7*S*,15*S*)-**6**
1	46.2	46	46.57130	46.39308	47.40337	47.69345	47.14025	46.92163	47.91294	48.09097
2	131.7	131.3	135.59832	138.46472	136.47326	136.20684	133.57986	136.93933	134.42880	134.23872
3	133.7	134.4	139.65484	137.83620	138.92492	139.69167	137.54156	135.32197	136.99083	137.68346
4	34.2	33.9	35.80698	38.31828	38.34114	38.01983	35.67420	37.90610	38.45954	38.10893
5	39.8	39.6	40.73372	40.78012	43.07876	43.29148	40.08221	39.87917	42.88511	43.16292
6	215.7	215.6	220.08922	218.86243	223.70187	224.13575	219.62834	218.22501	223.73877	224.27452
7	45.9	45.8	47.81690	44.79987	53.22466	53.21138	47.29370	43.99727	53.39198	53.33622
8	34.9	34.7	35.48669	35.23952	36.14155	36.92017	35.81835	35.81834	36.91902	37.80477
9	30.1	30.2	30.95416	31.57056	31.38153	31.51020	31.09385	31.71331	31.98018	32.06969
10	46.5	46.4	46.10323	47.39907	50.05089	50.52214	46.46267	47.94088	50.93827	51.46291
11	38.7	38.8	40.59854	40.31333	40.10529	40.08079	40.41579	40.19165	39.80018	39.69847
12	32	32.3	31.65920	31.95224	31.66837	32.63604	31.37333	31.55982	31.41355	32.26223
13	27.8	27.8	28.63557	28.47842	28.89864	28.25320	28.24520	28.28953	28.50868	28.04192
14	43.8	44.3	45.69365	44.78501	45.26048	46.38034	45.93154	45.10028	45.52040	46.80531
15	58.9	58.9	59.78527	60.05367	59.68140	59.33175	59.45975	60.04031	59.47461	59.31588
16	53.2	56.1	52.81532	54.85547	52.77127	56.22134	52.51297	54.23005	52.40420	55.47313
17	25.1	23.8	24.54989	23.35652	24.43305	22.42925	24.26689	22.91998	24.13898	21.95916
18	20.9	20.7	19.63642	19.61723	19.67990	19.68817	19.59061	19.64019	19.71363	19.68460
19	17.4	17.2	18.27345	18.63233	18.49894	18.70911	18.30598	18.66953	18.51316	18.75242
20	18.7	18.6	17.56845	14.61784	15.40655	15.44148	16.69329	13.45415	14.61237	14.60969
MAE (**6a**)[d]			1.55	1.88	2.59	2.97	1.35	1.78	2.59	2.93
MAE (**6b**)[e]			1.71	1.81	2.75	2.75	1.49	1.77	2.73	2.77
MSE (**6a**)[f]			2.14	2.51	3.41	3.68	1.68	2.26	3.34	3.63
MSE (**6b**)[g]			2.22	2.49	3.52	3.60	1.82	2.28	3.47	3.57
DP4 (**6a**)[h]			98.183	1.173	0.516	0.129	99.752	0.236	0.009	0.002
DP4 (**6b**)[i]			95.339	3.637	0.268	0.756	99.738	0.257	0.002	0.003

[a]Recorded in $(CD_3)_2CO$ at 150.94 MHz (298K). [b]Calculated at mPW1PW91/6-311+G(2d,p)//B3LYP/6-31G(d,p). [c]Calculated at B3LYP/6-31G(d,p)//B3LYP/6-31G(d,p). [d]Mean absolute error (ppm) against experimental ^{13}C chemical shifts of **6a**. [e]Mean absolute error (ppm) against experimental ^{13}C chemical shifts of **6b**. [f]Mean squared error against experimental ^{13}C chemical shifts of **6a**. [g]Mean squared error against experimental ^{13}C chemical shifts of **6b**. [h]DP4 probability (%) for **6a**. [i]DP4 probability (%) for **6b**

Table S15. Comparison of experimental and calculated ^{1}H NMR chemical shifts for **6a** and **6b.**

	δ_H exp[a]		δ_H calc (mPW1PW91)[b]				δ_H calc (B3LYP)[c]			
no.	**6a**	**6b**	(7*R*,15*R*)-**6**	(7*R*,15*S*)-**6**	(7*S*,15*R*)-**6**	(7*S*,15*S*)-**6**	(7*R*,15*R*)-**6**	(7*R*,15*S*)-**6**	(7*S*,15*R*)-**6**	(7*S*,15*S*)-**6**
1	2.13	2.14	2.079349	2.056393	2.028768	2.046258	2.167254	2.136559	2.135084	2.161493
2	5.57	5.5	5.603381	5.444216	5.353614	5.286357	5.886364	5.686547	5.626559	5.532907
4	2.27	2.27	2.454406	2.415163	2.060747	2.025885	2.412895	2.376271	2.033841	1.979919
4	2.58	2.57	2.608053	2.711588	3.012985	2.993077	2.523454	2.547915	2.998518	2.953392
5	2.24	2.22	2.322998	2.522897	2.281314	2.278481	2.523454	2.408107	2.240860	2.238515
5	3.07	3.09	2.978986	2.769360	2.919934	2.920122	2.925971	2.786922	2.846217	2.843253
7	2.99	2.99	2.996615	3.097407	2.562184	2.573651	3.062620	3.187058	2.533552	2.542089
8	1.34	1.36	1.028190	1.046171	1.504152	1.534170	1.106880	1.147899	1.571495	1.595501
8	1.84	1.84	2.068636	2.066670	1.712432	1.722958	2.193261	2.209881	1.866105	1.881662
9	0.71	0.69	1.008959	0.893616	0.874998	0.837915	1.262253	1.121742	1.089261	1.060743
9	1.28	0.94	1.192482	1.290128	1.370110	1.400588	1.328565	1.432383	1.519650	1.563479
10	0.96	0.94	0.886700	0.912243	0.986966	1.011375	1.191962	1.206706	1.193998	1.254491
11	1.14	1.11	1.116556	1.089089	1.017462	1.035870	1.269792	1.249592	1.165519	1.182993
12	1.23	1.29	1.182880	1.203071	1.181279	1.294381	1.363172	1.385292	1.366029	1.469929
12	1.52	1.54	1.457031	1.467836	1.418420	1.507846	1.458550	1.470581	1.410927	1.497617
13	1.56	1.57	1.539521	1.524923	1.462040	1.492887	1.580681	1.580650	1.498525	1.558939
13	1.76	1.94	1.900341	2.008438	1.936814	2.089314	1.928366	2.030572	1.965165	2.125900
14	1.55	1.26	1.511562	1.430106	1.562780	1.269339	1.498639	1.398592	1.543444	1.229893
16	2.38	2.47	2.284213	2.257168	2.291106	2.299211	2.364813	2.335025	2.390813	2.372890
16	2.52	2.45	2.419322	2.471021	2.477317	2.309701	2.548676	2.560514	2.625569	2.392397
17	1.38	1.39	1.345713	1.317452	1.278856	1.319632	1.424049	1.397724	1.348329	1.416232
18	0.94	0.95	0.981463	0.981620	0.946926	0.962156	1.038197	1.045504	0.985222	1.004366
19	0.92	0.92	0.989556	0.995635	0.914198	0.922321	1.026566	1.038850	0.961721	0.970900
20	1.55	1.56	1.608147	1.652392	1.773459	1.759607	1.616300	1.653828	1.769767	1.752660
MAE (**6a**)[d]			0.092	0.122	0.133	0.153	0.142	0.145	0.146	0.171
MAE (**6b**)[e]			0.113	0.135	0.157	0.147	0.167	0.162	0.175	0.168
MSE (**6a**)[f]			0.123	0.150	0.172	0.194	0.190	0.180	0.198	0.223
MSE (**6b**)[g]			0.149	0.169	0.202	0.195	0.220	0.206	0.235	0.233
DP4 (**6a**)[h]			98.6183	1.0001	0.3613	0.0203	54.248	45.440	0.307	0.005
DP4 (**6b**)[i]			97.052	2.533	0.022	0.393	14.384	85.511	0.020	0.085

[a]Recorded in $(CD_3)_2CO$ at 600.21 MHz (298K). [b]Calculated at mPW1PW91/6-311+G(2d,p)//B3LYP/6-31G(d,p). [c]Calculated at B3LYP/6-31G(d,p)//B3LYP/6-31G(d,p). [d]Mean absolute error (ppm) against experimental ^{13}C chemical shifts of **6a**. [e]Mean absolute error (ppm) against experimental ^{13}C chemical shifts of **6b**. [f]Mean squared error against experimental ^{13}C chemical shifts of **6a**. [g]Mean squared error against experimental ^{13}C chemical shifts of **6b**. [h]DP4 probability (%) for **6a**. [i]DP4 probability (%) for **6b**.

Table S16. Comparison of experimental and calculated ^{13}C NMR chemical shifts for euthailol H (**7**, albireticulone A)

	δ_C exp[a]	δ_C calc (mPW1PW91)[b]		δ_C calc (B3LYP)[c]	
no.	**7**	(7*R*)-**7**	(7*S*)-**7**	(7*R*)-**7**	(7*S*)-**7**
1	47.5	47.01233	48.48523	47.65288	49.15067
2	132.2	138.10145	136.58099	136.65242	134.74128
3	134.4	136.88571	137.69146	134.38963	135.71264
4	34.4	38.08489	38.50484	37.80998	38.72033
5	40.4	40.91743	42.82168	39.92215	42.62245
6	219.9	218.93868	223.40248	218.27462	223.44789
7	47.1	44.73309	53.57992	43.85522	53.91331
8	35.3	35.75945	36.65779	36.08258	37.44352
9	30.7	31.91188	32.89441	32.11783	33.61784
10	46.7	47.58808	51.02425	48.32053	52.11942
11	40.2	41.10383	40.68556	40.81637	40.46949
12	31.5	29.71175	30.45601	29.36724	30.16376
13	30.8	30.11048	30.68897	29.92502	30.28767
14	45.7	46.93044	47.18498	46.61272	46.98721
15	148.5	154.43081	153.18922	149.48681	148.54441
16	113.2	114.50481	115.44269	113.01658	113.92331
17	26.8	26.89215	26.28851	25.69404	25.17375
18	21	19.58471	19.60277	19.64499	19.73185
19	17.5	18.76008	18.80140	18.69619	18.85060
20	18.9	14.76416	15.34273	13.60318	14.57275
MAE[d]		1.89	2.49	1.59	2.28
MSE[e]		2.53	3.00	2.12	2.87
DP4[f]		19.112	80.888	96.679	3.321

[a]Recorded in CD_3OD at 150.94 MHz (298K). [b]Calculated at mPW1PW91/6-311+G(2d,p)//B3LYP/6-31G(d,p). [c]Calculated at B3LYP/6-31G(d,p)//B3LYP/6-31G(d,p). [d]Mean absolute error (ppm). [e]Mean squared error. [f]DP4 probability (%).

Table S17. Comparison of experimental and calculated ^{1}H NMR chemical shifts for euthailol H (**7**, albireticulone A)

	δ_H exp[a]	δ_H calc (mPW1PW91)[b]		δ_H calc (B3LYP)[c]	
no.	**7**	(7*R*)-**7**	(7*S*)-**7**	(7*R*)-**7**	(7*S*)-**7**
1	2.1	2.011429	2.029441	2.122594	2.152380
2	5.47	5.499559	5.345386	5.765075	5.641795
4	2.27	2.376085	2.034291	2.350822	2.004765
4	2.53	2.648456	2.913001	2.482171	2.877579
5	2.26	2.476569	2.246371	2.381962	2.209109
5	3.05	2.771558	2.899570	2.787925	2.839937
7	2.95	3.080371	2.520404	3.178829	2.507863
8	1.34	1.017518	1.485820	1.115188	1.550076
8	1.84	2.091510	1.769369	2.226924	1.931426
9	0.71	0.930351	0.788690	1.166064	1.018964
9	1.31	1.249117	1.401178	1.398441	1.556736
10	1.02	0.918338	1.101090	1.237658	1.350158
11	1.12	1.127402	1.039768	1.299900	1.208531
12	1.36	1.387416	1.388418	1.401335	1.396194
12	1.46	1.484993	1.453894	1.678044	1.625446
13	1.61	1.601382	1.584459	1.657938	1.644151
13	1.71	1.630843	1.681101	1.667888	1.697481
14	2.26	2.234070	2.220428	2.225020	2.208254
16	4.87	4.888222	4.882176	5.068797	5.051230
16	4.94	5.095271	5.071262	5.165309	5.136655
17	1.74	1.782413	1.771909	1.767506	1.769039
18	0.949	0.994038	0.966463	1.066726	1.020782
19	0.946	0.970313	0.898193	1.016464	0.948413
20	1.57	1.651394	1.786673	1.654977	1.781489
MAE[d]		0.103	0.106	0.155	0.159
MSE[e]		0.137	0.152	0.194	0.199
DP4[f]		87.229	12.771	98.789	1.211

[a]Recorded in CD_3OD at 499.63 MHz (303K). [b]Calculated at mPW1PW91/6-311+G(2d,p)//B3LYP/6-31G(d,p). [c]Calculated at B3LYP/6-31G(d,p)//B3LYP/6-31G(d,p). [d]Mean absolute error (ppm). [e]Mean squared error. [f]DP4 probability (%)

Experimental Methods

General Materials

Oligonucleotide primers were synthesized by Microsynth AG (Mainz, Germany). DNA synthesis was conducted by Twist Bioscience (USA). Q5 High-Fidelity DNA polymerase, deoxynucleotides (dNTPs), restriction endonucleases, NEBuilder HiFi DNA assembly master mix, Monarch plasmid miniprep Kit, and Monarch DNA gel extraction kit were purchased from New England Biolabs (UK) and used according to the protocols provided by the manufacturer. Chemicals, media components and solvents were purchased from Carl Roth (Germany), BD Difco (UK) and Merck (Germany).

Molecular cloning

The native *eut* BGC or synthetic DNA were used as templates for PCR. Oligonucleotide primers used in this study are listed in Tables S3. All polymerase chain reactions (PCRs) were conducted on a C1000 Touch Thermal Cycler, Bio-Rad (USA) using Q5 High Fidelity DNA polymerase, according to the manufacturer's instructions. PCR products were verified by agarose gel electrophoresis and isolated using the kits mentioned above. DNA fragments obtained from the PCR reactions were treated with restriction endonuclease *Dpn*I prior to DNA assembly using NEB builder HiFi DNA assembly master mix. The DNA assembly reactions were then transformed into *E. coli* DH5α. Sanger and full plasmid sequencing were conducted by Microsynth AG (Mainz, Germany) to confirm the right constructs.

Bacterial strains and culture conditions

Plasmids harboring codon optimized *eutB* full length and its truncated versions were transformed into *E. coli* BL21 (DE3) containing the MVA pathway and a gene encoding a GGPS for supply of geranylgeranyl pyrophosphate. *E. coli* strains were grown in Luria Bertani (LB) broth or LB agar (10 g/L tryptone, 5 g/L yeast extract, 10 g/L NaCl, 15 g/L agar) with appropriate antibiotics (ampicillin 100 µg/mL). Each culture was grown at 37°C with shaking at 180 rpm until an OD_{600} of 0.4-0.6 was reached and then induced by adding 100 µM isopropyl β-D-1-Thiogalactopyranoside (IPTG). The cells were further cultured with 20% *n*-octane at 16°C for 18 h with shaking at 180 rpm.

Plasmids harboring the native *eut* BGC and partial sets of *eut* genes were introduced into *Streptomyces* host strains by conjugation via *E. coli* ET12567 (pUZ8002) as described previously.[3] Recombinant *Streptomyces* strains were grown on Mannitol-soy flour agar (MS agar) (20 g/L mannitol, 20 g/L soya flour, 20 g/L agar, and 10 mM $MgCl_2$) or ISP2 agar (4.0 g/L glucose, 4.0 g/L yeast extract and 10.0 g/L malt extract and 20 g/L agar) containing appropriate antibiotics (50 µg/mL Hygromycin-B, 50 µg/mL Apramycin and 25 µg/mL Nalidixic acid, final concentration) for 3-4 days at 30°C. Recombinant *Streptomyces* strains for the production of target terpenoids were cultured in tryptic soy broth medium (TSB) supplemented with hygromycin-B (50 µg/mL) and apramycin (50 µg/mL). All production cultures were incubated for 7 days at 30 °C with shaking at 180 rpm using a 5-liter flask containing 1 L liquid media.

Construction of *Streptomyces* heterologous host for terpene production

Genes encoding enzymes involved in the MVA pathway were amplified from the plasmid pUMV19 (from Addgene) (http://n2t.net/addgene:67161), and cloned into pIJ10257 under the strong constitutive *ermE* promoter. The constructed plasmid was then transformed into *E. coli* ET12567/pUZ8002 by electroporation followed by an intergenic conjugation into *Streptomyces albus* J1074, *Streptomyces coelicolor* M1154 *and Streptomyces avermitilis* SUKA22, to achieve a terpene producer with high precursor supply.

Protein modeling analysis of terpene cyclase and hypothetical protein

The predicted protein models for the Terpene cyclase, hypothetical protein and the cytochrome P450 complex were generated by using Alpha Fold2 Colab notebook[4,5] (https://colab.research.google.com/github/sokrypton/ColabFold/blob/main/AlphaFold2.ipynb) and models were analyzed with pymol.

To identify the potential binding site for a cofactor in the structure of EutC, we searched for putative conserved regions that might be responsible for cofactor binding. Putative binding pockets were predicted by DeepSite[6] analysis using the default settings. FAD, NAD and heme were modeled in EutC using AlphaFold 3.[7] Conserved regions were identified using DeepSite on default settings. ScanProsite[8] was used on default settings to detect prosite signatures.

Comparison of BGC of interest with similar gene clusters

To query similar gene clusters within GenBank, the CAGECAT web server was utilized on default settings on 12.12.2023.[9] Additionally, the NPDC database was queried on 12.12.2023 for similar clusters using the hypothetical protein as bait for the DIAMOND-BLASTP with e-value, %identity, and query coverage cutoffs of 1e-10, 40%, and 80%, respectively.[10] Similar gene clusters were manually selected based on cluster architecture. To visualize and compare the gene clusters, clinker was used with a sequence similarity threshold of 0.3.[11] The nucleotide sequence of the *eut* BGC has been deposited in the GenBank database under the accession number *PQ240601.*

Genome sequencing and phylogenetic analysis

Genomic DNA was extracted from early stationary phase cultures of *S. euthainensis* following the protocol outlined by Nikodinovic *et al.*[12] Genome sequencing was conducted using the PacBio Sequel platform, with the sequencing library prepared using the SMRTbell™ Template Prep Kit and Sequel™ Binding Kit 2.0 according to manufacturer's description. The raw sequence reads were de novo assembled using HGAP2 (Pacific Biosciences, Menlo Park, CA). Gene prediction was performed with Prodigal,[13] while secondary metabolite biosynthetic gene clusters were annotated using antiSMASH.[14] The genome was determined to have a BUSCO completeness score of 99.9% and a size of 7.3 Mb with a GC content of 72.2%, as analyzed with Geneious Prime 2024.0.

The assembled genomic sequence was submitted to the Type (Strain) Genome Server (TYGS) of Deutsche Sammlung für Mikroorganismen und Zellkulturen (DSMZ).[15] The analysis was run under default conditions and without the preselection of particular type strains. The TYGS compared the query genome against all type strain genomes available in TYGS database (>20k strains) using the MASH algorithm and selected the best matching strains.[16] The phylogenomic inference was determined by pairwise comparisons among the set of genomes using the Genome BLAST Distance Phylogeny approach (GBDP) and the intergenomic distances were calculated by under the algorithm 'trimming' and distance formula d_5.[17] Digital DNA: DNA hybridization (dDDH) values and confidence intervals were calculated using the recommended settings of the GGDC 4.0.[17,18]

Culture conditions and LC-MS analysis of recombinant strains

Recombinant *Streptomyces* strains harboring plasmids with full length eutBGC (*S. avermitilis* SUKA22, S. *albus* J1074 and *S. coelicolor* M1154) and recombinant *S. albus* J1074 strains containing the plasmid with partial sets of *eut* genes were first cultured in TSB media with appropriate antibiotics (50 µg/mL hygromycin-B, 50 µg/mL apramycin) for 3 days for spore germination and then inoculated in 1 L ISP2 and TSB media respectively, using 5-liter baffled shake flasks. The cultures were incubated for 7 days at 30 ^{0}C at 180 rpm. After fermentation the cultures were extracted with equal culture volume of ethyl acetate and the crude extracts were dried under reduced pressure.

The crude extracts from each recombinant strain were analyzed by HPLC-MS using an Ultimate 3000 LC system (Thermo Fisher) coupled to an AmaZonX (Bruker) electro-spray ionization (ESI) ion trap mass spectrometer. Separation was achieved on a C18 column (ACQUITY UPLC BEH, 130 Å, 1.7 µm particle size, 2.1 × 100 mm, Waters) using acetonitrile and Milli-Q water supplemented with 0.1% (v/v) formic acid in a gradient ranging from 5 to 95% acetonitrile at a flow rate of 0.4 mL/min at 40 °C for 16 min. HPLC-ESI-QTOF-MS analyses were conducted on an Ultimate 3000 LC system (Thermo Fisher) coupled to an Impact II QTOF mass spectrometer (Bruker). Separation was achieved on a C18 column (ACQUITY UPLC BEH, 130 Å, 1.7 µm particle size, 2.1 mm × 100 mm, Waters) using acetonitrile and Milli-Q water supplemented with 0.1% (v/v) formic acid in a gradient ranging from 5 to 95% acetonitrile at a flow rate of 0.4 mL/min at 40 °C for 16 min. MS data was acquired in positive mode at a scan range between 100 to 1200 *m/z* for the detection of terpenoids and the measurements were evaluated with DataAnalysis 4.3 (Bruker).

Large scale production and purification of euthailols A–G

For the isolation of terpenoids associated with the prioritized pathway each *Streptomyces* recombinant strain was cultured in 5 mL TSB media containing 50 ug/ml of appropriate antibiotics (Hygromycin-B and apramycin) at 30 °C, 180 rpm. After 3 days of incubation 5 mL of seed culture was used to inoculate (10 x 1 L) TSB media containing 50 ug/mL of hygromycin-B and apramycin in 5 L-baffled Erlenmeyer flasks. The cultures were incubated for 7 days at 30 °C and 180 rpm. The cell cultures were harvested by centrifugation at 10,000 x g for 20 min and the cell free supernatant was extracted with equal volume of ethyl acetate. The crude extract was dried under reduced pressure in a Laborata 40003 control rotary evaporator.

The crude extracts of each recombinant strain were subjected to silica gel open column chromatography and eluted with petroleum ether–ethyl acetate (9:1, 7:3, 5:5, v/v). The resulting fractions were screened for the compounds of interest by LC-MS analysis, and the fractions containing the target compounds were combined and dried under reduced pressure. The dried fractions were dissolved in MeOH and, if needed, further purified by preparative HPLC on an Agilent 1260 Infinity II UV–vis system, equipped with Luna C18 preparative column (100 Å, 5 µm particle size, 250 x 21.2 mm, Phenomenex). Solvents used were Milli-Q water and acetonitrile as mobile phases A and B, respectively. A gradient was set from 50 to 95% solvent B over 30 min. and then 100% solvent B for 10 min., giving a total run time of 40 min., with a flow rate of 21 mL/min. Terpenoids that require further purification were obtained by semi-preparative HPLC on the Agilent 1260 Infinity II UV–vis system, equipped with Phenyl-hexyl RP column (100 Å, 5 µm particle size, 250 x 10 mm, Phenomenex). Milli-Q water and acetonitrile were used as mobile phases A and B, respectively. An isocratic method with 60% solvent B over 25 min and then a gradient of 60-100% solvent B for 10 min, with a flow rate of 3 mL/min was used. The solvent from purified fractions was evaporated under reduced pressure and then the compounds were dissolved in deuterated Acetone-d_6 and Methanol-d_4 for the NMR analysis. Optical rotation values of euthailols were measured on a Jasco P-2000 polarimeter, using a 3.5 mm × 10 mm cylindrical quartz cell.

Physical and spectroscopic properties

Euthailol A (**1**): white powder (1.5 mg); ^{1}H and ^{13}C NMR data, see Table S1; HRMS (ESI-QTOF) calc. for $C_{20}H_{33}O_3$ $[M + H]^+$ *m/z* 321.2424, found 321.2420.

Euthailol B (**2**): white powder (1.29 mg); ^{1}H and ^{13}C NMR data, see Table S2; HRMS (ESI-QTOF) calc. for $C_{20}H_{34}NaO_4$ $[M + Na]^+$ *m/z* 361.2355, found 361.2348.

Euthailol C (**3**): white powder (1.1 mg); ^{1}H and ^{13}C NMR data, see Table S3; HRMS (ESI-QTOF) calc. for $C_{20}H_{33}O_2$+ $[M + H]^+$ *m/z* 321.2424, found 305.2425.

Euthailol D (**4**): white powder (0.62 mg); $[\alpha]^{20}_{D}$ −50.0 (*c* 0.3, Acetone); ^{1}H and ^{13}C NMR data, see Table S4; HRMS (ESI-QTOF) calc. for $C_{20}H_{33}O_2$ $[M + H]^+$ *m/z* 305.2475, found 305.2470.

Euthailol E (**5**): white waxy solid (0.58); $[\alpha]^{20}_{D}$ −11.6 (*c* 0.51, Acetone); ^{1}H and ^{13}C NMR data, see Table S5; HRMS (ESI-QTOF) calc. for $C_{20}H_{33}O_2$ $[M + H]^+$ *m/z* 305.2475, found 305.2473.

Euthailol F (**6a**): colorless oil (0.7 mg); $[\alpha]^{20}_{D}$ +127.65 (*c* 0.23, Acetone); ^{1}H and ^{13}C NMR data, see Table S6; HRMS (ESI-QTOF) calc. for $C_{20}H_{33}O_2$ $[M + H]^+$ *m/z* 305.2475, found 305.2474.

Euthailol G (**6b**): colorless oil (0.36 mg); $[\alpha]^{20}_{D}$ −2.85 (*c* 0.35, Acetone); ^{1}H and ^{13}C NMR data, see Table S6; HRMS (ESI-QTOF) calc. for $C_{20}H_{33}O_2$ $[M + H]^+$ *m/z* 305.2475, found 305.2474.

Euthailol H (**7**, albireticulone A): white waxy solid (1.7 mg); $[\alpha]^{20}_{D}$ +6.6 (*c* 1.5, $CHCl_3$); ^{1}H and ^{13}C NMR data, see Table S7; HRMS (ESI-QTOF) calc. for $C_{20}H_{31}O$ $[M + H]^+$ *m/z* 289.2526, found 289.2526.

NMR analysis

^{1}H NMR, ^{13}C{^{1}H} NMR, HSQC, HMBC, ^{1}H–^{1}H COSY, NOESY and ROESY spectra were acquired on Bruker AV500 and DRX600 spectrometers. Chemical shifts (δ) were acquired in ppm with reference to the residual solvents of $CHCl_3$ (δ 7.26 for ^{1}H NMR), $CDCl_3$ (δ 77.16 for ^{13}C{^{1}H} NMR), $(CD_3)(CD_2H)CO$ (δ 2.05 for ^{1}H NMR),

$(CD_3)_2CO$ (δ 29.84 for $^{13}C\{^1H\}$ NMR), CD_2HOD (δ 3.31 for ^1H NMR), CD_3OD (δ 49.0 for $^{13}C\{^1H\}$ NMR), C_5D_4HN (δ 7.19 for ^1H NMR), and C_5D_5N (δ 123.5 for $^{13}C\{^1H\}$ NMR). Spectra were processed using TOPSPIN 3.6.2 (Bruker) and MestreNova 14.3.1 (Mestrelab).

Structure elucidation:

Euthailols A–C (1–3)

The molecular formulas of **1–3** were determined to be $C_{20}H_{32}O_3$, $C_{20}H_{34}O_4$, and $C_{20}H_{32}O_3$, respectively, based on HR-ESI-QTOF-MS data (Figure S8, S9, S10). The ^1H NMR spectra of **1–3** recorded in CD_3OD were highly similar except for four protons. The ^1H NMR spectrum for **1** revealed a doublet olefinic proton (δ_H 5.57 ppm), two singlet methyl groups (δ_H 1.57 and 1.47 ppm), one doublet methyl group (δ_H 0.97 ppm), and a pair of oxygenated methylene protons (doublets of doublets; δ_H 3.51 and 3.46 ppm). Six methylene protons, a hallmark of terpenoids, overlapped in the region of 1.50–1.66 ppm (δ_H 1.61. 1.55, and 1.51) and 1.22–1.35 (δ_H 1.30, 1.27, and 1.26) (Figure S11). The ^{13}C NMR spectrum showed the characteristic ketone group at 218 ppm and four carbons that are directly connected or close to hydroxy groups (δ_C 65.8, 60.7, 55.4, and 54.3) (Figure S12).

The structure of **1** was elucidated by building a total of four fragments: **I**–**III** around three methyl groups (C-17, C-18, and C-20) and **IV** oxygenated methylene group (C-19) through HMBC correlations. The fragments were then extended and connected through ^1H–^1H COSY and/or HMBC correlations. Fragment **I** (C-2, C-3, C-4 and C-20) was connected to fragment **II** (C-6, C-7, C-8, and C-19) through the C-4–C-5–C-6 linkage indicated by the HMBC correlations of C-4/C-5, C-4/C-6, and C-7/C-5, resulting in a [8,4,0] bicyclic carbon backbone and accounting for all but one double bond equivalent. Based on the molecular formula two additional oxygen atoms should be present, one of which should be either an internal ether or epoxy group. Two deshielded doublet protons at C-16 (δ_H 2.57 and 2.50) with a $^2J_{H,H}$ geminal coupling constant of 4.7 Hz suggested an epoxy group between C-15–C-16 (Figure S11-S15). The absence of the epoxide methylene signals at C-16 in **2** and **3** implied epoxide ring opening. In the case of **2**, epoxide ring opening resulted in the diol congener of **1**, while an additional pair of geminal olefinic protons (δ_H 5.30 and 5.18) in **3** suggested that **3** is the C-15 dehydration production of **2**. Changing the NMR solvent to the CD_3CN, revealed individual hydroxy proton signals at C-16 and/or C-19 in the ^1H NMR spectrum that were masked in CD_3OD, confirming the position of the hydroxy and epoxide groups.

The stereochemistry of compounds **1**–**3** was elucidated through $^3J_{H,H}$ coupling constant analysis and NOESY correlations and is supported by density functional theory (DFT) calculations. The bridgehead H-1 was shown as a doublet of triplet with coupling constants of 11.0 and 5.5 Hz. Analysis of the coupling constants of the adjacent protons revealed *anti*-configuration on C-1/C-2 and C-1/C-10 and *syn*-configuration on C-1/C-14. NOE correlations of H-2/H_3-17 and H-2/H-10 supported these assignments. In addition, NOE correlation between H-2 and H-4 established the *E*-configuration of the methyl allyl moiety at C-2. NOE correlation between H-7 and H-2 suggested their cofacial relationship that both are pointing downwards. The bridgehead H-10 was shown as a triplet of triplets (only observed in **2**) with coupling constants of 11.2 and 3.0 Hz, with the 3.0 Hz triplet coupling likely caused by the H_2-9 protons on the flexible 10-membered ring and the 11.0 Hz coupling each comes from H-1 and H-11, suggesting *anti*-configuration between C10 and C11 (Table S1-S3). The relative configuration at C-15 was elusive based on NMR data. We therefore performed DFT calculations to predict the ^1H and ^{13}C NMR chemical shifts of both C-15 diastereoisomers of **1** (Table S1). The ^{13}C NMR chemical shifts of (15*R*)-**1** calculated at mPW1PW91 and B3LYP functionals were in good agreement with the experimental data, showing mean absolute errors (MAEs) of 2.04 and 2.08 ppm, respectively. Furthermore, the DP4 statistical analysis of ^{13}C NMR chemical shifts suggested the high probability of 15*R* isomer in both mPW1PW91 and B3LYP functionals. In similar manner, comparison of the calculated ^1H NMR chemical shifts of two possible diastereomers calculated at mPW1PW91/6-311+G(d,p) with experimental ^1H NMR chemical shifts revealed the relative configuration of C-15 as *R**. Although the ^1H NMR chemical shifts of (15*S*)-**1** calculated at B3LYP/6-31G(d,p) showed lower MAE and higher DP4 probability[19] than those of (15*R*)-**1**, a closer chemical shift of H-14, which is most likely affected by the deshielding effect from the epoxide moiety, was found in (15*R*)-**1**. Consequently, the relative configuration at C-15 in **1** was determined to be *R**.

Euthailol D (4)

The molecular formula of euthailol D (**4**) was determined to be $C_{20}H_{32}O_2$ based on a protonated ion at *m/z* 305.2470 $[M+H]^+$ (calc. for $C_{20}H_{33}O_2$, 305.2475) in HR-ESI-QTOF-MS data (Figure S32). The ^1H NMR spectrum (Figure S33) showed resonances responsible for two hydroxy protons (δ_H 6.43, 6.19), an *exo*-olefin (δ_H 5.60, 5.17), two

olefinic protons (δ_H 5.25, 5.10), an oxygenated methine (δ_H 4.91), an oxygenated methylene (δ_H 4.23), two allyl methyls (δ_H 1.56, 1.50), and an aliphatic methyl (δ_H 1.00). Analysis of the ^{13}C NMR spectra with the aid of HSQC correlations (Figures S34, S36) revealed six sp^2 carbons, four of which were unprotonated, two oxygenated sp^3 carbons, four aliphatic methines, five aliphatic methylenes, and three methyls. The ^1H–^1H COSY correlations of the aliphatic region showed a constitutive spin system, indicating the presence of a methyl cyclohexane ring that is connected with an olefin (δ_H 5.10, H-2) and an aliphatic methylene (δ_H 1.88, 1.04, H_2-9) and two methines (δ_H 2.14, H-1; δ_H 1.26, H-10), respectively (Figure S35, Table S4). An additional spin system in an olefin (δ_H 5.25, H-6), an oxygenated methine (δ_H 4.91 H-5), and aliphatic methylene (δ_H 2.71, 2.27, H-4) was also detected in the ^1H–^1H COSY spectrum. Analysis of the HMBC spectrum indicated the presence of a bicyclo[8.4.0]tetradecane ring system based on the cross-peaks from the allyl methyl (δ_H 1.56, H_3-20) to C-1 (δ_C 46.4) and C-4 (δ_C 50.3), and from the other allyl methyl (δ_H 1.50, H_3-19) to C-6 (δ_C 132.2) and C-8 (δ_C 41.4), along with cross-peaks from the aliphatic methylene H_2-9 to C-1 and C-11 (δ_C 37.2) (Figure S37, Table S4). Furthermore, the HMBC correlations from the *exo*-olefins H_2-17 to the oxygenated methylene C-16 (δ_C 66.7) and the aliphatic methine C-14 (δ_C 41.4) indicated the presence of a hydroxy isopropenyl group that is located at C-14 of the bicyclic ring. The *E*-configuration of the methyl allyl moieties was deduced based on the W-typed long range ^1H–^1H COSY correlations between H-2 and H_3-20, as well as H-6 and H_3-19. The stereochemistry of **4** was not determined because of the low isolation yield.

Euthailol E (5)

The molecular formula of euthailol E (**5**) was determined to be $C_{20}H_{32}O_2$ based on the protonated ion at *m/z* 305.2473 $[M+H]^+$ (calc. for $C_{20}H_{33}O_2$, 305.2475) in HR-ESI-QTOF-MS data (Figure S38). The ^1H NMR chemical shifts of **5** in $CDCl_3$ were identical to those previously reported (Table S5, Figures S42).[20] Thus **5** was determined to be identical to 17-hydroxy albireticulone A. However, the relative configuration of C-7 remained ambiguous, as it was not conclusively determined from the NMR data, nor was it indicated in the previous literature.

Structure elucidation of Euthailol F (6a) and G (6b)

Compound **6a** has a molecular formula of $C_{20}H_{32}O_2$, which was determined based on a protonated ion at *m/z* 305.2474 $[M+H]^+$ (calc. for $C_{20}H_{33}O_3$, 305.2475) in HR-ESI-QTOF-MS data (Figure S39). The ^1H and ^{13}C NMR spectra, and HSQC data suggested the presence of three doublet methyls, one singlet methyl, seven methylenes, and six methine protons, one of which was connected to a sp^2 carbon (Figure S47–48, S50). COSY cross peaks revealed three fragments, units **a**–**c** (Figure S49). HMBC correlations from H-2 and H_3-20 to C-4, from H_2-4 to C-2, C-3, and C-20, and from H_2-5 to C-3 revealed that C-3 in unit **b** was connected to C-4 in unit **c** (Figure S51). C-5 in unit **c** was connected to C-7 in unit **b** via a ketone C-6, which was determined by HMBC correlations from H_2-4, H_2-5, and H_3-19 to C-6. HMBC correlation from H_3-18 to C-10 determined the connection between C-11 in unit **a** and C-10 in unit **b**. The methyl group C-17 was connected to C-14 via an epoxide C15–C16, which was revealed by HMBC correlations from H_3-17 to C-15, C-16, and C-14, from H_2-16 to C-17 and C-15, and from H-1 to C-15. Finally, C-14 in unit **b** and C-13 in unit **a** were connected by HMBC correlations from H_2-13 to C-2 and C-4 to complete the structure of **6a**. The double bond C2–C3 was determined to be *E*-configured based on the NOESY cross peak between H-2 and H-4 (Figure S57). The *anti*-relationship of H-1 and H-10 was determined by NOESY cross peaks between H-1 and H-9, and between H-10 and H-2. NOESY cross peaks between H_3-17 and H-2, between H-16 and H-2, and between H-16 and H-10 revealed the *syn*-relationship of C-1 and C-14 as shown in Table S6. The coupling constants of H-1 also supported the *anti-syn* relationship of H-10, H-1 and H-14 (Table S6). The NOE correlations of H-1/H-13 and H-11/H-13 indicated their cofacial relationships. Consequently, the relative configuration of **6a** was determined as 1*S**,10*R**,11*S**,14*R**. Compound **6b** has the same molecular formula as **6a**. The interpretation of NMR data including ^1H NMR spectrum, HSQC, COSY, and HMBC revealed that the planar structure was identical to **6a** (Figure S53–S57, Table S6). The geometry of the double bond C11–C12 was also the same as that of **6a**, which was determined by the NOESY cross peak between H-2 and H-4 (Figure S57). The relative configuration at bridgeheads C-1 and C-10, as well as two methines C-11 and C-14 were determined to be the same as those of **6a** based on NOE correlations (Table S6). These NMR results of **6a** and **6b** revealed that they are diastereomers with the different stereochemistry at C-15 and/or C-7, but these relative configurations remain uncharacterized by only using NMR data. To determine the stereochemistry at C-15 and C-7, ^1H and ^{13}C NMR chemical shifts were calculated using the GIAO method. Significant low MAEs for calculated ^1H and ^{13}C NMR chemical shifts at both mPW1PW91 and B3LYP functionals were found in (7*R*,15*R*)-**6** and (7*R*,15*S*)-**6** (Table S14 and S15). Furthermore, the calculated chemical shifts of C-7 and H-7 in both (7*R*,15*R*)-**6** and (7*R*,15*S*)-**6** were closer to those experimental chemical shifts than (7*S*,15*R*)-**6** and (7*S*,15*S*)-**6**, indicating relative configurations at C-7 for **6a** and **6b** as *R**. The relative configuration at C-15, however, was still

elusive because the calculated chemical shifts of (7*R*,15*R*)-**6** were similar to both **6a** and **6b** rather than those of (7*R*,15*S*)-**6**, except for the ^{1}H NMR chemical shifts calculated at B3LYP/6-31G(d,p) of which (7*R*,15*R*)-**6** and (7*R*,15*S*)-**6** showed almost the same differences compared with experimental chemical shifts of **6a** and **6b**. Consequently, **6a** and **6b** were determined to be diastereomers of the epoxide at C-15.

Euthailol H (7, albireticulone A)

The molecular formula of euthailol H (**7**) was determined to be $C_{20}H_{30}O$ based on a protonated ion at *m/z* 289.2526 $[M+H]^+$ (calc. for $C_{20}H_{31}O$, 289.2526) in HR-ESI-QTOF-MS data (Figure S40). The ^{1}H NMR spectrum of **7** in $CDCl_3$ was identical to the one reported for albireticulone A (Table S7).[1,20] In the NOESY spectrum recorded in CD_3OD, NOE correlations of H-2/H-7, H-2/H-10, and H-2/H-16 were observed, suggesting their cofacial relationships and the relative configuration at C-7 as 7*R** (Figure 63). However, the stereochemical assignment at C-7 is alarmingly mismatched with the relative configuration that has been previously determined based on the NOE correlations and $^3J_{H,H}$ coupling constants. [1] The bicyclo [8.4.0] tetradecanone ring system of **7** is likely to be relatively flexible, suggesting that the determination of the stereochemistry at C-7 using only NOE correlations and coupling constant analysis is inconclusive. Thus, DFT calculations of the ^{1}H and ^{13}C NMR chemical shifts for 7*R*-**7** and 7*S*-**7** were carried out. The overall ^{1}H and ^{13}C NMR chemical shifts calculated at mPW1PW91/6-311+G(2d,p) or B3LYP/6-31G(d,p) for (7*R*)-**7** (MAEs ^{1}H 0.103 ppm, ^{13}C, 1.89 ppm for mPW1PW91 functional; ^{1}H 0.155 ppm, ^{13}C 1.59 ppm for B3LYP functional) were favored compared to those of (7*S*)-**7** (MAEs ^{1}H 0.106 ppm, ^{13}C 2.49 ppm for mPW1PW91 functional; ^{1}H 0.159 ppm, ^{13}C 2.28 ppm for B3LYP functional) (Table S16 and S17). Among others, high similarities of chemical shifts for (7*R*)-**7** to experimental chemical shifts were found in the stereocenter C-7 (H-7) with its adjacent carbons C-6 and C-8 whose chemical shifts are most likely associated with the stereochemistry of C-7. Moreover, the DP4 probability analyses[19] also supported the 7*R* configuration for **7**, except for the probability of ^{13}C NMR chemical shifts calculated at mPW1PW91 functional that showed (7*S*)-**7** to be favorable with a low confidence (80.9 %) (Table S16 and S17) This inverted probability might be arising from the high error chemical shifts at C-2 and C-15 of (7*R*)-**7**, both of which are not directly affected by the stereochemistry of C-7. Consequently, the relative configuration of C-7 was determined to be *R*, resulting in the structure revision of the relative configuration for albireticulone A from 7*S* to 7*R*.

Antimicrobial activity assay

Antimicrobial activity of all compounds extracted were tested by disk diffusion assay against a panel of Gram-positive and negative (ESKAPE) pathogens (*Enterococcus faecium, Straphylococcus aureus, Klebsiella pneumonia, Actinetobacter baumannii, Pseudomonas aeruginosa, Enterobacter cloacae*). *Candida albicans* CAF4-2 was used to test antifungal activity. The pathogenic strains were cultured in LB medium at 37 °C, except for *Candida albicans* that was cultured in potato dextrose broth (PDB) at 30 °C, 130 rpm. The cultures were then diluted to an OD_{600} of 0.05 with LB agar for the disk diffusion susceptibility test. Sterile paper discs (6 mm diameter) with 5 µL (2.56 mg/mL) of purified diterpenoids were placed on top of each agar plate. Trimethoprim and ampicillin were used as positive controls for the antibacterial assays. Nystatin and cycloheximide were used as positive control for antifungal assays. The plates were incubated at 37 °C for 16 h (ESKAPE pathogens) or at 30 °C for 24 h (*C. albicans* CAF-2). The antimicrobial activity was determined by the size of inhibition zones formed on the agar plates. The broth microdilution method was used to determine the MIC value of the bioactive compounds.[21] Overnight cultures of each test strain were diluted with MHB medium to a concentration of 5×10^5 CFU/mL and transferred to 96-well plates. The cells were then treated with twofold serial dilutions of the purified compounds **4-7**. Each well contained a total volume of 200 µL, with final compound concentrations of 256, 128, 64, 32, 8, 4, and 2 µg/mL. Negative control wells contained only bacterial cells, while positive control wells contained ampicillin at 256 µg/mL. Plates for *Arthrobacter pascens* were incubated at 30°C with shaking at 120 rpm for 3-5 days. For all ESKAPE strains, plates were incubated at 37°C with shaking at 140 rpm for 16 hours. After incubation, the minimum inhibitory concentration (MIC) was determined as the lowest compound concentration that inhibited 90% of bacterial growth.

DFT Calculations

The 3-dimensional structures were modeled in the Spartan modeling software. The geometry of the models was minimized using molecular mechanics with the Spartan MMFF force field and subsequently subjected to conformational search using molecular mechanics again with the MMFF force field. For each structure, the ten conformers with the lowest energy as determined by MMFF, were selected for DFT calculations. All DFT calculations were performed with Gaussian 16. The geometries of all structures were first minimized using the B3LYP function with a 6-31G(d,p) basis set and in the gas phase and convergence was confirmed with a frequency calculation. If the minimization had not converged, minimization was repeated until convergence. We then performed NMR GIAO calculations with two different basis sets: B3LYP/6-31G (d,p) and mPW1PW91/6-311+G(2d,p), both in the gas phase. Calculated chemical shifts for ^{1}H and ^{13}C for each structure were determined using regression coefficients from the CHESHIRE online repository using the standard method.[10] We then averaged over the ten predicted spectra using a Boltzmann-weighted average.

References

1. Z. Li, B. Xu, T. A. Alsup, X. Wei, W. Ning, D. G. Icenhour, M. A. Ehrenberger, I. Ghiviriga, B.-D. Giang and J. D. Rudolf, Cryptic Isomerization in Diterpene Biosynthesis and the Restoration of an Evolutionarily Defunct P450, *J. Am. Chem. Soc.*, 2023, **145**, 22361-22365.
2. G.-M. Lin and C. A. Voigt, Design of a redox-proficient *Escherichia coli* for screening terpenoids and modifying cytochrome P450s, *Nat. Catal.*, 2023, **6**, 1016-1029.
3. L. Du, R.-H. Liu, L. Ying and G.-R. Zhao, An Efficient Intergeneric Conjugation of DNA from *Escherichia coli* to Mycelia of the Lincomycin-Producer *Streptomyces lincolnensis*, *Int. J. Mol. Sci.*, 2012, **13**, 4797-4806.
4. E. Richard, O. N. Michael, P. Alexander, A. Natasha, S. Andrew, G. Tim, Ž. Augustin, B. Russ, B. Sam, Y. Jason, R. Olaf, B. Sebastian, Z. Michal, B. Alex, P. Anna, C. Andrew, T. Kathryn, J. Rishub, C. Ellen, K. Pushmeet, J. John and H. Demis, Protein complex prediction with AlphaFold-Multimer, *bioRxiv*, 2022, DOI: 10.1101/2021.10.04.463034.
5. J. Jumper, R. Evans, A. Pritzel, T. Green, M. Figurnov, O. Ronneberger, K. Tunyasuvunakool, R. Bates, A. Žídek, A. Potapenko, A. Bridgland, C. Meyer, S. A. A. Kohl, A. J. Ballard, A. Cowie, B. Romera-Paredes, S. Nikolov, R. Jain, J. Adler, T. Back, S. Petersen, D. Reiman, E. Clancy, M. Zielinski, M. Steinegger, M. Pacholska, T. Berghammer, S. Bodenstein, D. Silver, O. Vinyals, A. W. Senior, K. Kavukcuoglu, P. Kohli and D. Hassabis, Highly accurate protein structure prediction with AlphaFold, *Nature*, 2021, **596**, 583-589.
6. J. Jiménez, S. Doerr, G. Martínez-Rosell, A. S. Rose and G. De Fabritiis, DeepSite: protein-binding site predictor using 3D-convolutional neural networks, *Bioinformatics*, 2017, **33**, 3036–3042.
7. J. Abramson, J. Adler, J. Dunger, R. Evans, T. Green, A. Pritzel, O. Ronneberger, L. Willmore, A. J. Ballard, J. Bambrick, S. W. Bodenstein, D. A. Evans, C.-C. Hung, M. O'Neill, D. Reiman, K. Tunyasuvunakool, Z. Wu, A. Žemgulytė, E. Arvaniti, C. Beattie, O. Bertolli, A. Bridgland, A. Cherepanov, M. Congreve, A. I. Cowen-Rivers, A. Cowie, M. Figurnov, F. B. Fuchs, H. Gladman, R. Jain, Y. A. Khan, C. M. R. Low, K. Perlin, A. Potapenko, P. Savy, S. Singh, A. Stecula, A. Thillaisundaram, C. Tong, S. Yakneen, E. D. Zhong, M. Zielinski, A. Žídek, V. Bapst, P. Kohli, M. Jaderberg, D. Hassabis and J. M. Jumper, Accurate structure prediction of biomolecular interactions with AlphaFold 3, *Nature*, 2024, **630**, 493–500.
8. E. de Castro, C. J. A. Sigrist, A. Gattiker, V. Bulliard, P. S. Langendijk-Genevaux, E. Gasteiger, A. Bairoch and N. Hulo, ScanProsite: detection of PROSITE signature matches and ProRule-associated functional and structural residues in proteins, *Nucleic Acids Res.*, 2006, **34**, W362–W365.
9. M. van den Belt, C. Gilchrist, T. J. Booth, Y.-H. Chooi, M. H. Medema and M. Alanjary, CAGECAT: The CompArative GEne Cluster Analysis Toolbox for rapid search and visualisation of homologous gene clusters, *BMC Bioinf.*, 2023, **24**, 181.
10. M. W. Lodewyk, M. R. Siebert and D. J. Tantillo, Computational Prediction of ^{1}H and ^{13}C Chemical Shifts: A Useful Tool for Natural Product, Mechanistic, and Synthetic Organic Chemistry, *Chem. Rev.*, 2012, **112**, 1839-1862.
11. C. L. M. Gilchrist and Y.-H. Chooi, clinker & clustermap.js: automatic generation of gene cluster comparison figures, *Bioinformatics*, 2021, **37**, 2473-2475.

12. J. Nikodinovic, K. D. Barrow and J.-A. Chuck, High yield preparation of genomic DNA from Streptomyces, *Biotechniques*, 2003, **35**, 932–936.
13. D. Hyatt, G.-L. Chen, P. F. LoCascio, M. L. Land, F. W. Larimer and L. J. Hauser, Prodigal: prokaryotic gene recognition and translation initiation site identification, *BMC Bioinf.*, 2010, **11**, 119.
14. K. Blin, S. Shaw, K. Steinke, R. Villebro, N. Ziemert, S. Y. Lee, M. H. Medema and T. Weber, antiSMASH 5.0: updates to the secondary metabolite genome mining pipeline, *Nucleic Acids Res.*, 2019, **47**, W81–W87.
15. J. P. Meier-Kolthoff and M. Göker, TYGS is an automated high-throughput platform for state-of-the-art genome-based taxonomy, *Nat. Commun.*, 2019, **10**, 2182.
16. B. D. Ondov, T. J. Treangen, P. Melsted, A. B. Mallonee, N. H. Bergman, S. Koren and A. M. Phillippy, Mash: fast genome and metagenome distance estimation using MinHash, *Genome Biol.*, 2016, **17**, 132.
17. J. P. Meier-Kolthoff, A. F. Auch, H.-P. Klenk and M. Göker, Genome sequence-based species delimitation with confidence intervals and improved distance functions, *BMC Bioinf.*, 2013, **14**, 60.
18. J. P. Meier-Kolthoff, J. S. Carbasse, R. L. Peinado-Olarte and M. Göker, TYGS and LPSN: a database tandem for fast and reliable genome-based classification and nomenclature of prokaryotes, *Nucleic Acids Res.*, 2022, **50**, D801-D807.
19. S. G. Smith and J. M. Goodman, Assigning Stereochemistry to Single Diastereoisomers by GIAO NMR Calculation: The DP4 Probability, *J. Am. Chem. Soc.*, 2010, **132**, 12946-12959.
20. Y. L. Hu, Q. Zhang, S. H. Liu, J. L. Sun, F. Z. Yin, Z. R. Wang, J. Shi, R. H. Jiao and H. M. Ge, Building *Streptomyces albus* as a chassis for synthesis of bacterial terpenoids, *Chem. Sci.*, 2023, **14**, 3661-3667.
21. I. Wiegand, K. Hilpert and R. E. W. Hancock, Agar and broth dilution methods to determine the minimal inhibitory concentration (MIC) of antimicrobial substances, *Nat. Protoc.*, 2008, **3**, 163–175.
